# Antimicrobial Dihydroflavonols and Isoflavans Isolated from the Root Bark of *Dalbergia gloveri*

**DOI:** 10.1021/acs.jnatprod.4c00690

**Published:** 2024-09-10

**Authors:** Ivan Kiganda, Lianne H. E. Wieske, Vaderament-Alexe Nchiozem-Ngnitedem, Duncan Chalo, Daniel Umereweneza, Albert Ndakala, Wouter Herrebout, Ruisheng Xiong, Tomasz M. Karpiński, Abiy Yenesew, Mate Erdelyi

**Affiliations:** † Department of Chemistry, University of Nairobi, P.O. BOX 30197, 30197-00100 Nairobi, Kenya; ‡ Department of Chemistry − BMC, Uppsala University, SE-751 23 Uppsala, Sweden; Δ Department of Biology, University of Nairobi, P.O. BOX 30197, 30197-00100 Nairobi, Kenya; § Department of Medical Microbiology, Poznań University of Medical Sciences, Rokietnicka 10, 60-806 Poznań, Poland; ¥ Departmnet of Chemistry, College of Science and Technology, University of Rwanda, P.O. Box 3900, Kigali, Rwanda; ∥ Department of Chemistry, University of Antwerp, 2020 Antwerp, Belgium

## Abstract

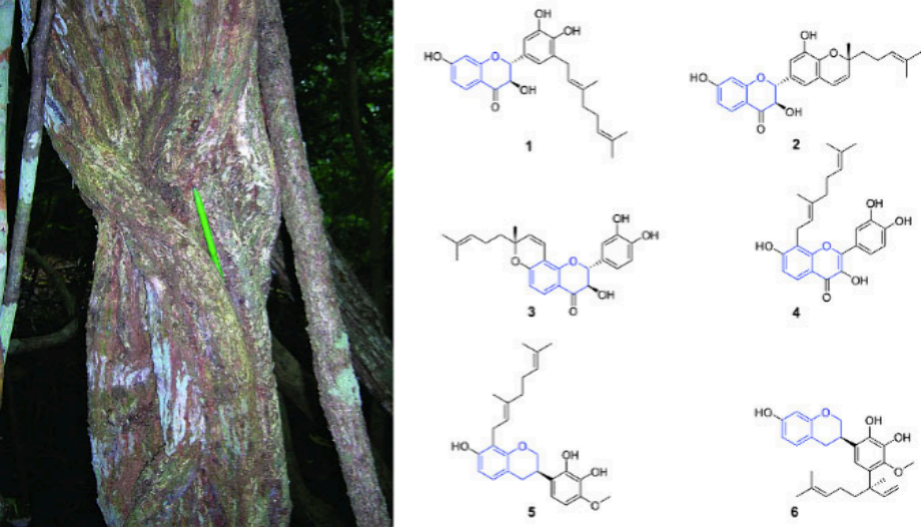

Three new dihydroflavonols, gloverinols A–C (**1**–**3**), a new flavon-3-ol, gloverinol D (**4**), two new isoflavans, gloveriflavan A (**5**) and B (**6**), and seven known compounds were isolated from the root bark of *Dalbergia gloveri*. The structures of the isolates were elucidated by using NMR, ECD, and HRESIMS data analyses. Among the isolated compounds, gloverinol B (**2**), gloveriflavan B (**6**), and 1-(2,4-dihydroxyphenyl)-3-hydroxy-3-(4-hydroxyphenyl)-1-propanone (**10**) were the most active against *Staphylococcus aureus*, with MIC values of 9.2, 18.4, and 14.2 μM, respectively.

The genus *Dalbergia* (family Fabaceae) comprises medium-sized trees, shrubs, and lianas widely distributed in Africa, Southern Asia, Central America, and Madagascar.^1^ Several *Dalbergia* species are known for their traditional medicinal uses. For example, *D. sissoo* is used in the treatment of syphilis, ulcers, dysentery, and skin diseases, and *D. latifolia* as a remedy to treat leprosy, diarrhea, and obesity in India, where they are native.^2^ In Senegal, the smoke of *D. melanoxylon* (Guill. & Perr.) stems and roots is inhaled to treat malaria, colds, headaches, bronchitis, and rheumatism.^3^*Dalbergia* species show a wide range of biological activities. For instance, *D. oderifera* possesses antibacterial, anti-inflammatory, antiallergic, and antioxidant properties, while *D. oliveri* presents antifungal, and *D. sissoo* osteogenic activities.^4^ This genus is known for providing a variety of sterols, anthraquinones, terpenes, cinnamyl esters, and flavonoids.^5^

*Dalbergia gloveri* Q. Luke. ined. is an endemic liana distributed in the coastal forest of Kenya at 30–320 m above sea level.^6^ It is an endangered species^7^ without any report on its phytochemistry or biological activity. In continuation of our interest in the phytochemistry of plants belonging to the genus *Dalbergia*,^4,8^ we report herein the isolation and characterization of six new (**1**–**6**) and seven known (**7**–**13**) compounds from the root bark of *D. gloveri*, along with the antimicrobial activity of the isolated compounds against *Staphylococcus aureus*, *Escherichia coli*, and *Candida albicans*.
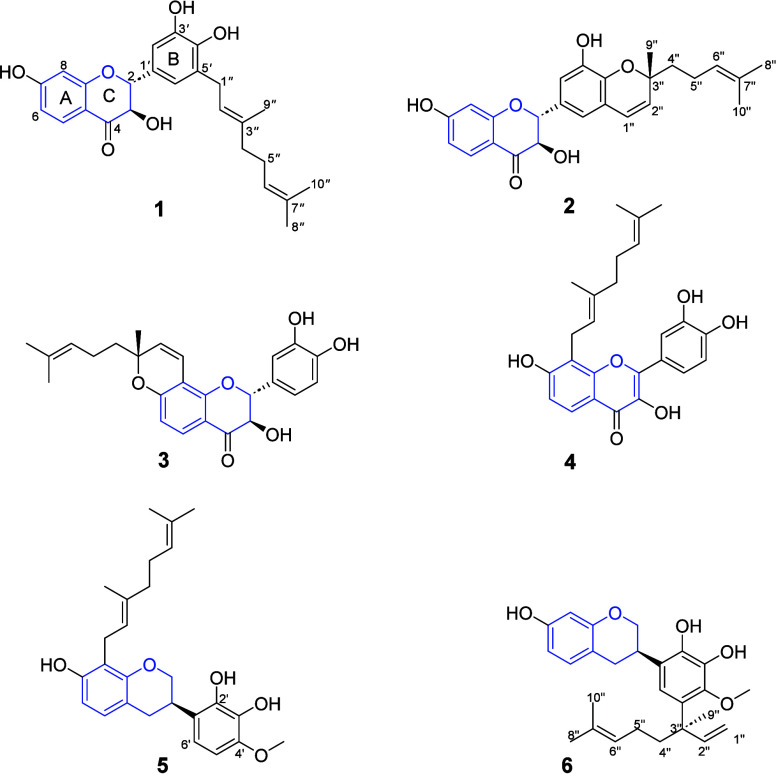


## Results and Discussion

The CH_2_Cl_2_/MeOH (1:1) extract of the root bark of *D. gloveri* was subjected to silica gel column chromatography, followed by purification on Sephadex LH-20 and preparative TLC to afford three new dihydroflavonols (**1**–**3**), a new flavon-4-ol (**4**), two new isoflavans (**5** and **6**), and the known secondary metabolites nitidulin (**7**),^9^ lespeol (**8**),^10^ isoliquiritigenin (**9**),^11^ 1-(2,4-dihydroxyphenyl)-3-hydroxy-3-(4-hydroxyphenyl)-1-propanone (**10**),^12,13^ dalbinol (**11**),^14,15^ (2*R*)-1,2-dihydro-2-[1-(hydroxymethyl)ethenyl]-8,9-dimethoxy[1]benzopyrano[3,4-*b*]furo[2,3-*h*][1]benzopyran-6(12*H*)-one (**12**),^16^ and oleanolic acid acetate (**13**),^17^ which were identified by comparison of their spectroscopic data with literature values.

**Figure 1:**
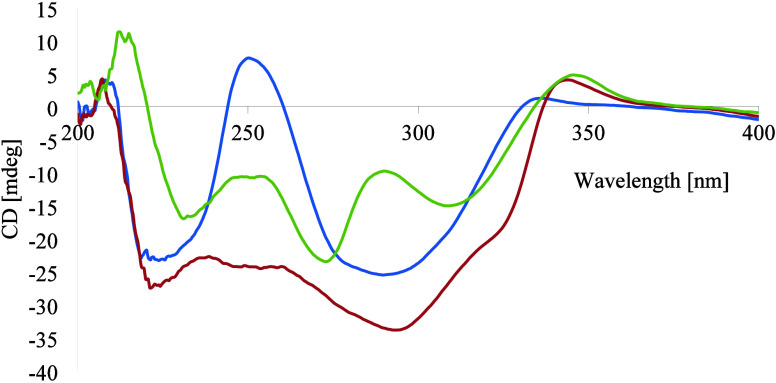
ECD spectra (in MeOH) of gloverionols A (**1**, blue), B (**2**, red), and C (**3**, lime green).

Gloverinol A (**1**) was isolated as a yellow, amorphous solid. Its HREIMS ([M + H]^+^*m*/*z* 425.1974, C_25_H_29_O_6_ calcd 425.1964) along with its NMR spectroscopic data (Table 1 and Figures S1–S9, Supporting Information) were consistent with 12 degrees of unsaturation. The UV (λ_max_ 279 and 309 nm) and ^1^H NMR spectroscopic data, which showed two mutually coupled protons at δ_H_ 4.91 (d, *J* = 11.6 Hz, H-2) and 4.46 (d, *J* = 11.6 Hz, H-3), along with the ^13^C NMR data at δ_C_ 85.9 (C-2), 74.5 (C-3), and 194.4 (C=O) were characteristic for a dihydroflavonol backbone.^18,19^ This was supported by the HMBC (Table 1, Figure S5, Supporting Information) correlations of δ_H_ 4.91 (H-2) with δ_C_ 129.4 (C-1*′*), 113.2 (C-2*′*), 74.5 (C-3), 194.4 (C-4), 121.4 (C-6*′*), and 165.1 (C-8a), as well as δ_H_ 4.46 (H-3) with δ_C_ 129.4 (C-1*′*), 85.9 (C-2), and 194.4 (C-4). The ^1^H NMR spectrum indicated five aromatic protons, of which three are in ring A displaying an AMX spin system [δ_H_ 7.72 (d, *J* = 8.7 Hz, H-5), 6.52 (dd, *J* = 8.7, 2.3, Hz, H-6), and 6.32 (d, *J* = 2.3 Hz, H-8)], with the biogenetically expected oxygenation at C-7 (δ_C_ 167.1). In ring B, *meta*-coupled protons at δ_H_ 6.84 (d, *J* = 2.1 Hz, H-2*′*) and 6.76 (d, *J* = 2.1 Hz, H-6*′*) indicated that this ring is trisubstituted at C-3*′*, C-4*′*, and C-5*′*, with two hydroxy groups at C-3*′* and C-4*′* and a C_10_ substituent at C-5*′*. The NMR spectroscopic data established that the substituent at C-5*′* (δ_C_ 129.1) is a geranyl group [δ_H_/δ_C_ 5.35 (m, H-2*′*)/123.9 (C-2*′*); 5.11 (m, H-6*′*)/125.4 (C-6*′*); 3.34 (d, *J* = 7.2 Hz, H-1*′*)/29.1 (C-1*′*); 2.10 (t, *J* = 7.4 Hz, H-5*′*)/27.7 (C-5*′*); 2.03 (t, *J* = 7.4 Hz, H-4*′*)/40.9 (C-4*′*); 1.72 (s, H-9*′*)/16.2 (C-9″); 1.62 (s, H-10*′*)/25.9 (C-10*′*); 1.57 (s, H-8*′*)/17.8 (C-8*′*)]. The substitution pattern in ring B was established based on the HMBC correlations of δ_H_ 3.34 (H-1*″*) with δ_C_ 129.4 (C-1′), 144.9 (C-4*′*), 129.1 (C-5*′*), 121.4 (C-6*′*), 123.9 (C-2″), and 136.8 (C-3*″*). The location of the geranyl unit was further confirmed by the HMBC correlation between the signal at δ_H_ 6.76 (H-6*′*) and δ_C_ 29.1 (C-1″), 85.9 (C-2), 113.2 (C-2*′*), and 144.9 (C-4*′*), Thus, compound **1** possesses a tetrasubstituted ring B similar to alnifoliol.^20^ The relative configuration around ring C of this dihydroflavonol (**1**) was determined based on the large ^3^*J*_H-2,H-3_ = 11.6 Hz coupling constant that is consistent with a 2,3-*trans* configuration and thus a 2*S*,3*S* or 2*R*,3*R* absolute configuration. According to Slade et al.,^21^ the naturally commonly occurring (2*R*,3*R*) isomer has a positive Cotton effect (CE) at ca. 300–340 nm, which corresponds to the forbidden π → n* transition. The weak positive CE at 332 nm (Figure 1) observed for **1** suggests it to have a 2*R*,3*R* absolute configuration.^21^ The strong negative CE observed at 295 nm, which corresponds to a π → π* transition and is thereby more reliable, is also consistent with a 2*R*,3*R*-configured dihydroflavonol.^22,23^ This conclusion is corroborated by the high negative specific rotation, [α]_D_^24^ −114 (*c* 0.2 M in CH_3_OH). Based on the above spectroscopic evidence, this new compound, gloverinol A (**1**), was characterized as (2*R*,3*R*)*-*3*′*,4*′*,7-trihydroxy-5*′*-geranylflavanon-3-ol.

**Table 1:** NMR Spectroscopic Data (^1^H 500 MHz and ^13^C 125 MHz, MeOH-*d*_4_) for Gloverinols A (**1**), B (**2**), and C (**3**)

	**1**			**2**			**3**		
position	δ_C_, type	δ_H_ m (*J* in Hz)	HMBC	δ_C_, type	δ_H_ m (*J* in Hz)	HMBC	δ_C_, type	δ_H_ m (*J* in Hz)	HMBC
2	85.9, CH	4.91 d (11.6)	C-1*′*, C-2*′*, C-3, C-4, C-6*′*, C-8a	85.5, CH	4.93 d (11.8)	C-1*′*, C-2*′*, C-3, C-4, C-4a, C-8a	85.7, CH	4.99 d (11.8)	C-3, C-4, C-1*′*, C-2*′*, C-6*′*
3	74.5, CH	4.46 d (11.6)	C-1*′*, C-2, C-4	74.5, CH	4.48 d (11.8)	C-1*′*, C-2, C-4, C-4a	74.5, CH	4.51 d (11.8)	C-2, C-4, C-1*′*
4	194.4, C			194.4, C			194.5, C		
4a	113.4, C			113.4, C			114.2, C		
5	130.1, CH	7.72 d (8.7)	C-4, C-7, C-8a	130.1, CH	7.73 d (8.7)	C-4, C-6, C-7, C-8, C-8a	128.9, CH	7.66 d (8.7)	C-4, C-7, C-8a
6	112.2, CH	6.52 dd (8.7, 2.3)	C-4a, C-8	112.2, CH	6.54 dd (8.7, 2.3)	C-4a, C-5, C-7, C-8, C-8a	112.2, CH	6.50 d (8.7)	C-4a, C-7, C-8
7	167.1, C			166.9, C			159.0, C		
8	103.7, CH	6.32 d (2.3)	C-4a, C-6, C-7	103.7, CH	6.35 d (2.3)	C-4, C-4a, C-6, C-7, C-8a	110.4, C		
8a	165.1, C			165.1, C			161.6, C		
1*′*	129.4, C			130.8, C			129.9, C		
2*′*	113.2, CH	6.84 d (2.1)	C-2, C-2*′*, C-4*′*, C-5*′*, C-6*′*	116.4, CH	6.89 d (2.1)	C-1*′*, C-2, C-3*′*, C-4*′*, C-5*′*, C-6*′*	115.9, CH	7.01 d (2.1)	C-1*′*, C-2, C-3*′*, C-4*′*, C-6*′*
3*′*	145.9, C			146.2, C			146.4, C		
4*′*	144.9, C			142.2, C			147.2, C		
5*′*	129.1, C			122.9, C			116.1, CH	6.82 d (8.1)	C-1*′*, C-3*′*, C-4*′*
6*′*	121.4, CH	6.76 d (2.1)	C-1″, C-2, C-2*′*, C-4*′*	118.3, CH	6.73 d (2.1)	C-2, C-2*′*, C-3*′*, C-4*′*, C-1″, C-5*′*	120.9, CH	6.89 dd (8.1, 2.1)	C-2, C-2*′*, C-4*′*, C-5*′*
1″	29.1, CH_2_	3.34 d (7.2)	C-1*′*, C-4*′*, C-5*′*, C-6*′*, C-2″, C-3″	123.9, CH	6.40 d (9.9)	C-1*′*, C-2″, C-3″, C-3*′*, C-4*′*, C-4″, C-5*′*, C-6*′*, C-9″	117.0, CH	6.60 d (10.2)	C-3″, C-7, C-8, C-8a, C-9″
2″	123.9, CH	5.35 m	C-1″, C-4″, C-9″	131.2, CH	5.67 d (9.9)	C-1*′*, C-1″, C-2*′*, C-3″, C-3*′*, C-4*′*, C-4″, C-5*′*, C-9″	129.3 CH	5.62 d (10.2)	C-8, C-8a, C-5*′*, C-3″, C-4″, C-9″
3″	136.8, C			80.3, C			81.5, C		
4″	40.9, CH_2_	2.03 m	C-2″, C-3″, C-5″, C-6″, C-9″	42.1, CH_2_	1.79 m	C-2″, C-3″, C-5″, C-6″, C-9″	42.7, CH_2_	1.74 m	C-2″, C-3″, C-5″, C-6″, C-9″
					1.67 ddd (10.0, 7.1, 7.1)	C-2″, C-3″, C-4*′*, C-5″, C-6″, C-9″		1.68 m	C-2″, C-3″, C-5″, C-6″, C-9″
5″	27.7, CH_2_	2.10 m	C-3″, C-4″, C-6″, C-7″	23.9, CH_2_	2.15 m	C-3″, C-4″, C-6″, C-7″, C-8″, C-10″	23.9, CH_2_	2.10 m	C-4″, C-6″, C-7″
6″	125.4, CH	5.11 m	C-5″, C-8″, C-10″	125.3, CH	5.12 m	C-4″, C-5″, C-8″, C-10″	125.0, CH	5.11 m	C-5″, C-8″, C-10″
7″	132.2, C			132.5, C			132.7, C		
8″	25.9, CH_3_	1.62 s	C-4″, C-6″, C-7″, C-10″	25.9, CH_3_	1.66 s (1.4)	C-4″, C-5″, C-6″, C-7″, C-10″	25.8, CH_3_	1.64 s	C-6″, C-7″, C-10″
9″	16.2, CH_2_	1.72 s	C-2″, C-3″, C-4″	26.8, CH_3_	1.42 s	C-1″, C-2″, C-3″, C-4″, C-4″, C-5″	27.4, CH_3_	1.39 s	C-1″, C-2″, C-3″, C-4″, C-5″
10″	17.8, CH_3_	1.57 s	C-5″, C-6″, C-7″, C-8″	17.8, CH_3_	1.58 s (1.4)	C-4″, C-5″, C-6″, C-7″, C-8″	17.6, CH_3_	1.56 s	C-6″, C-7″, C-8″

Gloverinol B (**2**) was isolated as a yellow, amorphous solid. Its molecular formula was determined to be C_25_H_26_O_6_ based on HRESIMS ([M + H]^+^*m*/*z* 423.1828, calcd for C_25_H_27_O_6_ 423.1808) and NMR analyses (Table 1 and Figures S10–S18, Supporting Information), implying 13 double-bond equivalents. The ^1^H [δ_H_ 4.93 (d, *J* = 11.8 Hz, H-2), 4.48 (d, *J* = 11.6 Hz, H-3)] and ^13^C [δ_C_ 85.5 (C-2), 74.5 (C-3), and 194.4 (C=O)] NMR data were diagnostic of a dihydroflavonol skeleton, similar to **1**. The presence of two hydroxy groups and a modified geranyl moiety that underwent selective intramolecular 6-*endo*-*trig*-cyclization^24^ to afford a chromene [δ_H_/δ_C_ 6.73 (d, *J* = 2.1 Hz, H-6*′*) /118.3 (C-6*′*); 6.89 (d, *J* = 2.1 Hz, H-2*′*)/116.4 (C-2*′*); 6.40 (d, *J* = 9.9 Hz, H-1″)/123.9 (C-1*″*); and 5.67 (d, *J* = 9.9 Hz, H-2″)/131.2 (C-2*″*)] ring possessing a prenyl chain [δ_H_/δ_C_ 5.12 (m, H-6″)/125.3 (C-6″); 2.15 (m, H-5″)/23.9 (C-5″); 1.79 and 1.66 (m, H-4″)/42.1 (C-4″); 1.66 (s, H-10″)/25.9 (C-10″); and 1.58 (d, H-8″)/17.8 (C-8″)] was apparent from the NMR spectra. The identical ring A of compounds **1** and **2** suggests that **2** was formed from the former through cyclization of the C-5*′* geranyl group with the C-4*′* hydroxy group. The substitution pattern in ring B was confirmed by HMBC correlations, as shown in Table 1. The weak positive CE at 342 nm (for π → n*) and strong negative CE at 290 nm (π → π* transition) in its electronic circular dichroism (ECD) spectrum (Figure 1) and the high negative specific rotation, [α]_D_^24^ −111 (*c* 0.4, CH_3_OH), revealed it to have a 2*R*,3*R* absolute configuration.^22,23^

Whereas the NMR and ECD data provide sufficient information to deduce the absolute configuration of C-2 and C-3, these were not sufficient to determine the chirality of C-3″. The relative stereochemistry of C-3″ and the absolute configuration of C-2 and C-3 could in principle be confirmed using vibrational circular dichroism (VCD) or ECD.^25−28^ Unfortunately, preliminary DFT calculations performed for all possible diastereoisomers showed that the Boltzmann-weighted chiroptical spectra were largely determined by the chirality of the already known chiral centers. The intensities of the calculated Boltzmann-weighted spectra were vastly affected by the molecular flexibility due to the internal rotations of the hydroxy substituents and of the aliphatic side chain containing, among others, the yet to be identified chiral center C-3*″*. To determine the absolute configuration of C-3*″*, we therefore opted for an alternative approach, namely, the estimation of the isotropic shieldings for the ^1^H and ^13^C nuclei of the theoretically possible diastereoisomers, followed by comparison of the theoretically predicted data of the possible diastereomers to those experimentally obtained. 3D structures were derived from DFT calculations, and the relative configuration was assessed using the DP4(+) probabilistic methods.^29−34^ Details of the computational procedure are described in the Experimental Section. The experimental and computational shifts and the DP4(+) results derived by using the toolbox made available^35^ are summarized in Table S1 (Supporting Information). This analysis shows that the absolute configuration of the unknown chiral center C-3*″* of **2** is *R*, with a probability of 100.00%. Based on the above spectroscopic evidence, this new compound (**2**) was identified as (2*R*,3*R,*3*″R*)*-*7,3*′*-dihydroxy-(3″-methyl-3″-(4-methylpent-3-en-1-yl)-4″,5″-pyranoflavanon-3-ol and was given the trivial name gloverinol B.

Gloverinol C (**3**) was isolated as a yellow amorphous solid and was assigned the molecular formula C_25_H_26_O_6_ based on its HRESIMS ([M + H]^+^*m*/*z* 423.1807, calcd for C_25_H_27_O_6_ 423.1808) and NMR data (Table 1 and Figures S19–S27, Supporting Information). Its UV (λ_max_ 235, 271, and 310 nm) and NMR data showed features resembling those of compounds **1** and **2**, indicating that compound **3** is also a dihydroflavonol. Comparison of the NMR data of **3** with those of **2** suggested these compounds to be regioisomers. Thus, the 2-methyl-2-(4-methylpent-3-en-1-yl)pyrano group of **3** is on ring A, between rings C-7 and C-8. The HMBC of δ_H_ 7.66 (H-5) with δ_C_ 194.5 (C-4), 161.6 (C-7), and 159.0 (C-8a) and of δ_H_ 6.50 (H-6) with δ_C_ 161.6 (C-7), 159.0 (C-8a), and 114.2 (C-4a) assigned the AX spin system δ_H_ 7.86 (d, *J* = 8.8 Hz) and 6.94 (d, *J* = 8.8 Hz) to H-5 and H-6 of ring A, respectively. This also allowed the assignment of the pyrocatechol [δ_H_ 7.01 (d, *J* = 2.1 Hz, H-2*′*), 6.89 (dd, *J* = 8.1, 2.1, Hz, H-6*′*), and 6.82 (d, *J* = 8.1 Hz, 5*′*)] moiety to ring B. The weak positive CE at 347 nm (for π → n*) and strong negative CE at 276 nm (π → π*, Figure 1) and the high negative specific rotation, [α]_D_^24^ −89 (*c* 0.2, CH_3_OH), were consistent with **3** being 2*R*,3*R* configured.^22,23^ The absolute configuration at C-3*″* was determined to be *R* with 88.68% probability, following the procedure described above for compound **2**. As compounds **2**, **3**, and **6** (*vide infra*) originate from the same metabolic intermediates, it is plausible that they have the same chirality at the corresponding positions. Although the calculated probability for the configuration of C-3*″* of **3** is slightly less convincing on its own, the comparison of its data with those of **2** and **6** allowed the identification of its absolute configuration with minimal uncertainty. Based on the above spectroscopic data, this new compound (**3**) was identified as the dihydroflavonol (2*R*,3*R,*3*″R*)-3′,4*′*-dihydroxy-(3″-methyl-3″-(4-methylpent-3-en-1-yl)-7,8-geranyl flavanon-3-ol and was given the trivial name gloverinol C.

Gloverinol D (**4**) was obtained as a yellow, amorphous solid. Its HRESIMS ([M + H]^+^*m*/*z* 423.1807, calcd for C_25_H_27_O_6_ 423.1808) along with its NMR data (Table 2 and Figures S28–S36, Supporting Information) were consistent with the molecular formula C_25_H_26_O_6_, revealing 14 degrees of unsaturation. Its UV (λ_max_ 250, 319, 348, and 365 nm) absorption indicated an extended conjugation, which along with the absence of any signal assignable to ring C protons (Table 2) and the ^13^C NMR signals at δ_C_ 138.3 (C-2), 147.7 (C-3), and 174.8 (C-4) were consistent with a flavonol skeleton.^36^ The ^13^C NMR spectrum exhibited 25 carbon resonances, of which 15 signals were ascribed to the flavon-4-ol core and 10 signals to a geranyl substituent. The NMR along with the MS data were consistent with the presence of a geranyl and three hydroxy substituents.^36^ Two *ortho*-coupled protons, ^3^*J*_H-5/H-6_ = 8.8 Hz, resonating at δ_H_ 7.86 and 6.94, were assigned to H-5 and H-6 of ring A, respectively. Hydroxy substitution at C-7 (δ_C_ 161.3) and geranyl substitution at C-8 (δ_C_ 116.8) of ring A were unambiguously established based on the HMBC correlations of δ_H_ 3.68 (H-1″) with δ_C_ C-7 (161.3), C-8 (116.8), C-8a (156.3), C-2″ (123.3), and C-3″ (136.7). In ring B, an AMX spin system was observed at δ_H_ 7.84 (d, *J* = 2.2 Hz, H-2*′*), 7.66 (d, *J* = 8.5, 2.2 Hz, H-6*′*), and 6.89 (d, *J* = 8.5 Hz, H-5*′*), which was consistent with the placement of the remaining two hydroxy groups at C-3*′* (δ_C_ 146.3) and C-4*′* (δ_C_ 148.7), respectively. The substitution pattern of this ring was confirmed by the HMBC correlations of δ_H_ 7.84 (H-2*′*) with C-2 (δ_C_ 147.7), C-3*′* (δ_C_ 146.3), C-4*′* (δ_C_ 148.7), and C-6*′* (δ_C_ 121.5), δ_H_ 6.89 (H-5′) with C-1*′* (δ_C_ 124.7), C-3*′* (δ_C_ 146.3), and C-4*′* (δ_C_ 148.7), and δ_H_ 7.66 (H-6*′*) with C-2 (δ_C_ 147.7), C-2*′* (δ_C_ 116.4), C-4*′* (δ_C_ 148.7), and C-5*′* (δ_C_ 116.5). Based on the above spectroscopic data, this new compound (**4**) was identified as 7,3*′*,4*′*-trihydroxy-8-geranylflavan-3-ol and was given the trivial name gloverinol D.

**Table 2:** NMR Spectroscopic Data (^1^H 500 MHz, ^13^C 125 MHz, MeOH-*d*_4_) for Gloverinol D (**4**)

position	δ_C_, type	δ_H_ m (*J* in Hz)	HMBC
2	147.7, C		
3	138.3, C		
4	174.8, C		
4a	115.6, C		
5	124.4, CH	7.86 d (8.8)	C-4, C-7, C-8, C-8a
6	115.2, CH	6.94 d (8.8)	C-4a, C-7, C-8
7	161.3, C		
8	116.8, C		
8a	156.3, C		
1*′*	124.7, C		
2*′*	116.4, CH	7.84 d (2.2)	C-3*′*, C-4*′*, C-6*′*
3*′*	146.3, C		
4*′*	148.7, C		
5*′*	116.3, CH	6.89 d (8.5)	C-1*′*, C-3*′*, C-4*′*
6*′*	121.5, CH	7.66 dd (8.5, 2.2)	C-2, C-2*′*, C-4*′*, C-5*′*
1″	23.0, CH_2_	3.68 d (6.9)	C-7, C-8, C-8a, C-2″, C-3″
2″	123.3, CH	5.28 ddq (6.9, 6.9, 1.3)	C-8, C-1″, C-4″, C-9″
3″	136.7, C		
4″	40.7, CH_2_	1.99 m	C-2″, C-3″, C-5″, C-6″, C-9″
5″	27.5, CH_2_	2.04 m	C-3″, C-4″, C-6″, C-7″
6″	125.3, CH	4.98 ddqq (6.8,6.8,1.4, 1.4)	C-8″, C-10″
7″	132.1, C		
8″	25.7, CH_3_	1.51 d (1.4)	C-4″, C-6″, C-7″, C-10″
9″	16.7, CH_3_	1.82 d (1.3)	C-2″, C-3″, C-4″
10″	17.6, CH_3_	1.46 d (1.4)	C-4″, C-6″, C-7″, C-8″

**Figure 2:**
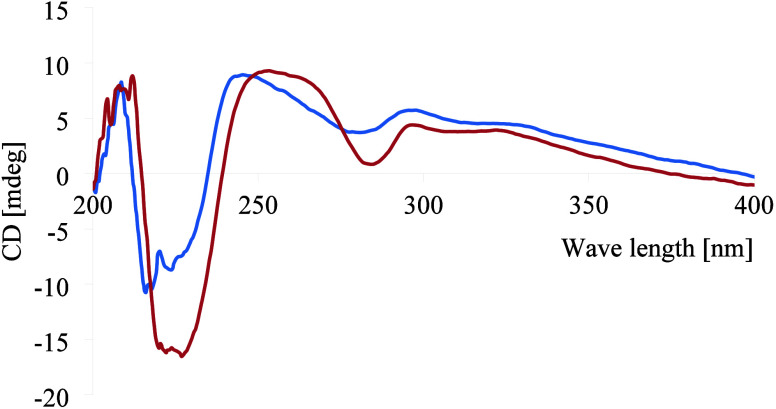
ECD spectra (MeOH) of gloveriflavans A (**5**, blue) and B (**6**, red).

Gloveriflavan A (**5**) was isolated as a yellow amorphous solid and was assigned the molecular formula C_26_H_33_O_5_ based on its HRESIMS ([M + H]^+^*m*/*z* 425.2312, calcd 425.2328) and NMR data (Table 3, Figures S37–S45, Supporting Information). Its ^1^H NMR spectrum showed two sets of diastereotopic protons at δ_H_ 4.30 and 3.95 (CH_2_-2, δ_C_ 71.2) and δ_H_ 2.95 and 2.80 (CH_2_-4, δ_C_ 32.0) and an oxymethine proton at δ_H_ 3.45 (H-3, δ_C_ 33.4) that were characteristic for the ring C of an isoflavan.^37^ The NMR and MS data further suggested the presence of a methoxy, a geranyl, and three hydroxy substituents. The *ortho*-coupled aromatic protons, *J* = 8.2 Hz, at δ_H_ 6.69 and 6.32 were assigned to H-5 and H-6, respectively. The C-7 oxygenation (δ_C_ 154.9) was derived from biogenetic considerations. The position of the geranyl group at C-8 was determined based on the HMBC of H-1″ (δ_H_ 3.28) with C-7 (δ_C_ 154.9) and C-8a (δ_C_ 154.1), while H-6 (δ_H_ 6.32) correlated with C-7 (δ_C_ 154.9) and C-8 (δ_C_ 117.9). Ring B was determined to be trisubstituted with two hydroxy and a methoxy group at C-2*′* (δ_C_ 144.9), C-3*′* (δ_C_ 134.7), and C-4*′* (δ_C_ 148.3), respectively. The chemical shift value of the methoxy group (δ_C_ 56.5) is “normal”, and hence it is placed at C-4*′* rather than at C-2*′* or C-3*′*, as a methoxy at the latter two positions would be expected to appear above 59 ppm, due to di-*ortho*-substitution.^38^ The placement of the methoxy group at C-4*′* was supported by the HMBC correlation of the methoxy protons (δ_H_ = 3.81) to C-4*′* (δ_C_ = 148.3). The *ortho*-coupled, *J* = 8.6 Hz, aromatic protons of this ring at δ_H_ 6.43 and 6.55 Hz were assigned to H-5*′* and H-6*′*, respectively. Comparison of the NMR data of **5** with those of nitiducol^37^ indicated that they have an identical ring B. The ECD spectrum (Figure 2), which showed a positive CE at 242 nm and a negative CE at 227 nm, is consistent with a 3*R* absolute configuration.^21^ Based on the above spectroscopic evidence, this new compound, gloveriflavan A (**5**), was characterized as 7,2*′*,3*′*-trihydroxy-4*′*-methoxy-8-geranylisoflavan.

**Table 3:** NMR Spectroscopic Data (^1^H NMR 500 MHz and ^13^C NMR 125 MHz, MeOH-*d*_4_) for Gloveriflavans A (**5**) and B (**6**)

	**5**			**6**		
position	δ_C_, type	δ_H_ m (*J* in Hz)	HMBC	δ_C_, type	δ_H_ m (*J* in Hz)	HMBC
2α	71.2, CH_2_	4.30 ddd (10.2, 3.5, 2.0)	C-3, C-4, C-8a, C-1*′*	71.0, CH_2_	4.23 m	C-3, C-4, C-8a, C-1*′*
2β		3.95 dd (10.2, 10.1)	C-3, C-4, C-8a, C-1*′*		3.99 m	C-3, C-4, C-8a, C-1*′*
3	33.4, CH	3.45 dddd (11.0, 10.1, 5.3, 3.5)	C-2, C-4, C-4a, C-1*′*, C-2*′*, C-6*′*	33.8, CH	3.46 m	C-2, C-4, C-4a, C-1*′*, C-2*′*, C-6*′*
4α	32.0, CH_2_	2.95 ddd (15.5, 11.0, 1.1)	C-2, C-3, C-4a, C-5, C-6, C-8, C-8a, C-1*′*	31.4, CH_2_	2.93 ddd (15.6, 10.2)	C-2, C-3, C-4a, C-5, C-6, C-7, C-8, C-8a, C-1*′*
4β		2.80 ddd (15.5, 5.3, 2.0)	C-2, C-3, C-4a, C-5, C-6, C-8a, C-1*′*		2.83 dddd (15.6, 5.0, 2.2, 2.0)	C-2, C-3, C-4a, C-5, C-6, C-8a, C-1*′*
4a	114.8, C			114.8, C		
5	127.8, CH	6.69 d (8.2)	C-4, C-6, C-7, C-8, C-8a, C-6*′*	131.2, CH	6.88 d (8.3)	C-4, C-4a, C-6, C-7, C-8, C-8a
6	108.5, CH	6.32 d (8.2)	C-4a, C-7, C-8, C-8a, C-1″	109.0, CH	6.32 dd (8.3, 2.2)	C-4a, C-7, C-8
7	154.9, C			157.6, C		
8	116.7, C			103.8, CH	6.22 d (2.2)	C-4, C-4a, C-6, C-7, C-8a
8a	154.1, C			156.4, C		
1*′*	122.7, C			123.5, C		
2*′*	144.9, C			144.6, C		
3*′*	135.0, C			139.7, C		
4*′*	148.3, C			148.2, C		
5*′*	104.0, CH	6.43 d (8.6)	C-3, C-1*′*, C-2*′*, C-3*′*, C-4*′*, C-6*′*	131.6, C		
6*′*	117.9, CH	6.55 d (8.6)	C-2, C-3, C-1*′*, C-2*′*, C-3′, C-4*′*, C-5*′*	117.7, CH	6.48 s	C-2, C-3, C-1*′*, C-2*′*, C-4*′*, C-5*′*, C-3″
1″	23.1, CH_2_	3.28 d (7.2)	C-7, C-8, C-8a, C-2″, C-3″, C-4″, C-5″, C-9″	110.6, CH_2_	4.96 dd (10.8, 1.5)	C-5*′*, C-2″, C-3″, C-9″
					4.89 dd (17.6, 1.5)	C-5*′*, C-2″, C-3″, C-9″
2″	124.8, CH	5.21 m	C-8, C-1″, C-4″, C-5″, C-9″	149.8, CH	6.16 ddd (17.6, 10.8)	C-5*′*, C-3″, C-4″, C-9″
3″	134.7, C			44.9, C		
4″	41.0, CH_2_	1.94 dd (8.8)	C-2″, C-3″, C-5″, C-6″, C-9″	41.2, CH_2_	1.96 m	C-5*′*, C-2″, C-3″, C-5″, C-6″, C-9″
					1.65 m	C-5*′*, C-2″, C-3″, C-5″, C-9″
5″	27.8, CH_2_	2.05 m	C-3″, C-4″, C-6″, C-7″, C-8″, C-9″, C-10″	24.7, CH_2_	1.78 m	C-3″, C-4″, C-6″, C-7″,
					1.65 m	C-3″, C-4″, C-6″, C-7″, C-10″
6″	125.6, CH	5.07 m	C-4″, C-5″, C-8″, C-10″	126.2, CH	5.04 m	C-4″, C-5″, C-8″, C-10″
7″	132.0, C			131.7 or 131.6, C		
8″	25.9, CH_3_	1.62 d (1.4)	C-4″, C-5″, C-6″, C-7″, C-10″	25.9, CH_3_	1.63 s	C-6″, C-7″, C-10″
9″	16.3, CH_3_	1.75 d (1.4)	C-8, C-1″, C-2″, C-3″, C-4″, C-5″	25.9, CH_3_	1.34 s	C-5*′*, C-6′, C-1″, C-2″, C-3″, C-4″
10″	17.7, CH_3_	1.56 d (1.4)	C-4″, C-5″, C-6″, C-7″, C-8″	17.7, CH_3_	1.49 s	C-4″, C-6″, C-7″, C-8″
4*′*-OCH_3_	56.5, CH_3_	3.81 s	C-4*′*, C-5*′*	60.2, CH_3_	3.74 s	C-4*′*

Gloveriflavan B (**6**) was obtained as a yellow amorphous solid. Its molecular formula was determined to be C_26_H_32_O_5_ based on HRESIMS ([M + H]^+^*m*/*z* 425.2312, calcd for C_26_H_33_O_5_ 425.2328) and NMR data (Table 3 and Figures S46–S54, Supporting Information). Similar to the NMR spectra of **5**, those of compound **6** displayed characteristic features for an isoflavan backbone and for three hydroxy, a methoxy, and a modified geranyl substituent. Ring A of **6** indicated the presence of an AMX spin-system [δ_H_ 6.88 (d, *J* = 8.3 Hz, H-5), 6.32 (dd, *J* = 8.3, 2.2, Hz, H-6), and 6.22 (d, *J* = 2.2 Hz, H-8)], and the biogenetically expected oxygenation at C-7 required the geranyl, the two hydroxy, and the methoxy groups to be located in ring B. The geranyl group has undergone a Claisen rearrangement from a 3*′*-*O*-geranylated precursor,^24,39^ placing the modified geranyl group, a “reverse geranyl”,^40,41^ at C-5*′*. The assignment of the two *gem* olefinic protons as *pro-Z* (δ_H_ 4.96) and *pro-E* (δ_H_ 4.89) was based on their coupling constant, ^3^*J*_H-1″,H-2″_ = 10.8 Hz and ^3^*J*_H-1″,H-2″_ = 17.6 Hz, respectively. The placement of this group at C-5′ was confirmed from the HMBC correlations of H-6*′* (δ_H_ 6.48) to C-5*′* (δ_C_ 131.7), C-4*′* (δ_C_ 148.2), and C-3″ (δ_C_ 44.9). Finally, the methoxy group (δ_H_ 3.74; δ_C_ 60.2) was placed at C-5″ based on the HMBC correlations of its protons as well as of H-6*′* (δ_H_ 6.48) to C-4*′* (δ_C_ 148.2). The positive CE at 258 nm and a negative CE at 230 nm, in the ECD spectrum (Figure 2), indicated **6** to be 3*R* configured.^21^ The absolute configuration of C-3″ was determined to be *R* with a probability of 99.99%, following the procedure described above for compounds **2** and **3**. Based on the above spectroscopic evidence, this new compound (**6**) was identified as (3*R,3″R*)*-*7,2*′*,3*′*-trihydroxy-4*′*-methoxy-5*′*-geranylisoflavan and was given the trivial name gloveriflavan B.

The isolated compounds were tested for *in vitro* antimicrobial activity against the Gram-positive bacterium *Staphylococcus aureus*, the Gram-negative bacterium *Escherichia coli*, and the fungus *Candida albicans* (Table 4). *S. aureus* was the most sensitive to compounds **2**, **6**, and **10** with MIC values of 9.2, 18.4, and 14.2 μM, respectively. Compounds **1**, **3**, **5**, **7**, and **8** showed lower activity (MIC = 36.7, 36.9, 36.7, 40 μM, respectively) against *S. aureus*. When tested against the opportunistic pathogenic yeast *C. albicans*, compounds **7**, **8**, and **10** showed equipotent activities (MIC = 36.9, 40.0, 56.9 μM). Only compound **8** (MIC = 64.0 μM) was active against *E. coli*. Some flavonoids isolated from the genus *Dalbergia*, such as sativanone, liquiritigenin, and sulfuretin from *D. odorifera*, have showed antibacterial activity.^42^ This is in line with the traditional medicinal use of some members of this genus for the treatment of microbial infections, such as cough and skin diseases.^42^

**Table 4:** Antimicrobial Activity of the Isolated Constituents of *D. gloveri’*s Root Bark

	MIC in μM		
sample	*S. aureus*	*E. coli*	*C. albicans*
**1**	36.7	294.5	73.7
**2**	9.2	295.9	74.1
**3**	36.9	>1000	74.1
**4**	>1000	>1000	>1000
**5**	36.7	294.4	73.7
**6**	18.4	>1000	73.7
**7**	36.9	295.8	36.9
**8**	40.0	64.0	40.0
**9**	487.8–975.6	>1000	>1000
**10**	14.2	>1000	56.9
**11**	293.1	>1000	146.6
**12**	612.1	>1000	>1000
**13**	250.6–501.3	>1000	250.6
Octenidine	0.5	0.5–1	0.5

In conclusion, three new flavononols (**1**–**3**), a new flavonol (**4**), and two new flavans (**5**, **6**) along with seven known compounds were isolated from the root bark of *D. gloveri*. Most of the isolated compounds are geranylated, which is in agreement with previous observations for the root extracts of this genus^43,44^ and is thus of chemotaxonomic significance. In line with the traditional medicinal use of this genus, some of the isolated compounds showed moderate to good antimicrobial activities against *S. aureus*, *E. coli*, and *C. albicans*. Compounds **2**, **6**, and **10** were the most active (MIC 9.0–184 μM) against *S. aureus*, and compound **8** was active against *E. coli* (MIC 64.0 μM). The reported structures may initiate synthetic efforts aiming at the development of new antibacterial lead compounds, which is of high significance due to the rapid resistance development of bacteria against the existing antimicrobials.

## Experimental Section

### General Experimental Procedures

Optical rotations were measured on a PerkinElmer 341-LC instrument, whereas ECD experiments were performed on a Jasco J-715 spectropolarimeter. UV spectra were recorded on a Specord S600 (Analytik Jena AG) spectrophotometer. NMR spectra were acquired by using a Bruker Avance Neo 500 MHz spectrometer equipped with a 5 mm cryogenic TXO probe. The spectra were processed using MestReNova 14.1 software and were referenced to the residual solvent peak. LC-ESIMS data were acquired on a Waters Micromass ZQ Multimode ionization electrospray ionization (ESI) instrument connected to an Agilent 1100 series gradient pump system and a C_8_ column (Gemini), using Milli-Q H_2_O/CH_3_CN (5:95 to 95:5, with 0.1% HCO_2_H over 4 min). High-resolution accurate mass measurements were performed by ESIMS using an LTQ-Velos Pro Orbitrap mass analyzer (Thermo Fisher Scientific, Waltham, MA, USA) equipped with an Agilent 1100 autosampler (Agilent, Santa Clara, CA, USA) with a bioZen Peptide XB-C18 column (100 mm × 2.1 mm, 1.7 μm). The gradient used was from 5% to 95% CH_3_CN (with 0.01% formic acid) in H_2_O (with 0.01% formic acid) at 0.8 mL/min. MS was scanned from 100 to 2500 Da at 1 scan/s. Each mass spectrum was obtained in positive-ion mode, and the obtained data were processed using MassLynx V4.1 software. TLC analyses were carried out on Merck precoated silica gel 60 F_254_ plates. Preparative TLCs were performed on glass plates of 20 × 20 cm dimension, precoated with silica gel 60 F_254_ having 0.25 to 1 mm thickness. Column chromatography was run on silica gel (40–63 μm mesh). Gel filtration was performed on Sephadex LH-20.

### Plant Material

The root bark of *D. gloveri* Q. Luke. ined. was collected in September 2020 from Gongoni forest, Kwale County, Kenya. The plant material was authenticated by Patrick Chalo Mutiso of the Herbarium, Department of Biology, University of Nairobi, Kenya, where a voucher specimen (UON_DMC2020_002) was deposited.

### Extraction and Isolation

The dried and ground root bark of *D. gloveri* (800 g) was extracted with CH_2_Cl_2_/MeOH (1:1) (3 × 1.5 L × 72 h) by cold maceration. The extract was filtered, and the supernatant was concentrated under reduced pressure to obtain a dark brown crude extract (50 g). A portion of the crude extract (30 g) was subjected to column chromatography on silica gel (400 g) using *iso*-hexane containing increasing amounts of EtOAc. A total of 120 fractions, each ca. 250 mL, were collected. The first 20 fractions eluted with 0–4% EtOAc in *iso-*hexane (a mixture of hexanes) contained mainly fatty acids and were not followed further. Fractions 21–30 eluted with 8% EtOAc in *iso*-hexane were combined and crystallized from a CH_2_Cl_2_/MeOH mixture, to give oleanolic acid acetate (**13**, 20 mg). Fractions 31–45 eluted with 12–14% EtOAc in *iso-*hexane were combined and subjected to column chromatography on Sephadex LH-20 (CH_2_Cl_2_/MeOH, 1:1) to give nitidulin (**7**, 10 mg) and a mixture of two compounds. The mixture was further purified by silica gel column chromatography (CH_2_Cl_2_/*iso*-hexane, 1:1) affording gloveriflavan A (**5**, 5 mg) and nitidulin (**7**, 1 mg). Fractions 46–59 eluted with 16–18% EtOAc in *iso-*hexane were combined and purified on Sephadex LH-20 (CH_2_Cl_2_/MeOH, 1:1) yielding gloveriflavan B (**6**, 3 mg), lespeol (**8**, 5 mg), and a mixture of **6** and **10**. Preparative TLC (*iso-*hexane/EtOAc, 4:1) was further carried out to furnish compound **6** (0.9 mg) and 1-(2,4-dihydroxyphenyl)-3-hydroxy-3-(4-hydroxyphenyl)-1-propanone (**10**, 6 mg). Using silica gel column chromatography, fractions 60–89 eluted with 24% EtOAc in *iso-*hexane were separated on silica gel (eluent: *iso-*hexane/CH_2_Cl_2_, 1:1), affording gloverinol B (**2**, 7 mg) together with gloverinol A (**1**, 5 mg) and mixture of gloverinol C (**3**), gloverinol D (**4**), and isoliquiritigenin (**9**). This mixture was further subjected to Sephadex LH-20 (CH_2_Cl_2_/MeOH, 1:1), producing gloverinol C (**3**, 3 mg), gloverinol D (**4**, 2 mg), and isoliquiritigenin (**9**, 6 mg). Sephadex LH-20 (CH_2_Cl_2_/MeOH, 1:1) on fractions 88–100 eluting with 30% EtOAc in *iso*-hexane yielded (2*R*)-1,2-dihydro-2-[1-(hydroxymethyl)ethenyl]-8,9-dimethoxy[1]benzopyrano[3,4-*b*]furo[2,3-*h*][1]benzopyran-6(12*H*)-one (**12**, 8 mg) and dalbinol (**11**, 10 mg).

#### Gloverinol A (**1**)

Yellow solid; [α]_D_^24^ −114 (*c* 0.2, CH_3_OH); UV (MeOH) λ_max_ 276 nm (4.12), 307 nm (3.42); ECD (*c* 0.03, CH_3_OH) λ_max_ (*Δε*) 305 (−20.7), 253 (6.3); ^1^H and ^13^C NMR (Table 1); HRESIMS [M + H]^+^*m*/*z* 425.1974 (calcd for C_25_H_29_O_6_, 425.1964).

#### Gloverinol B (**2**)

Yellow amorphous solid; [α]_D_^24^ −111 (*c* 0.41, CH_3_OH); UV (MeOH) λ_max_ 231 nm (4.03), 271 nm (3.32), 309 nm; ECD (*c* 0.06, CH_3_OH) λ_max_ (Δε) 340 (4.1), 314 (−14.1); 293 (−33.8); ^1^H and ^13^C NMR (Table 1); HRESIMS [M + H]^+^*m*/*z* 423.1828 (calcd for C_25_H_27_O_6_, 423.1808).

#### Gloverinol C (**3**)

Yellow amorphous solid; [α]_D_^24^ – 89 (*c* 0.2, CH_3_OH); UV (MeOH) λ_max_ 235 nm (4.21), 271 nm (3.65), 310 nm (3.32); ECD (c 0.02, CH_3_OH) λ_max_ (Δε) 350 (4.4), 318 (−12.2), 294 (−10.7); 273 (−23.3); ^1^H and ^13^C NMR (Table 2); HRESIMS [M + H]^+^*m*/*z* 423.1807 (calcd for C_25_H_27_O_6_, 423.1808).

#### Gloverinol D (**4**)

Yellow amorphous solid; UV (MeOH) λ_max_ 250 nm (3.99), 319 nm (3.46), 348 nm (3.23), 365 nm; ^1^H and ^13^C NMR (Table 2); HRESIMS [M + H]^+^*m*/*z* 423.1807 (calcd for C_25_H_27_O_5_, 423.1808).

#### Gloveriflavan A (**5**)

Yellow amorphous solid; [α]_D_^24^ −131 (*c* 0.3, CH_3_OH); UV (MeOH) λ_max_ 273 nm (4.22), 297 nm (3.56); ECD (c 0.04, CH_3_OH) λ_max_ (Δε) 249 (8.6), 299 (5.6); ^1^H and ^13^C NMR (Table 3); HRESIMS [M + H]^+^*m*/*z* 425.2312 (calcd for C_26_H_33_O_5_, 425.2328).

#### Gloveriflavan B (**6**)

Yellow amorphous solid; [α]_D_^24^ −121 (*c* 0.6, CH_3_OH); UV (MeOH) λ_max_ 275 nm (4.32), 296 nm (3.87); ECD (c 0.03, CH_3_OH) λ_max_ (Δε) 254 (9.1), 299 (4.2); ^1^H and ^13^C NMR (Table 3); HRESIMS [M + H]^+^*m*/*z* 425.2312 (calcd for C_26_H_33_O_5_, 425.2328).

### Antimicrobial Activity

The antimicrobial activity was determined through microdilution method. Three pathogenic microorganisms including Gram-positive (*Staphylococcus aureus*), Gram-negative (*Escherichia coli*), and fungi (*Candida albicans*) were investigated. The minimal inhibitory concentrations (MIC) of compounds were determined by the microdilution method using 96-well plates (Nest Scientific Biotechnology, China). The studies were conducted according to the methodology described in our previous publications.^45,46^ Briefly, 90 μL of tryptic soy broth TSB (Graso Biotech, Poland) and 10 μL of microbial suspension were placed into each well to a final inoculum concentration of 10^6^ CFU/mL. A suspension was performed by using McFarland standards. Serial dilutions of compounds were performed to obtain concentrations from 1000 to 1.95 μg/mL. 10% dimethyl sulfoxide (DMSO) was added as negative control. The plates were incubated at 35 °C for 24 h. MIC was determined by visual analysis. The compounds tested had >95% purity (see NMR spectra in the Supporting Information).

### Computational Details

Conformational analysis was performed using PCMODEL version 10.0, using the MMFF94 force field and by applying 10 and 8 kcal mol^–1^ energy windows for two consecutive conformational search cycles. Subsequently, geometry optimization, frequency and shielding tensor quantum mechanical calculations were performed using Gaussian16 RevC.^47^ Boltzmann populations was estimated using the sum of electronic and thermal free energies at 298.15 K obtained at the B3LYP/6-31G* level. Isotropic shielding constants for NMR predictions were derived at the mPW1PW91/6-31++G(d,p) level using the B3LYP/6-31G* equilibrium geometries. For all calculations, corrections for the solvent (methanol) were introduced by using the SCRF polarized continuum model. DP4(+) probabilities were obtained by combining the experimental and calculated chemical shifts for all ^1^H and ^13^C atoms involved, and by using the DP4+ toolbox made available by A.M. Sarroti and co-workers.^35^

## Data Availability

The original FIDs and MestreNova files for all compounds, NMReDATA^48,49^ files and CSEARCH^50^ results for the isolated compounds, HRMS, ECD and UV spectra, and details of the DFT computations for the new compounds **1**–**6** are freely available on Zenodo (DOI: 10.5281/zenodo.11075514).

## Supporting Information Available

NMR, MS, and optical spectroscopy data for the isolated compounds and antibacterial activity (PDF)
