# Supplementary material for: Antimicrobial Dihydroflavonols and Isoflavans Isolated from the Root Bark of Dalbergia gloveri
Source: J Nat Prod. 2024 Sep 10;87(9):2263–71. doi: 10.1021/acs.jnatprod.4c00690 (PMC11443528; doi:10.1021/acs.jnatprod.4c00690)
Supplement: Supplementary file 1 — np4c00690_si_001.pdf [file np4c00690_si_001.pdf]

SUPPORTING INFORMATION

Antimicrobial Dihydroflavonols and Isoflavans Isolated from the Root Bark of *Dalbergia gloveri*

Ivan Kiganda, Lianne H. E. Wieske, Vaderament-Alexe Nchiozem-Ngnitedem, Duncan Chalo Mutiso, Daniel Umereweneza, Albert Ndakala, Wouter Herrebout, Ruisheng Xiong, Tomasz M. Karpiński, Abiy Yenesew, and Mate Erdelyi

Table of Contents

Natural Products Isolated from the root bark of *Dalbergia gloveri* Q. Luke. Ined............................. S3

Spectroscopic Data of Gloverinol A (**1**)... S4

Figure S1. ^{1}H NMR (500 MHz, MeOH-d_4, 25 ºC) spectrum of gloverinol A (**1**) S4

Figure S2. ^{13}C NMR (125 MHz, MeOH-d_4, 25 ºC) spectrum of gloverinol A (**1**) S4

Figure S3. COSY (500 MHz, MeOH-d_4, 25 ºC) spectrum of gloverinol A (**1**).............................. S5

Figure S4. HSQC (500/125 MHz, MeOH-d_4, 25 ºC) spectrum of gloverinol A (**1**)....................... S5

Figure S5. HMBC (500/125 MHz, MeOH-d_4, 25 ºC) spectrum of gloverinol A (**1**)...................... S6

Figure S6. NOESY (500 MHz, MeOH-d_4, 25 ºC) spectrum of gloverinol A (**1**) S6

Figure S7. TOCSY (500 MHz, MeOH-d_4, 25 ºC) spectrum of gloverinol A (**1**)............................ S7

Figure S8. HRESIMS spectrum of gloverinol A (**1**).. S7

Figure S9. UV spectrum of gloverinol A (**1**).. S8

Spectroscopic Data of gloverinonol B (**2**)... S8

Figure S11. ^{1}H NMR (500 MHz, MeOH-d_4, 25 ºC) spectrum of gloverinol B (**2**)......................... S8

Figure S12. ^{13}C NMR (125 MHz, MeOH-d_4, 25 ºC) spectrum of gloverinol B (**2**)........................ S9

Figure S13. COSY (500 MHz, MeOH-d_4, 25 ºC) spectrum of gloverinol B (**2**) S9

Figure S14. HSQC (500/125 MHz, MeOH-d_4, 25 ºC) spectrum of gloverinol B (**2**) S10

Figure S15: HMBC (500/125 MHz, MeOH-d_4, 25 ºC) spectrum of gloverinol B (**2**) S10

Figure S16. NOESY (500 MHz, MeOH-d_4, 25 ºC) spectrum of gloverinol B (**2**)..........................S11

Figure S17. TOCSY (500 MHz, MeOH-d_4, 25 ºC) spectrum of gloverinol B (**1**)..........................S11

Figure S18. HRESIMS spectrum of gloverinol B (**2**) S12

Figure S19. UV spectrum of gloverinol B (**2**) S12

Spectroscopic Data of gloverinol C (**3**)..S13

Figure S20. ^{1}H NMR (500 MHz, MeOH-d_4, 25 ºC) spectrum of gloverinol C (**3**).........................S13

Figure S21. ^{13}C NMR (125 MHz, MeOH-$d4$, 25 ºC) spectrum of gloverinol C (**3**).......................S13

Figure S22. COSY (500 MHz, MeOH-d_4, 25 ºC) spectrum of gloverinol C (**3**) S14

Figure S23. HSQC (500/125 MHz, MeOH-d_4, 25 ºC) spectrum of gloverinol C (**3**) S14
Figure S24. HMBC (500/125 MHz, MeOH-d_4, 25 ºC) of gloverinol C (**3**) S15
Figure S25. NOESY (500 MHz, MeOH-d_4, 25 ºC) spectrum of gloverinol C (**3**).......................... S15
Figure S26. TOCSY (500 MHz, MeOH-d_4, 25 ºC) spectrum of gloverinol B (**1**).......................... S16
Figure S26. HRESIMS spectrum of gloverinol C (**3**) S16
Figure S27. UV spectrum of gloverinol C (**3**) S17
Spectroscopic Data of gloverinol D (**4**) S17
Figure S28. ^{1}H NMR (500 MHz, MeOH-d_4, 25 ºC) spectrum of gloverinol D (**4**) S17
Figure S30. COSY (500 MHz, MeOH-d_4, 25 ºC) spectrum of gloverinol D (**4**)........................... S18
Figure S32. HMBC (500/125 MHz, MeOH-d_4, 25 ºC) spectrum of gloverinol D (**4**) S19
Figure S33. NOESY (500 MHz, MeOH-d_4, 25 ºC) spectrum of gloverinol D (**4**) S20
Figure S34. TOCSY (500 MHz, MeOH-d_4, 25 ºC) spectrum of gloverinol D (**4**).......................... S20
Figure S35. HRESIMS spectrum of gloverinol D (**4**) S21
Figure S36. UV spectrum of gloverinol D (**4**) S21
Spectroscopic Data of gloveriflavan A (**5**) S22
Figure S37. ^{1}H NMR (500 MHz, MeOH-d_4, 25 ºC) spectrum of gloveriflavan A (**5**) S22
Figure S38. ^{13}C NMR (125 MHz, MeOH-d_4, 25 ºC) spectrum of gloveriflavan A (**5**) S22
Figure S39. COSY (500 MHz, MeOH-d_4, 25 ºC) spectrum of gloveriflavan A (**5**) S23
Figure S40. HSQC (500/125 MHz, MeOH-d_4, 25 ºC) spectrum of gloveriflavan A (**5**) S23
Figure S42. NOESY (500 MHz, MeOH-d_4, 25 ºC) spectrum of gloveriflavan A (**5**)..................... S24
Figure S43. TOCSY (500 MHz, MeOH-d_4, 25 ºC) spectrum of gloveriflavan A (**5**)..................... S25
Figure S44. HRESIMS spectrum of gloveriflavan A (**5**) S25
Figure S45. UV spectrum of gloveriflavan A (**5**) S26
Spectroscopic Data of gloveriflavan B (**6**) S26
Figure S46. ^{1}H NMR (500 MHz, MeOH-d_4, 25 ºC) spectrum of gloveriflavan B (**6**) S26
Figure S47. ^{13}C NMR (125 MHz, MeOH-d_4, 25 ºC) spectrum of gloveriflavan B (**6**) S27
Figure S48. COSY (500 MHz, MeOH-d_4, 25 ºC) spectrum of gloveriflavan B (**6**) S27
Figure S49. HSQC (500/125 MHz, MeOH-d_4, 25 ºC) spectrum of gloveriflavan B (**6**)................. S28
Figure S50. HMBC (500/125 MHz, MeOH-d_4, 25 ºC) spectrum of gloveriflavan B (**6**)................ S28
Figure S51. NOESY (500 MHz, MeOH-d_4, 25 ºC) spectrum of gloveriflavan B (**6**)..................... S29
Figure S52. TOCSY (500 MHz, MeOH-d_4, 25 ºC) spectrum of gloveriflavan B (6) S29
Figure S53. HRESIMS spectrum of gloveriflavan B (**6**) S30
Figure S54. UVspectrum of gloveriflavan B (**6**) S30
Spectroscopic Data of nitidulin (**7**) S31
Spectroscopic Data of 2',4,4',β-tetrahydroxydihydrochalcone (**8**).. S34
Spectroscopic Data of isoliquiritigenin (**9**) S38
Spectroscopic Data of 1-(2,4-dihydroxyphenyl)-3-hydroxy-3-(4-hydroxyphenyl)-1-propanone (**10**) S41

Spectroscopic Data of dalbinol (**11**) S45

Spectroscopic Data of (2*R*)-1,2-dihydro-2-[1-(hydroxymethyl)ethenyl]-8,9-dimethoxy[1]benzopyrano[3,4-*b*]furo[2,3-*h*][1]benzopyran-6(12*H*)-one (**12**) S48

Spectroscopic Data of oleanolic acid acetate (**13**) S52

ECD calculation..S55

The original FIDs and MestreNova files for all compounds, NMReDATA files and CSEARCH results for the isolated compounds, HRMS, ECD and UV spectra, and details of the DFT computations for the new compounds **1**-**6** are freely available on Zenodo (DOI:10.5281/zenodo.11075514).

Natural Products Isolated from the root bark of *Dalbergia gloveri* Q. Luke. Ined

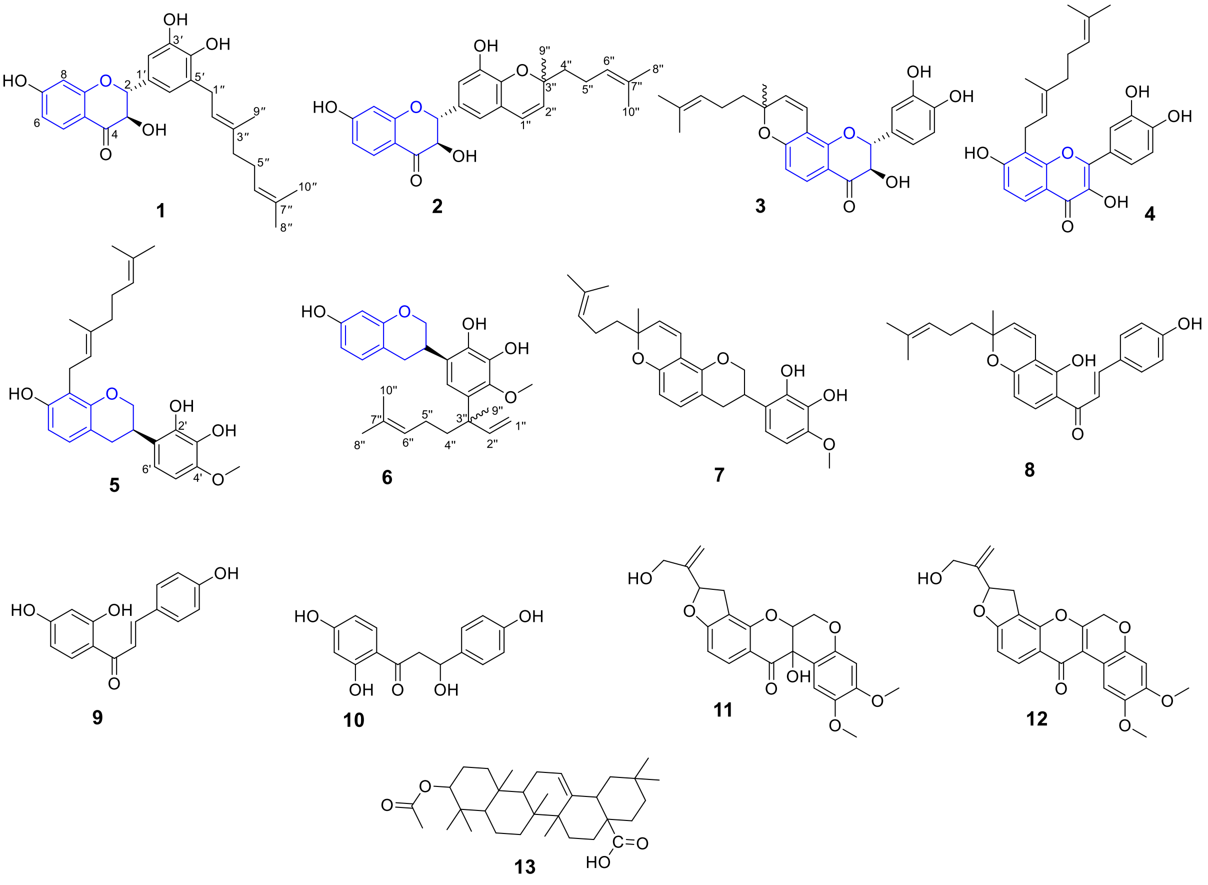

Spectroscopic Data of Gloverinol A (1)

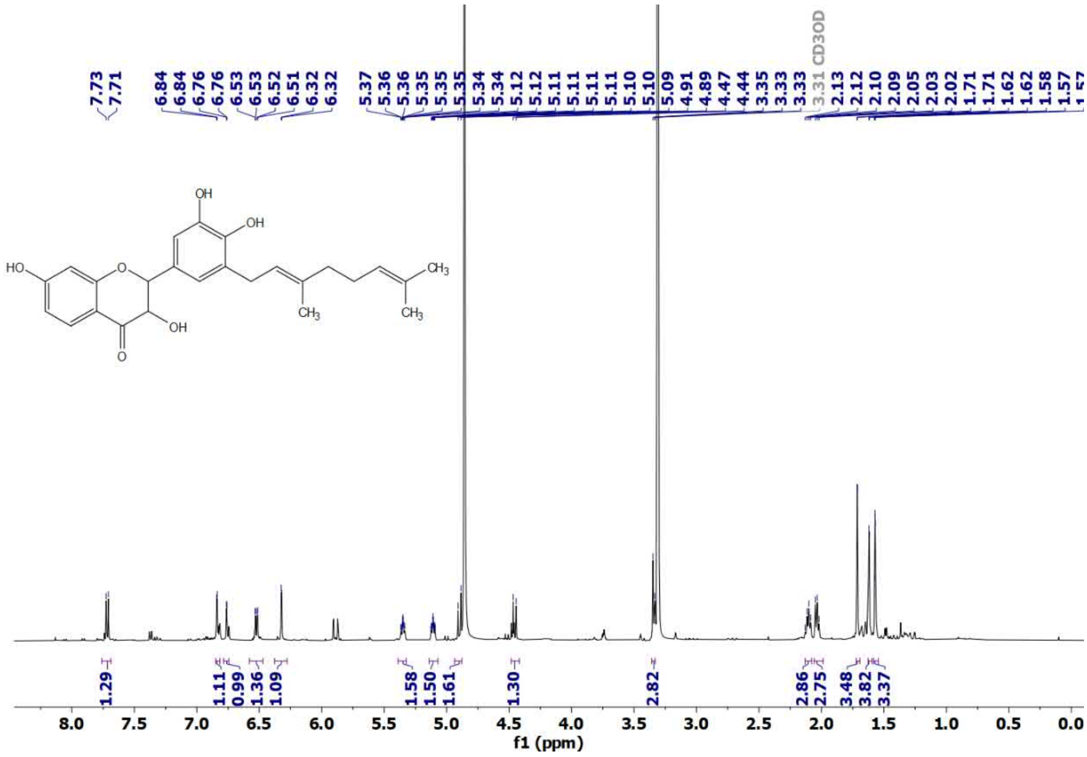

Figure S1. ^{1}H NMR (500 MHz, MeOH-d_4, 25 ºC) spectrum of gloverinol A (**1**)

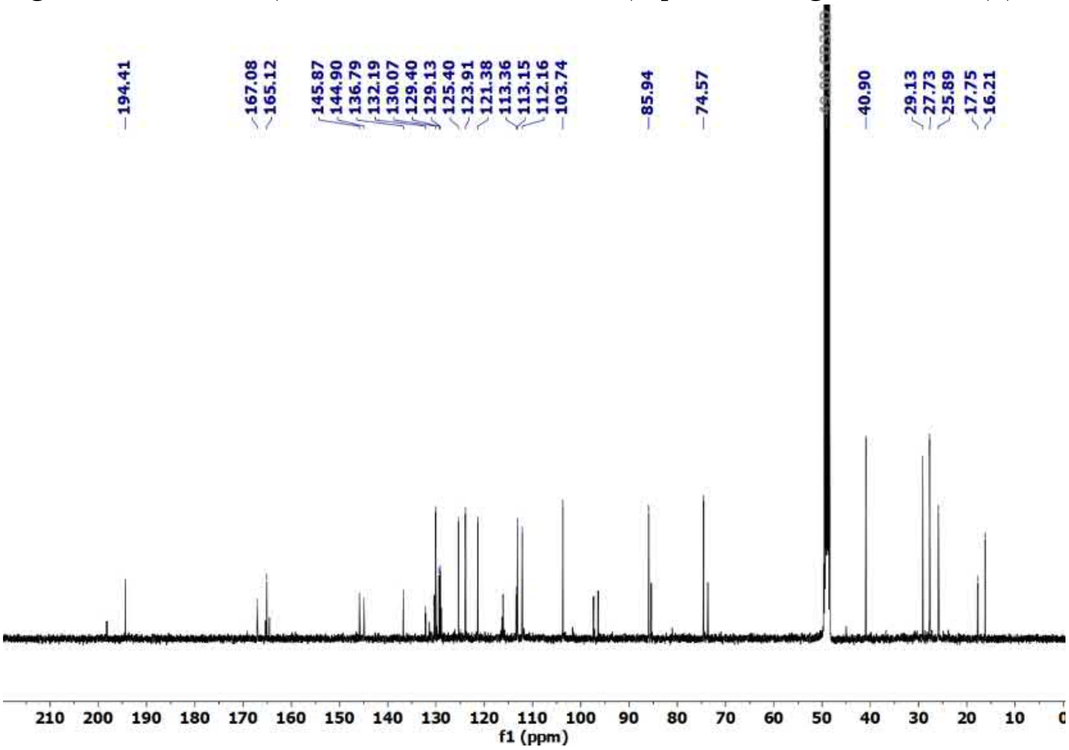

Figure S2. ^{13}C NMR (125 MHz, MeOH-d_4, 25 ºC) spectrum of gloverinol A (**1**)

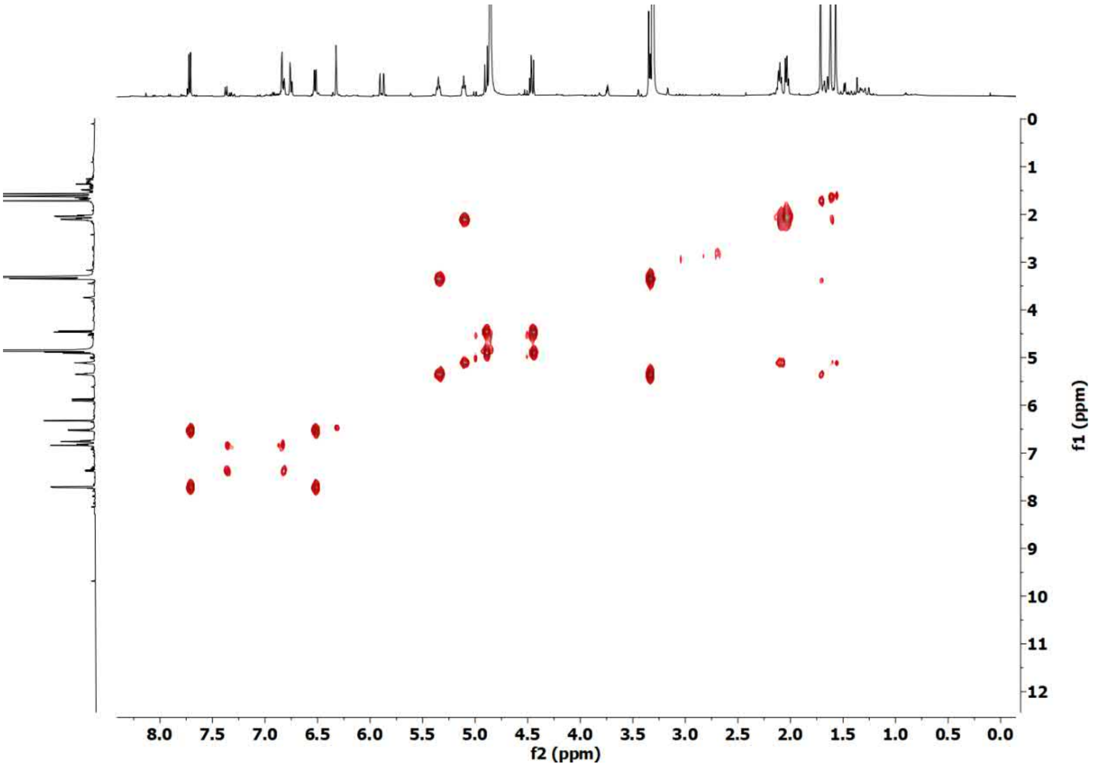

Figure S3. COSY (500 MHz, MeOH-d_4, 25 ºC) spectrum of gloverinol A (**1**)

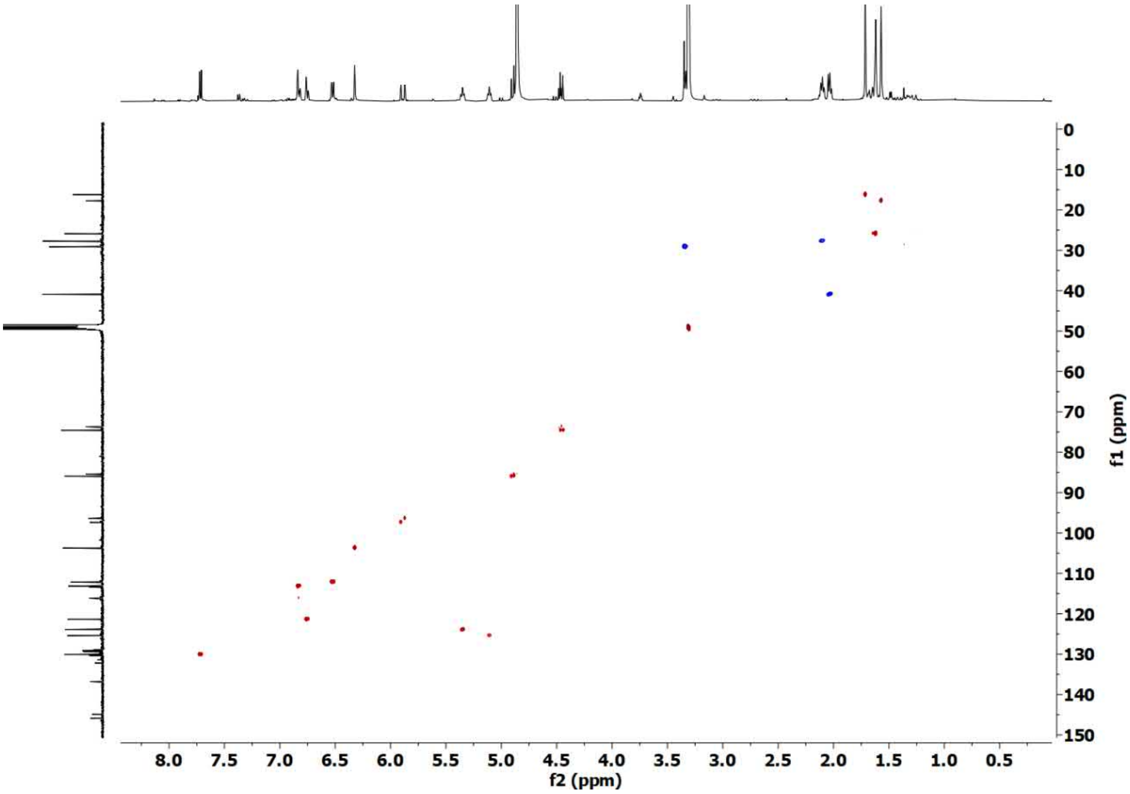

Figure S4. HSQC (500/125 MHz, MeOH-d_4, 25 ºC) spectrum of gloverinol A (**1**)

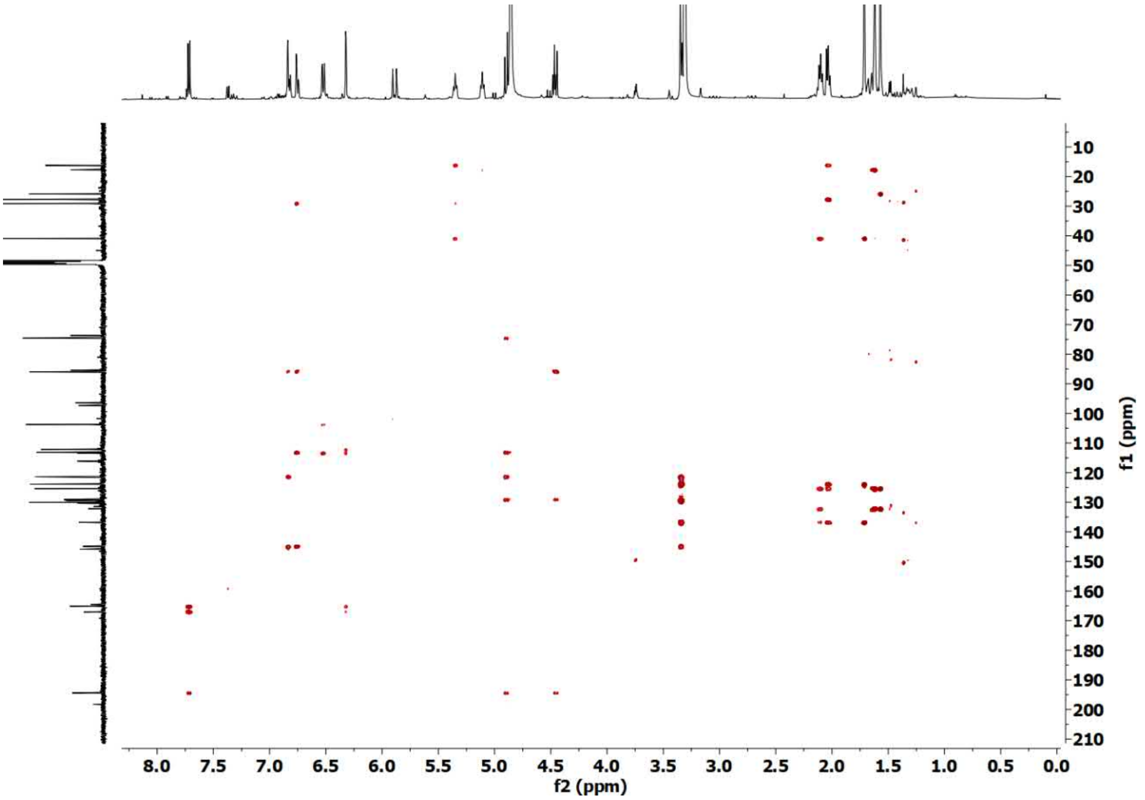

Figure S5. HMBC (500/125 MHz, MeOH-d_4, 25 ºC) spectrum of gloverinol A (**1**)

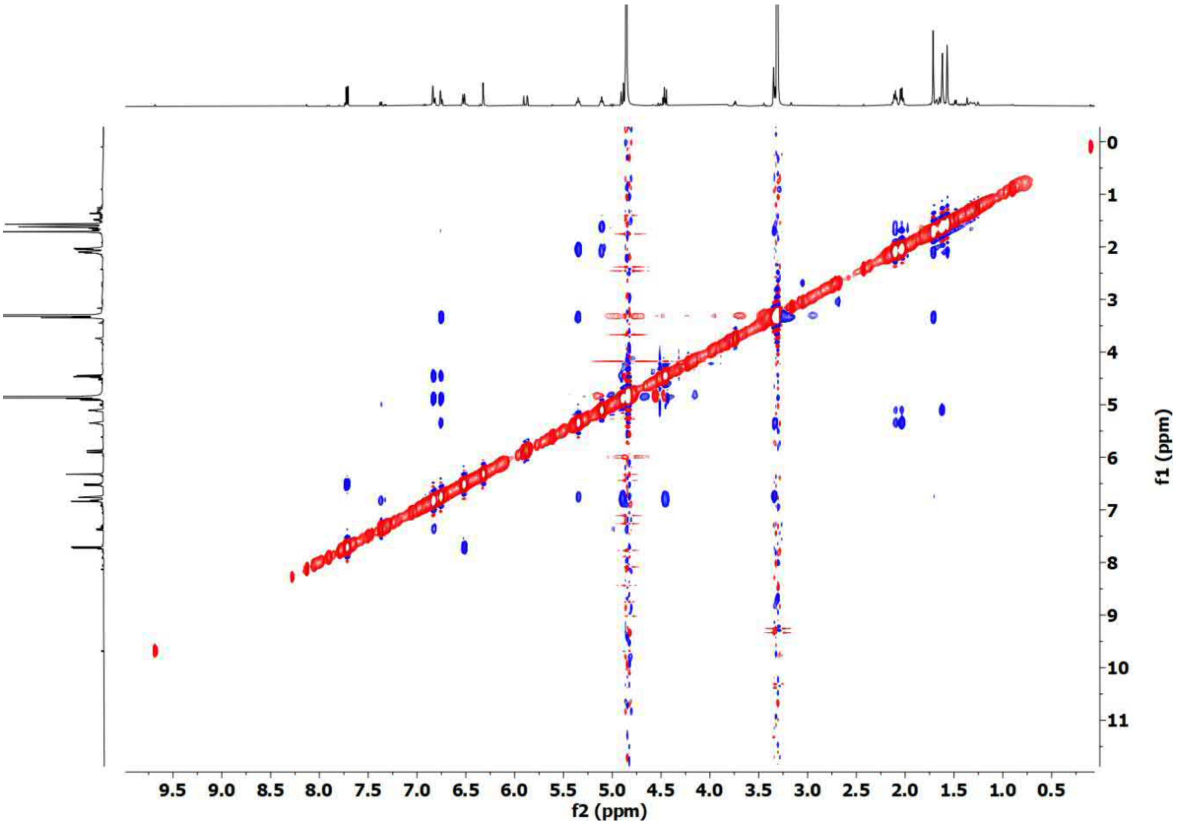

Figure S6. NOESY (500 MHz, MeOH-d_4, 25 ºC) spectrum of gloverinol A (**1**)

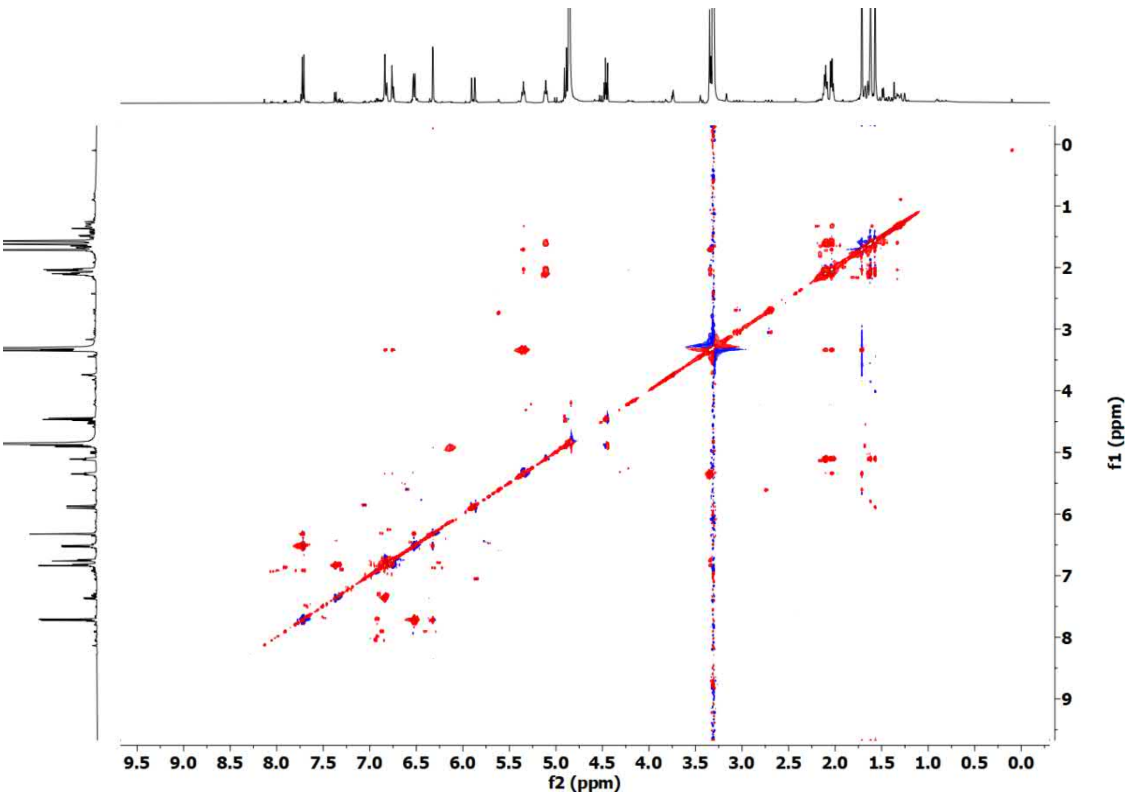

Figure S7. TOCSY (500 MHz, MeOH-d_4, 25 ºC) spectrum of gloverinol A (**1**)

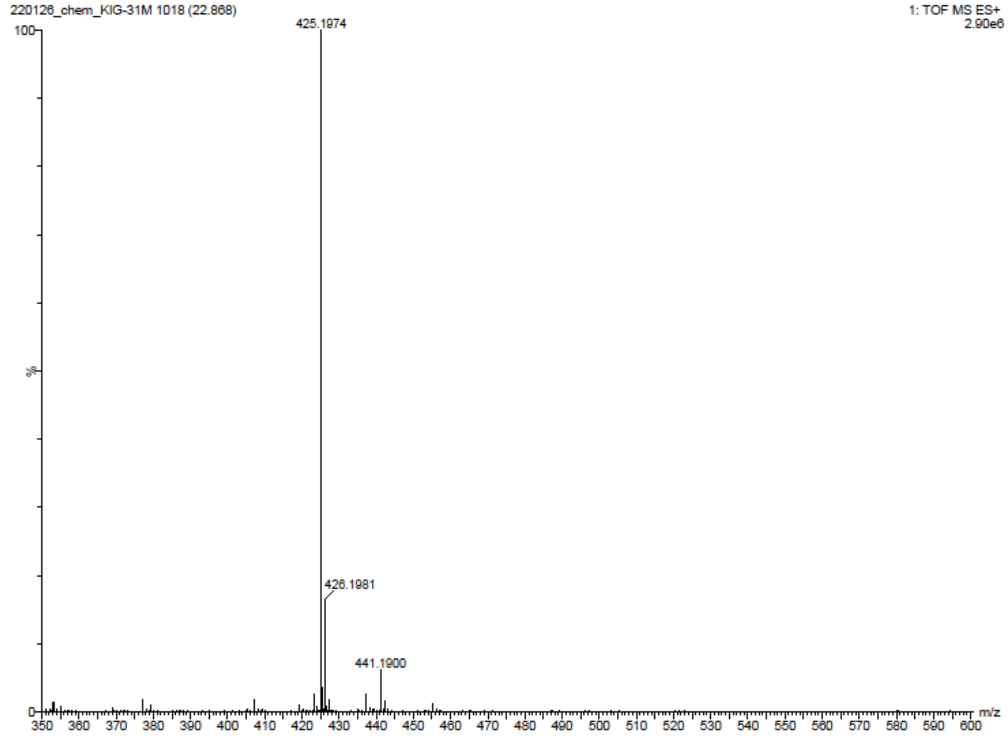

Figure S8. HRESIMS spectrum of gloverinol A (**1**)

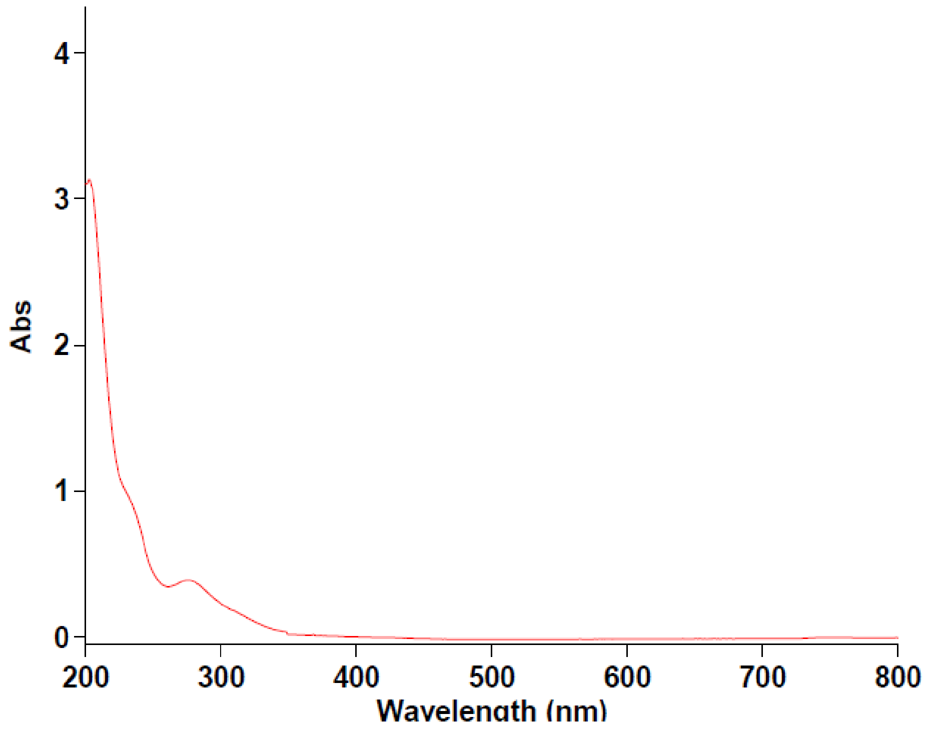

Figure S9. UV spectrum of gloverinol A (**1**)

Spectroscopic Data of gloverinonol B (2)

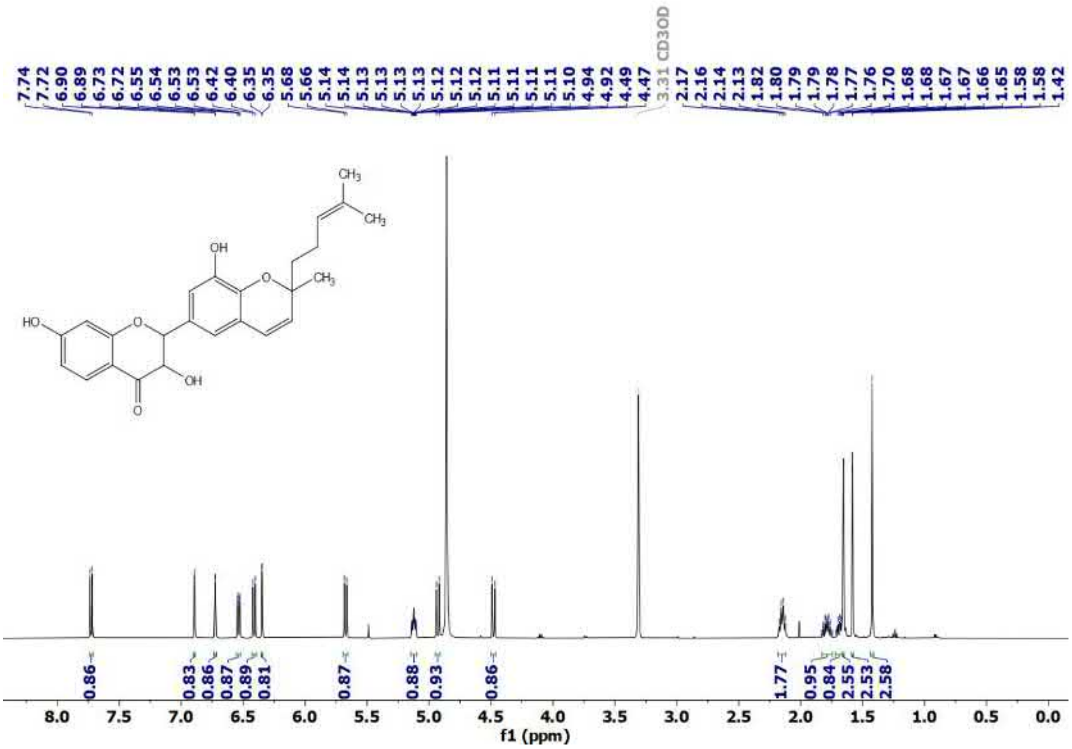

Figure S10. ^{1}H NMR (500 MHz, MeOH-d_4, 25 ºC) spectrum of gloverinol B (**2**)

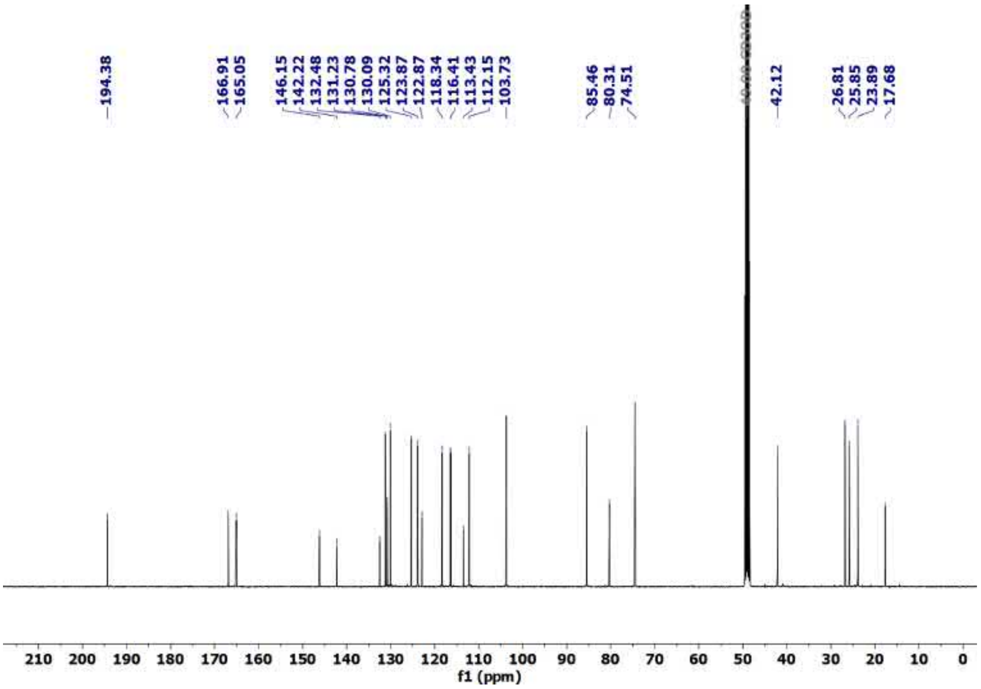

Figure S11. ^{13}C NMR (125 MHz, MeOH-d_4, 25 ºC) spectrum of gloverinol B (**2**)

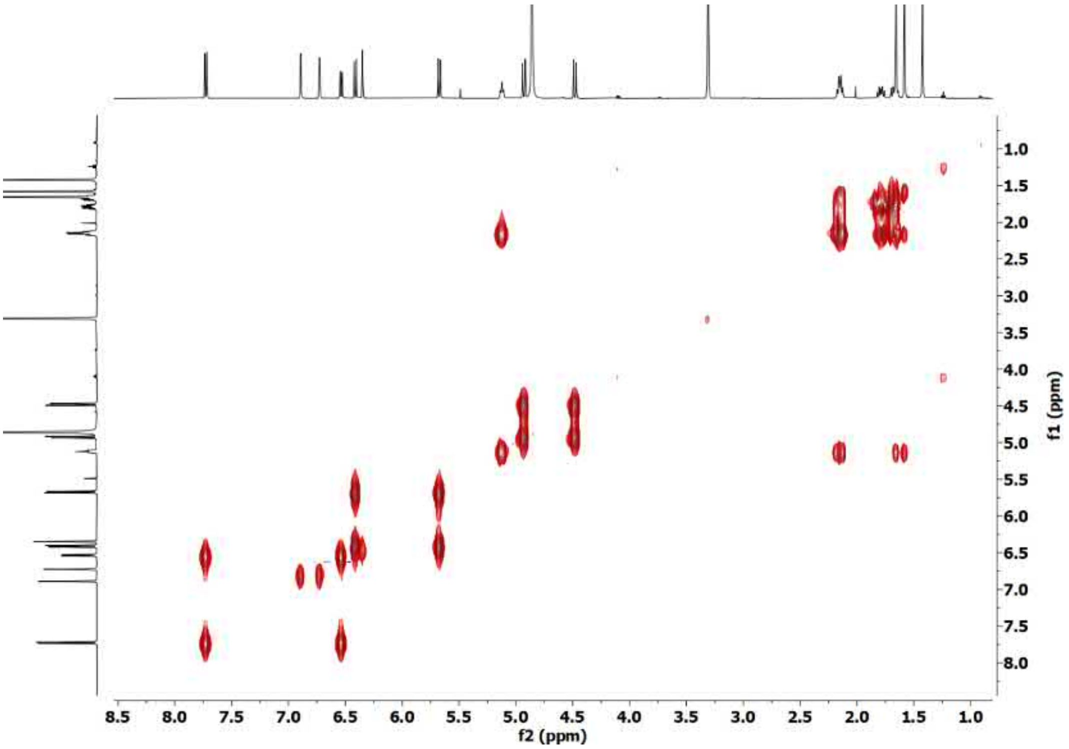

Figure S12. COSY (500 MHz, MeOH-d_4, 25 ºC) spectrum of gloverinol B (**2**)

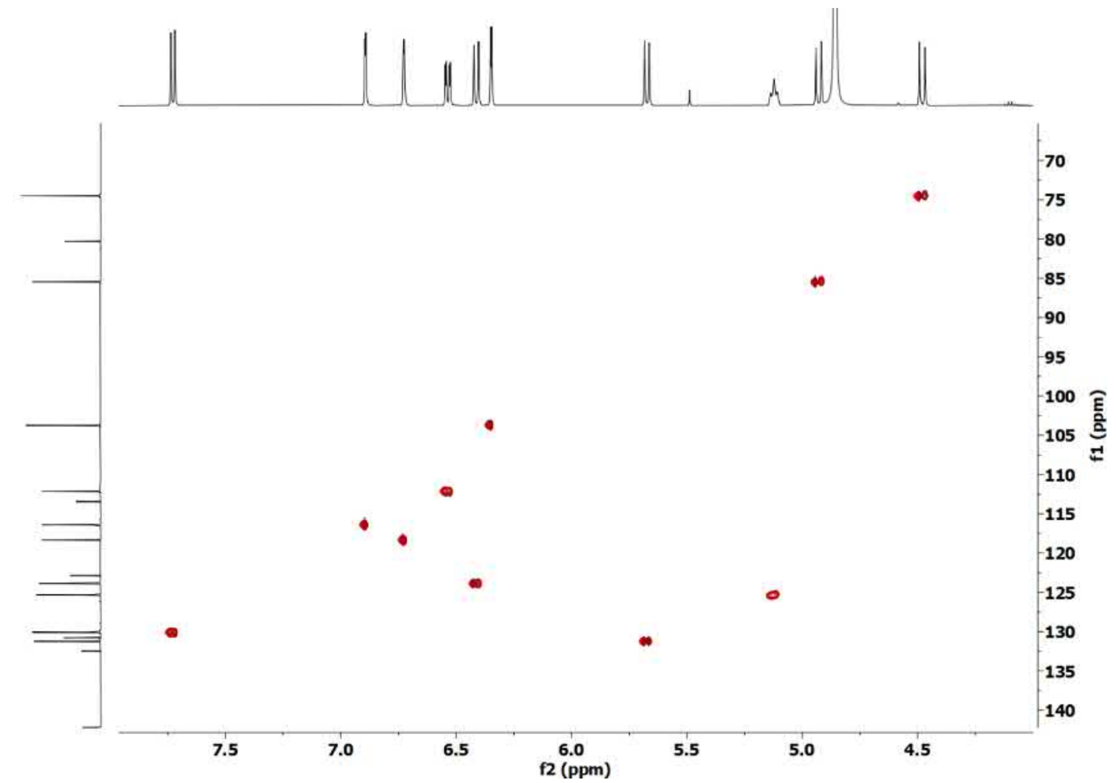

Figure S13. HSQC (500/125 MHz, MeOH-d_4, 25 ºC) spectrum of gloverinol B (**2**)

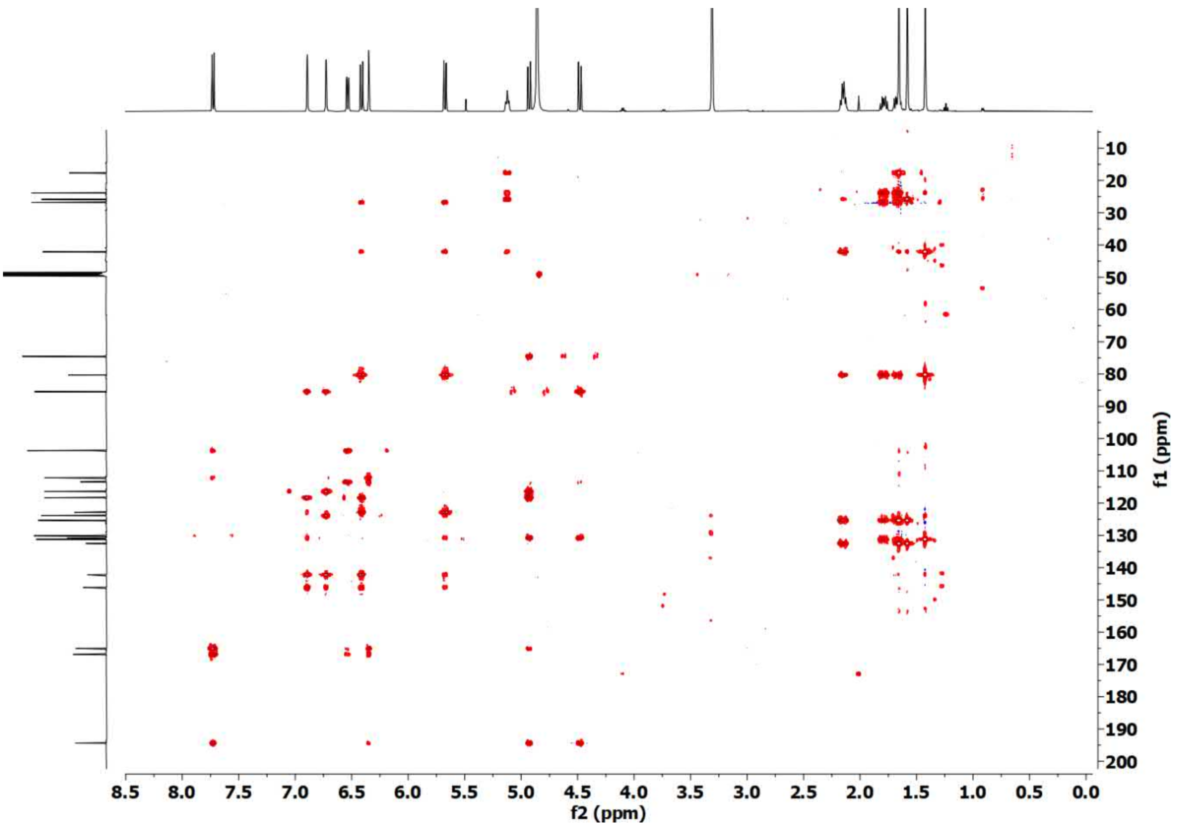

Figure S14: HMBC (500/125 MHz, MeOH-d_4, 25 ºC) spectrum of gloverinol B (**2**)

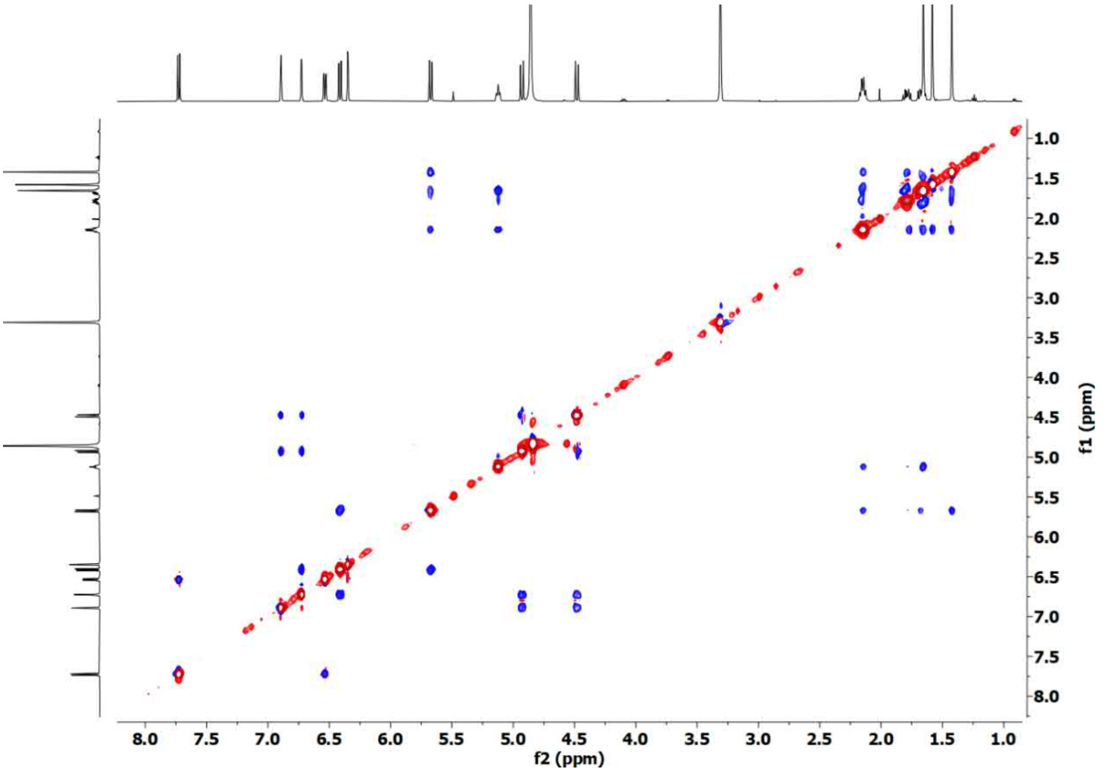

Figure S15. NOESY (500 MHz, MeOH-d_4, 25 ºC) spectrum of gloverinol B (**2**)

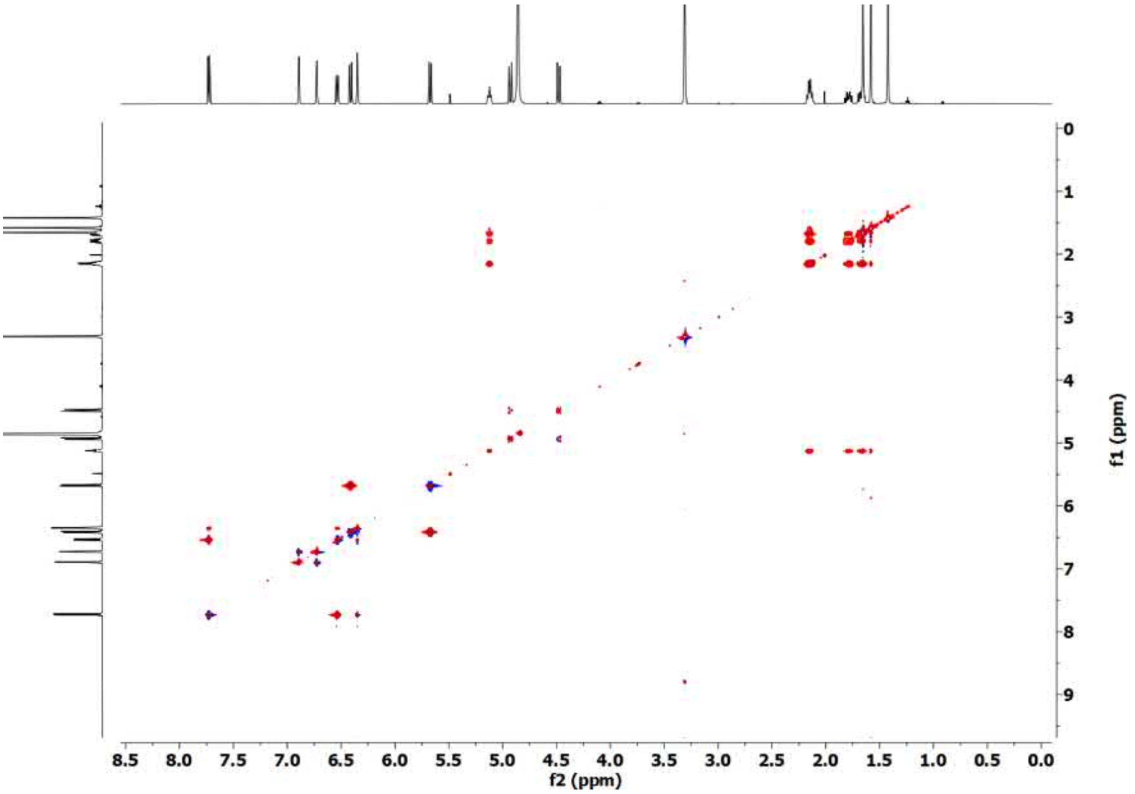

Figure S16. TOCSY (500 MHz, MeOH-d_4, 25 ºC) spectrum of gloverinol B (**1**)

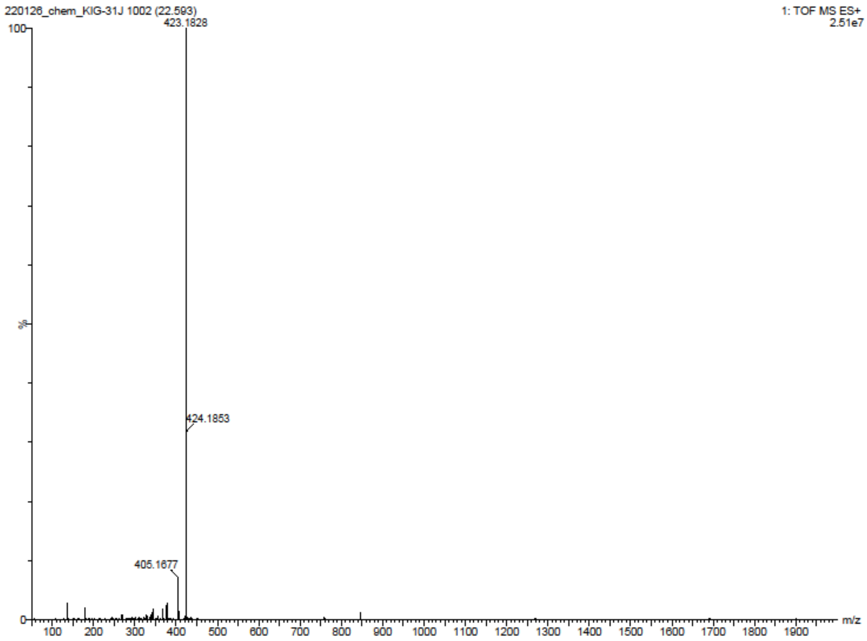

Figure S17. HRESIMS spectrum of gloverinol B (**2**)

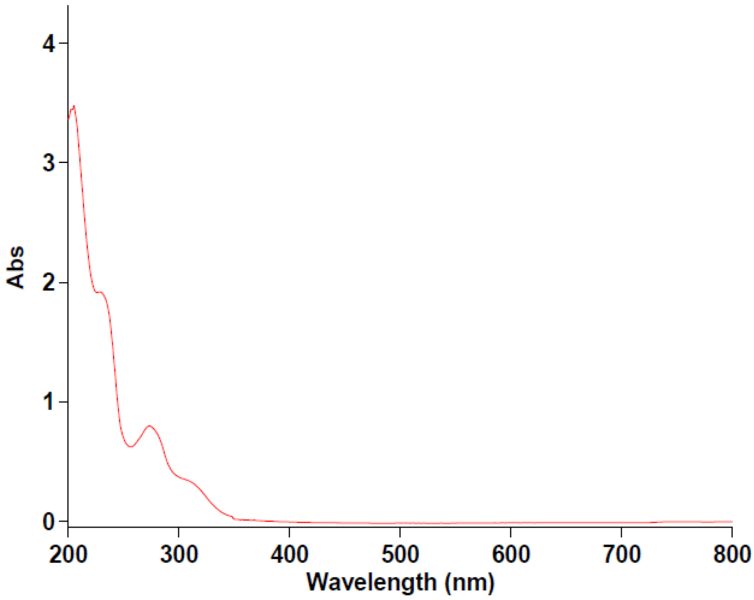

Figure S18. UV spectrum of gloverinol B (**2**)

Spectroscopic Data of gloverinol C (3)

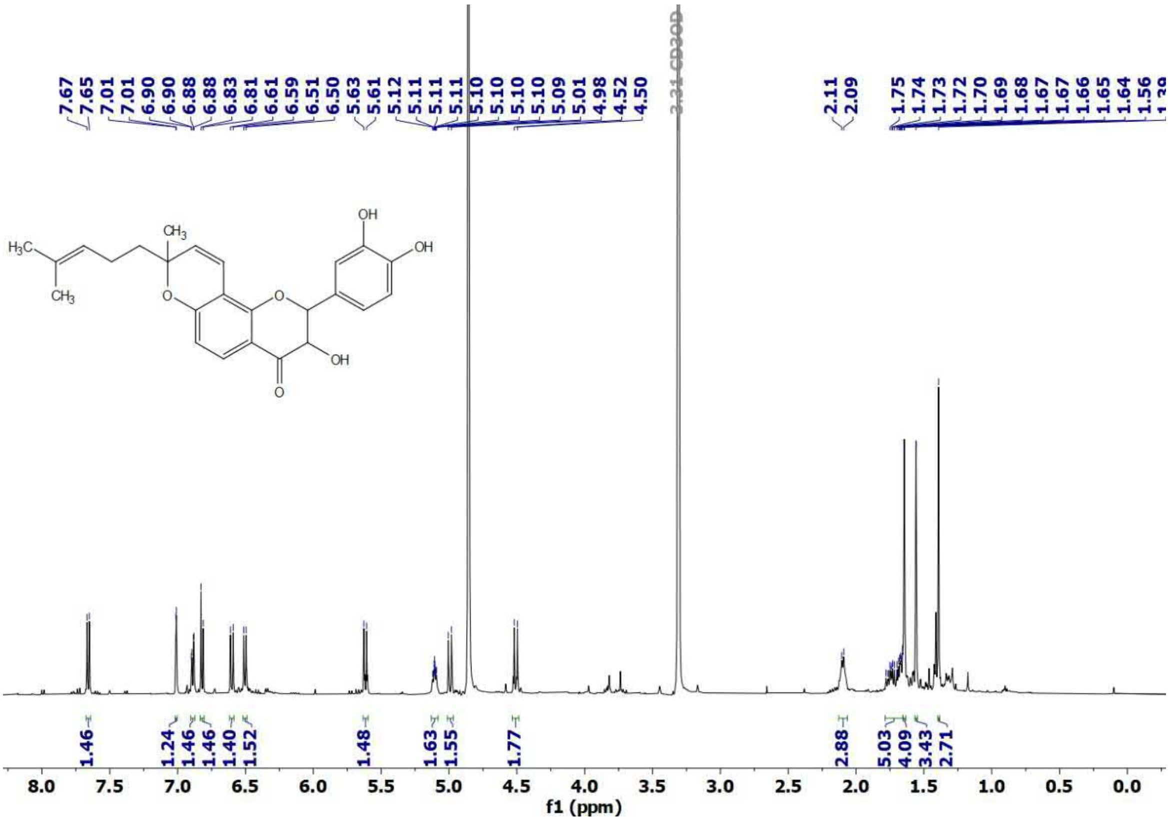

Figure S19. ^{1}H NMR (500 MHz, MeOH-d_4, 25 ºC) spectrum of gloverinol C (**3**)

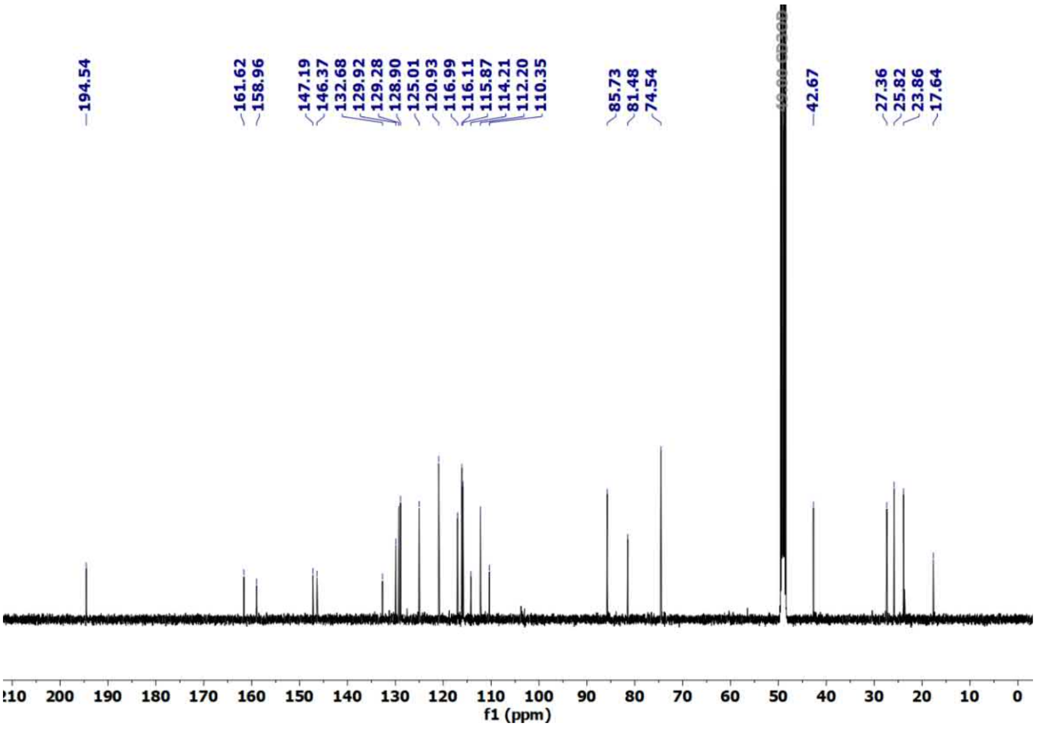

Figure S20. ^{13}C NMR (125 MHz, MeOH-d_4, 25 ºC) spectrum of gloverinol C (**3**)

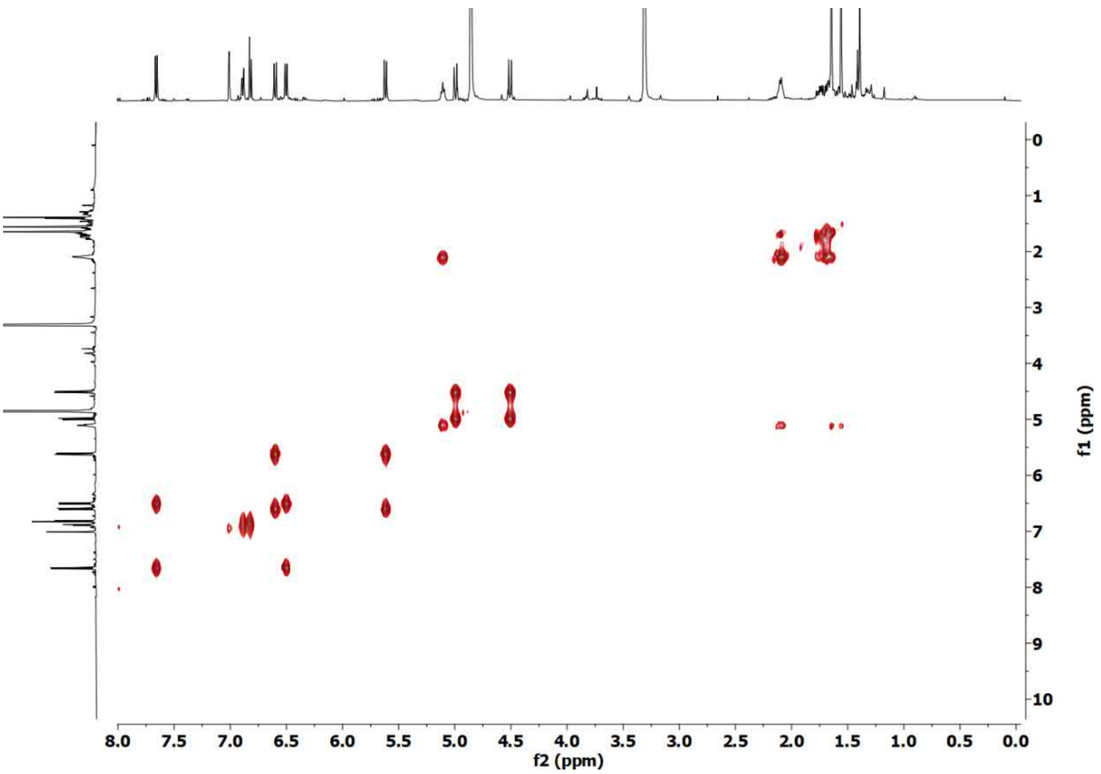

Figure S21. COSY (500 MHz, MeOH-d_4, 25 ºC) spectrum of gloverinol C (**3**)

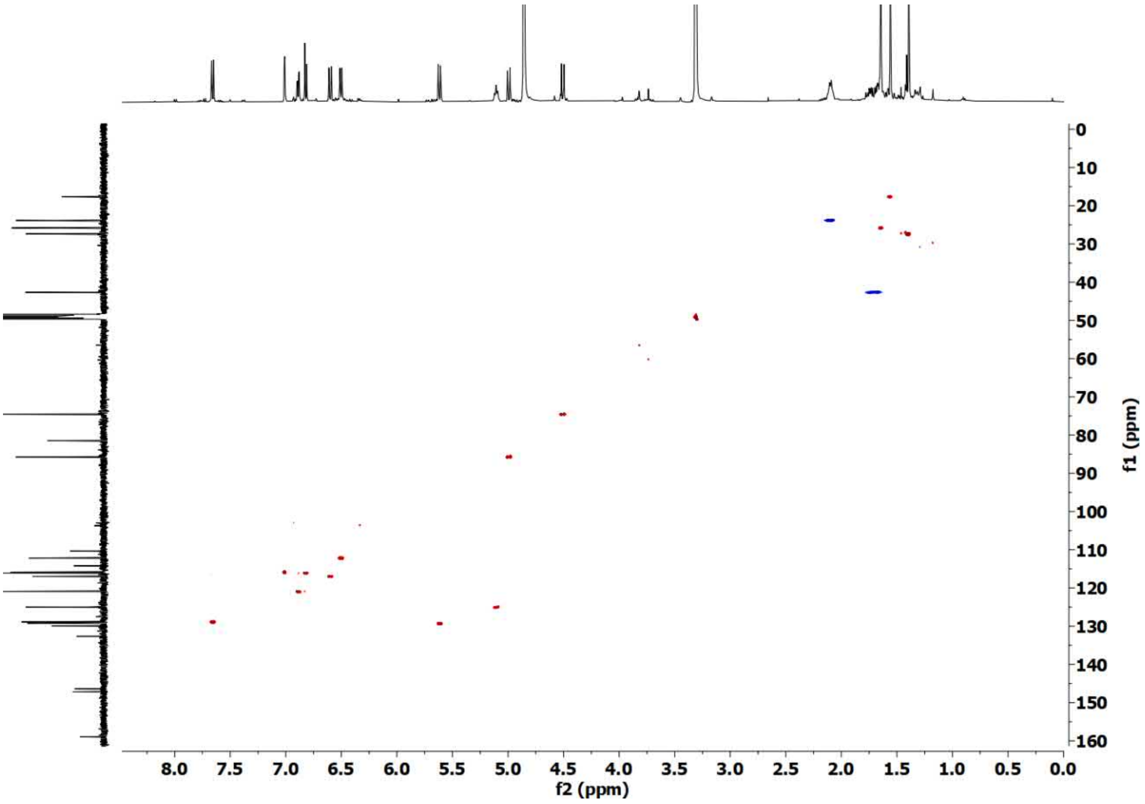

Figure S22. HSQC (500/125 MHz, MeOH-d_4, 25 ºC) spectrum of gloverinol C (**3**)

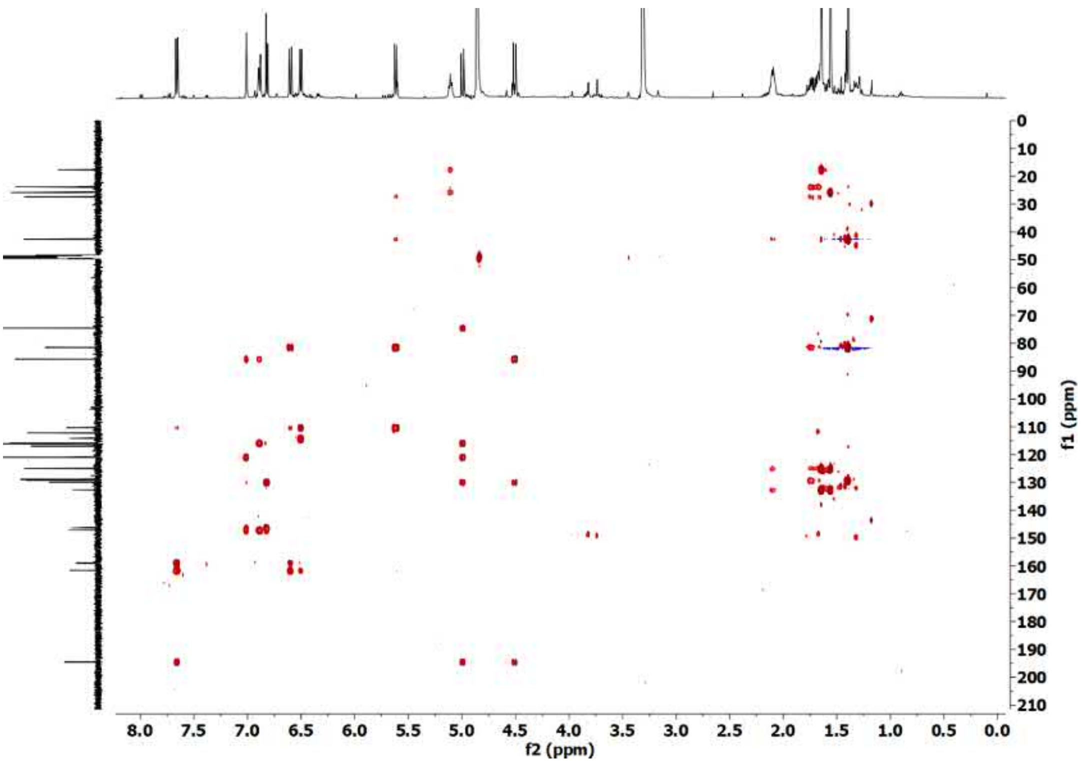

Figure S23. HMBC (500/125 MHz, MeOH-d_4, 25 ºC) of gloverinol C (**3**)

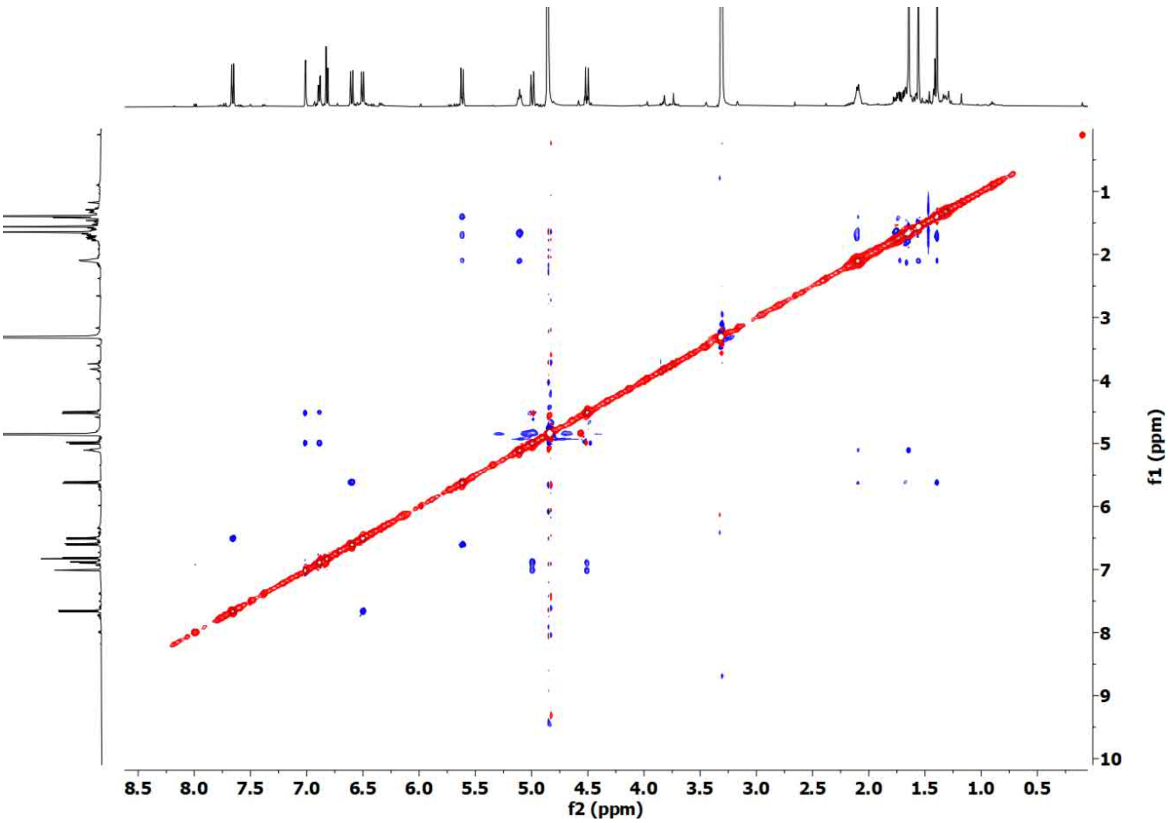

Figure S24. NOESY (500 MHz, MeOH-d_4, 25 ºC) spectrum of gloverinol C (**3**)

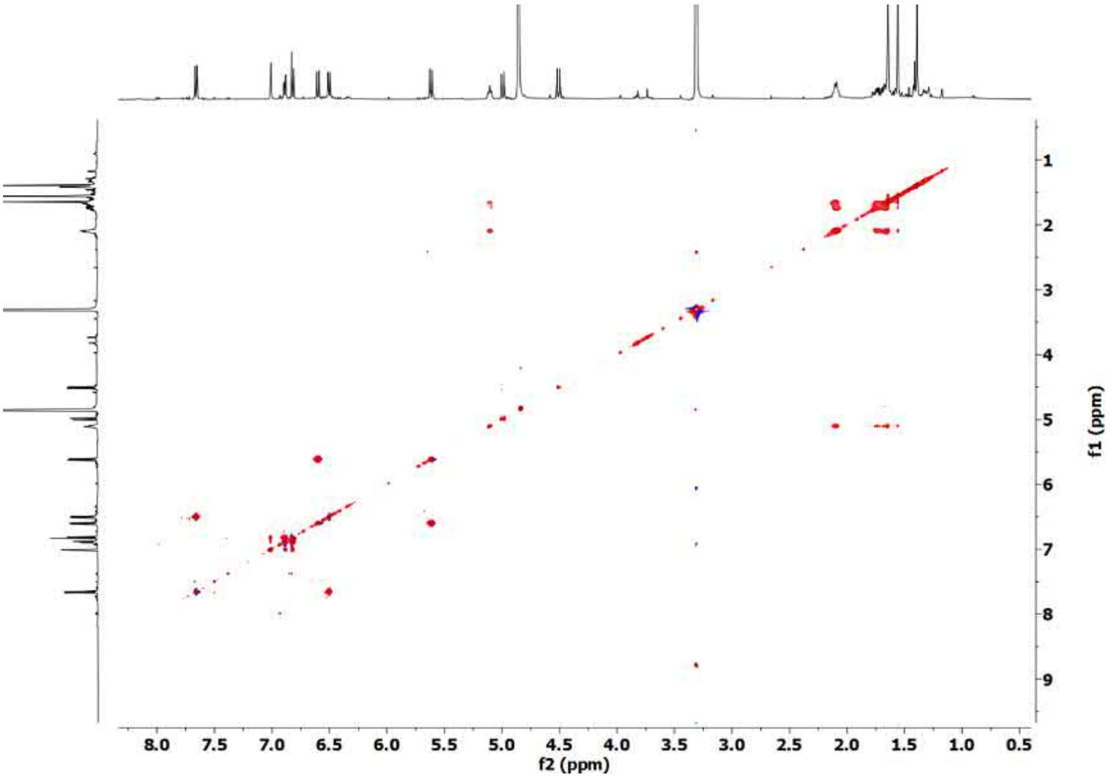

Figure S25. TOCSY (500 MHz, MeOH-d_4, 25 ºC) spectrum of gloverinol B (**1**)

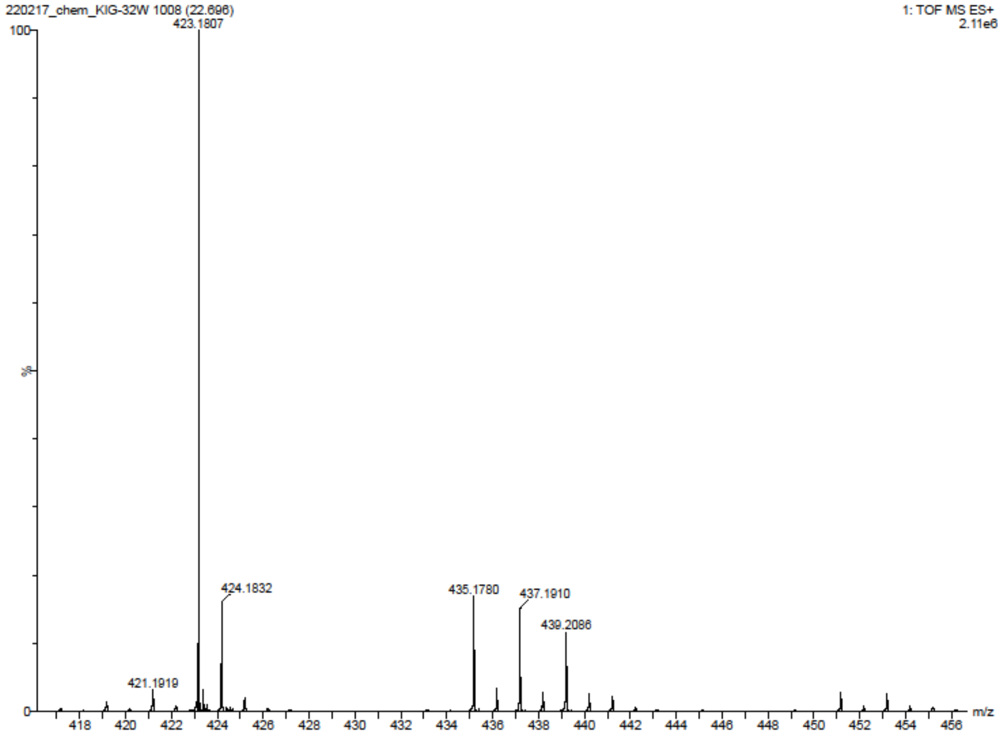

Figure S26. HRESIMS spectrum of gloverinol C (**3**)

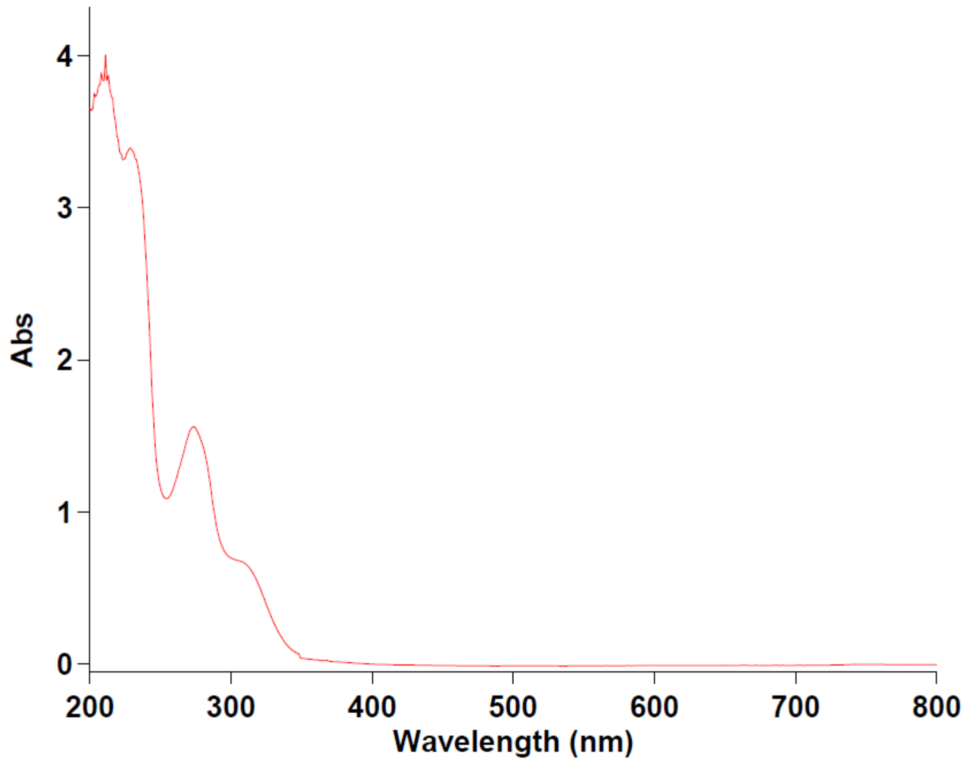

Figure S27. UV spectrum of gloverinol C (**3**)

Spectroscopic Data of gloverinol D (4)

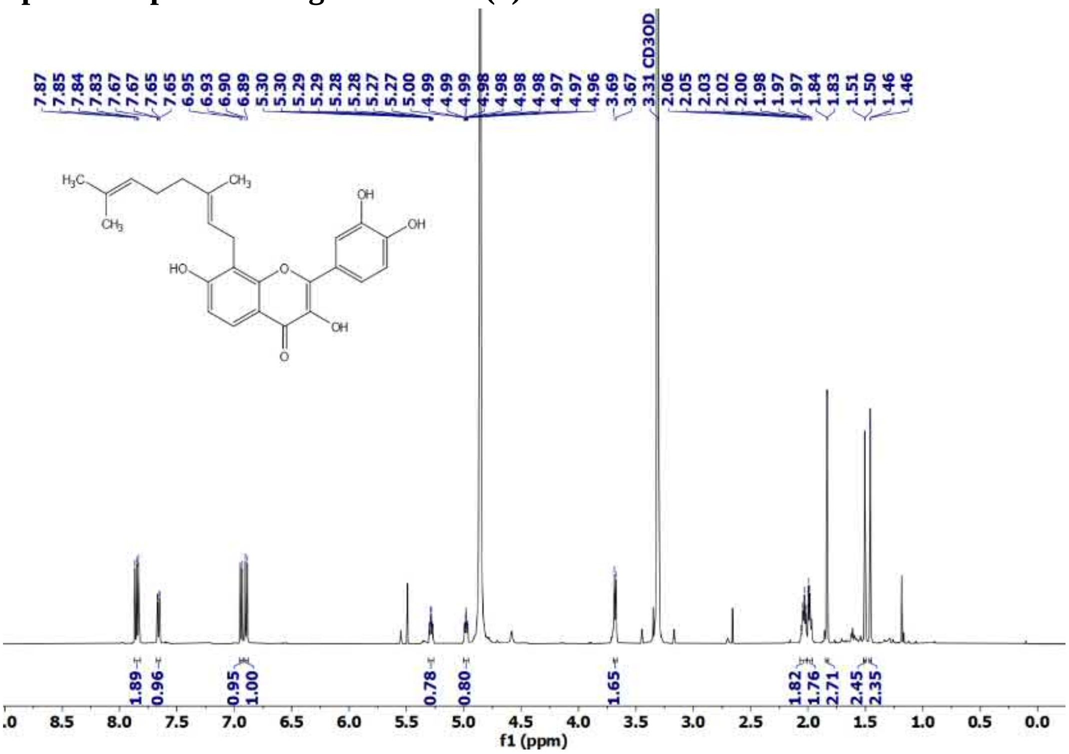

Figure S28. ^{1}H NMR (500 MHz, MeOH-d_4, 25 ºC) spectrum of gloverinol D (**4**)

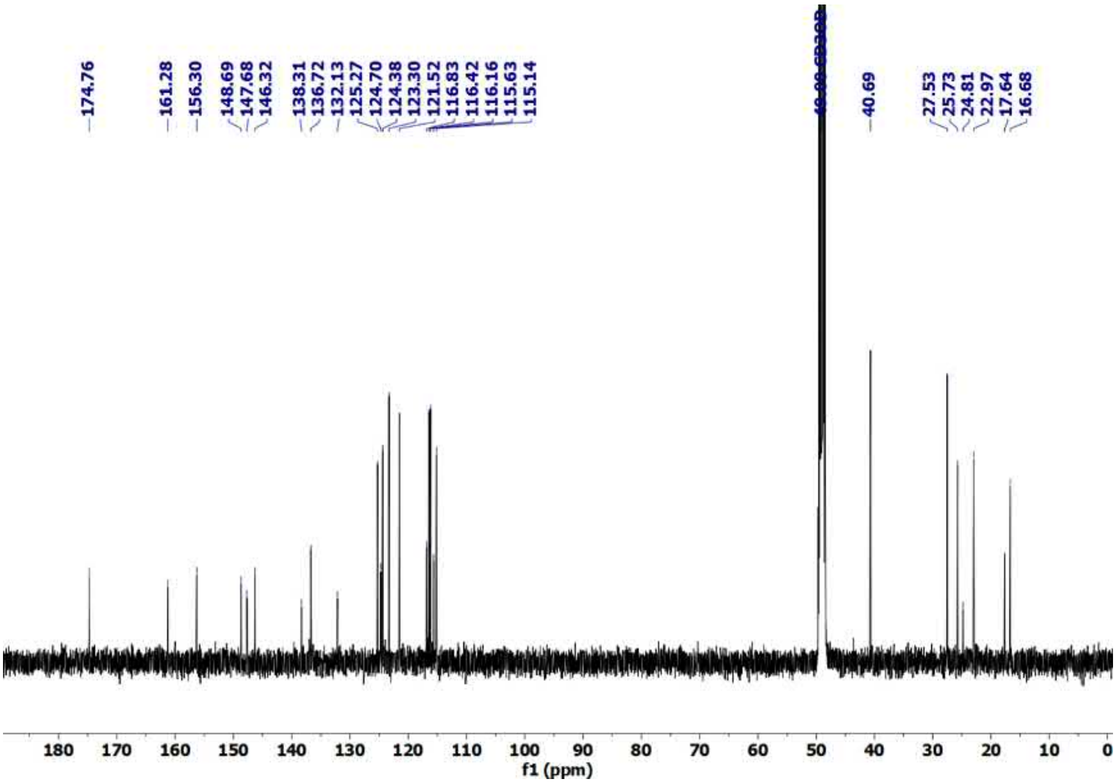

Figure S29. ^{13}C NMR (125 MHz, MeOH-d_4, 25 ºC) spectrum of gloverinol D (**4**)

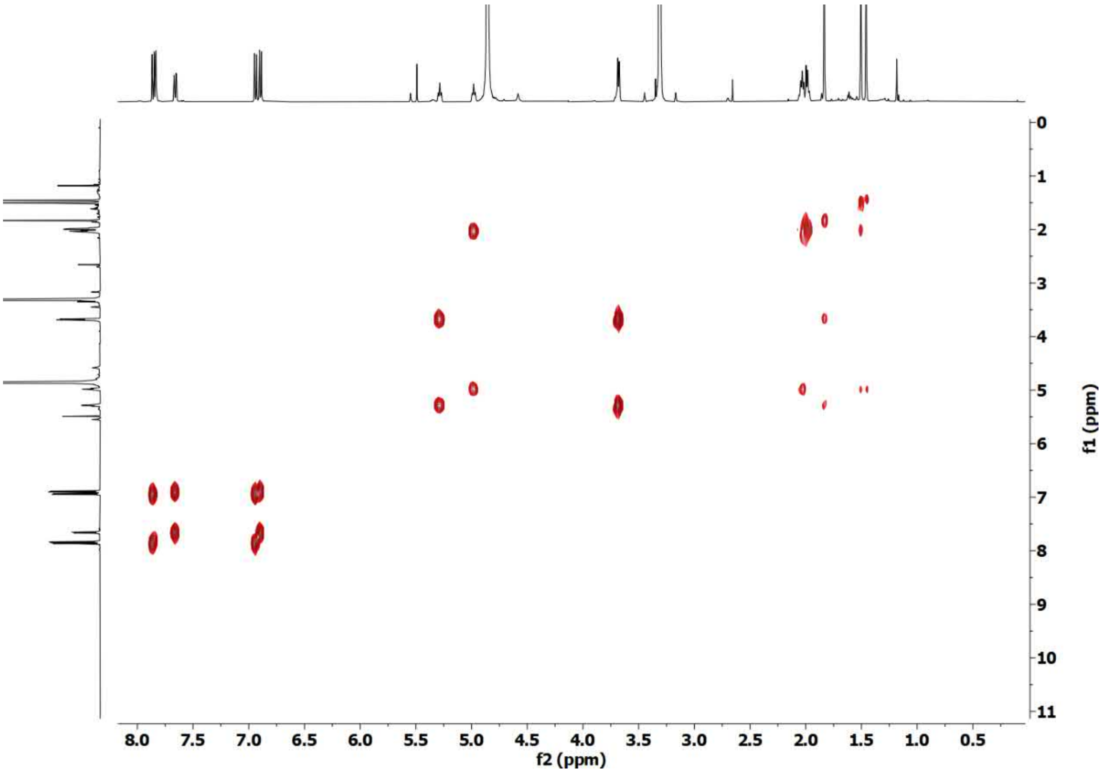

Figure S30. COSY (500 MHz, MeOH-d_4, 25 ºC) spectrum of gloverinol D (**4**)

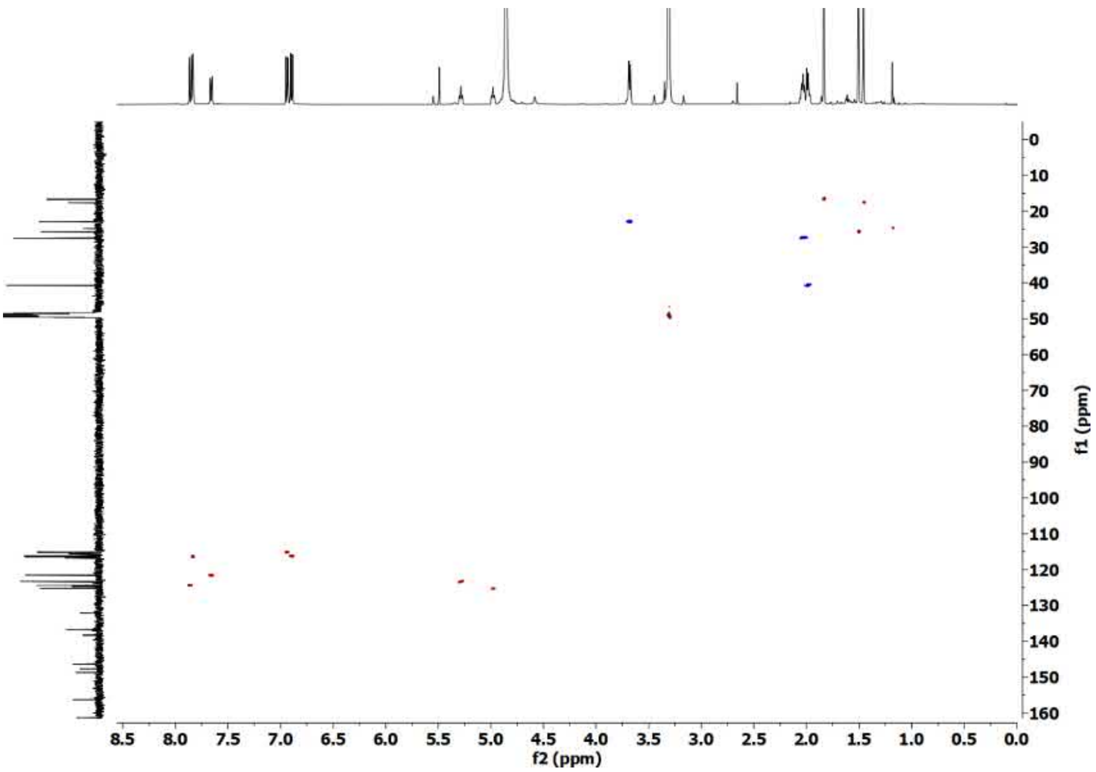

Figure S31. HSQC (500/125 MHz, MeOH-d_4, 25 ºC) spectrum of gloverinol D (**4**)

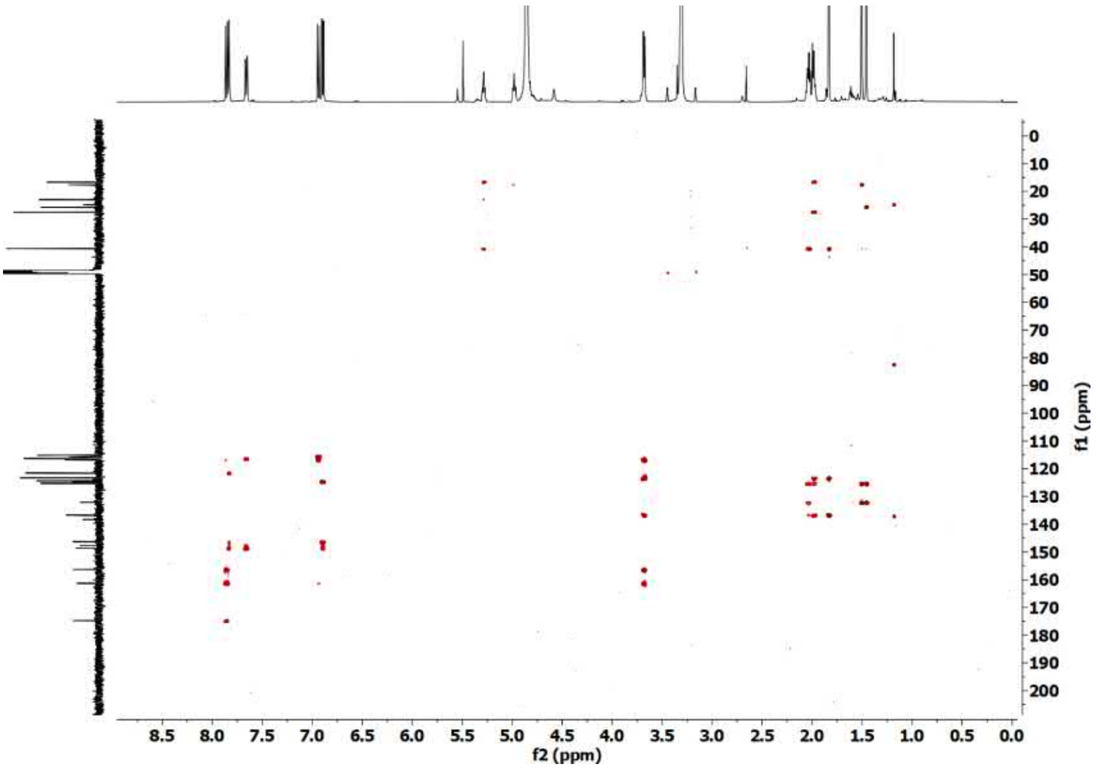

Figure S32. HMBC (500/125 MHz, MeOH-d_4, 25 ºC) spectrum of gloverinol D (**4**)

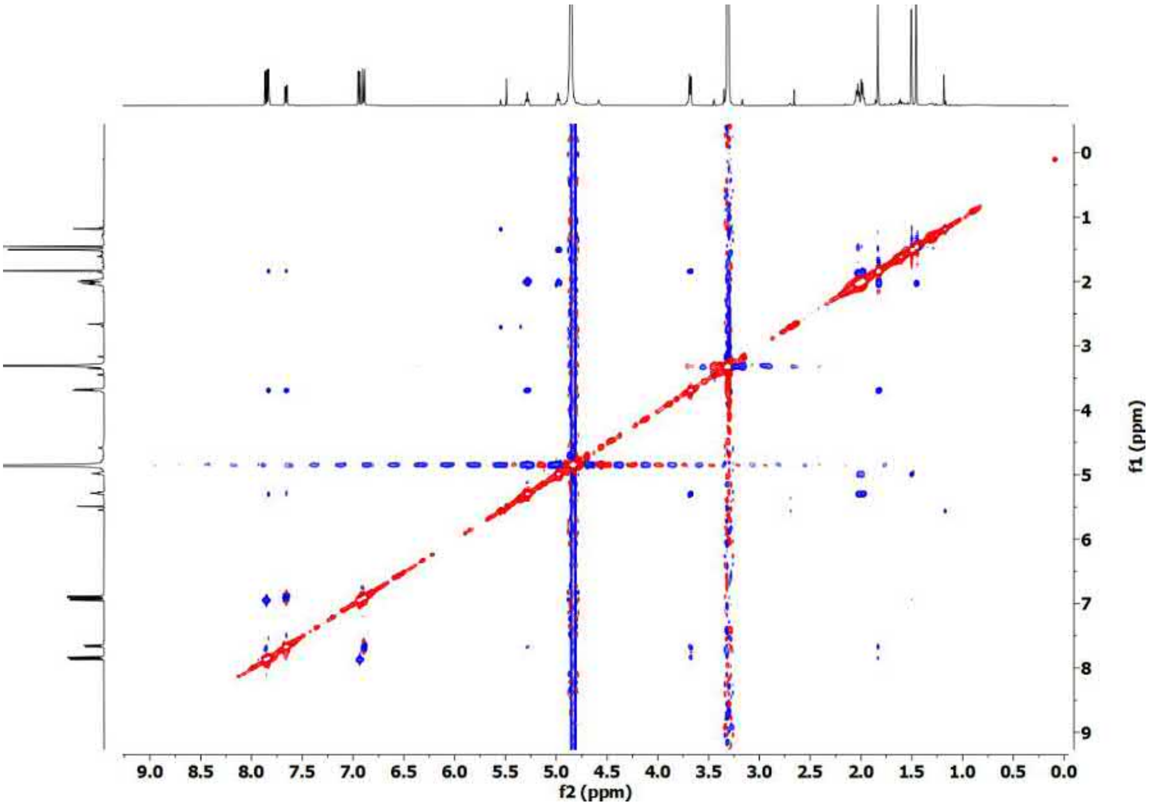

Figure S33. NOESY (500 MHz, MeOH-d_4, 25 ºC) spectrum of gloverinol D (**4**)

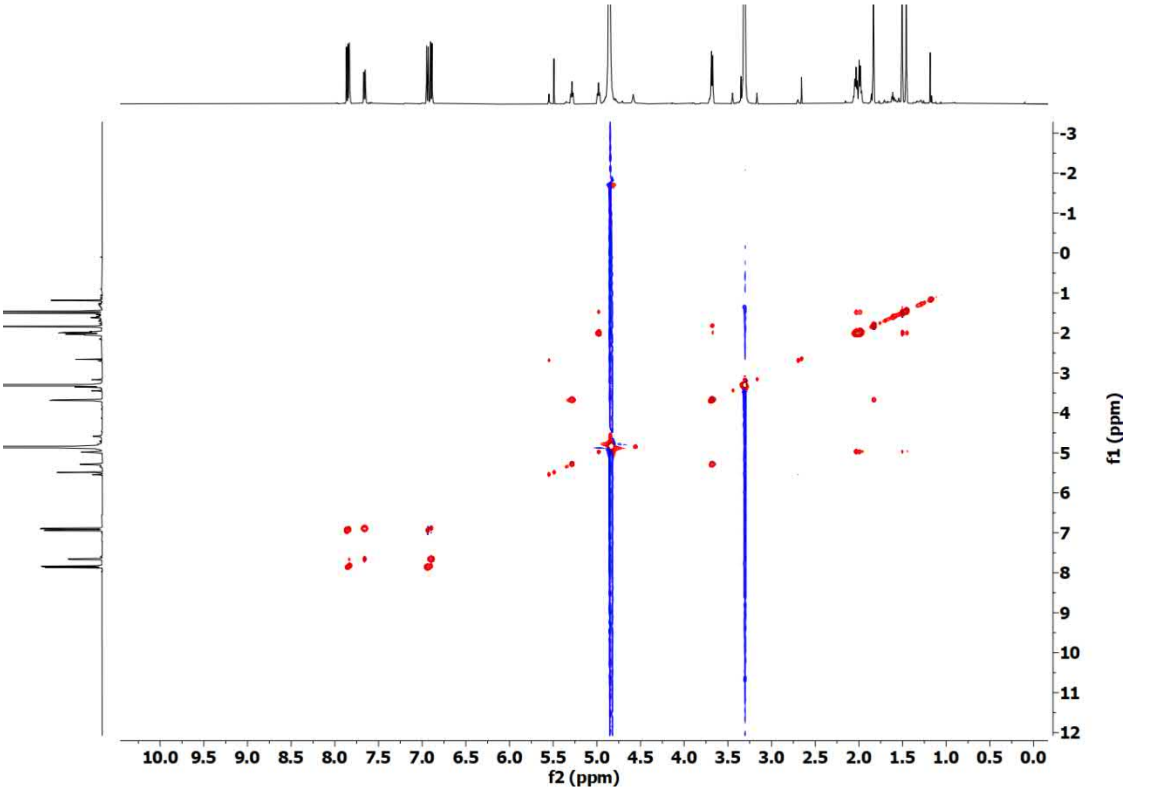

Figure S34. TOCSY (500 MHz, MeOH-d_4, 25 ºC) spectrum of gloverinol D (**4**)

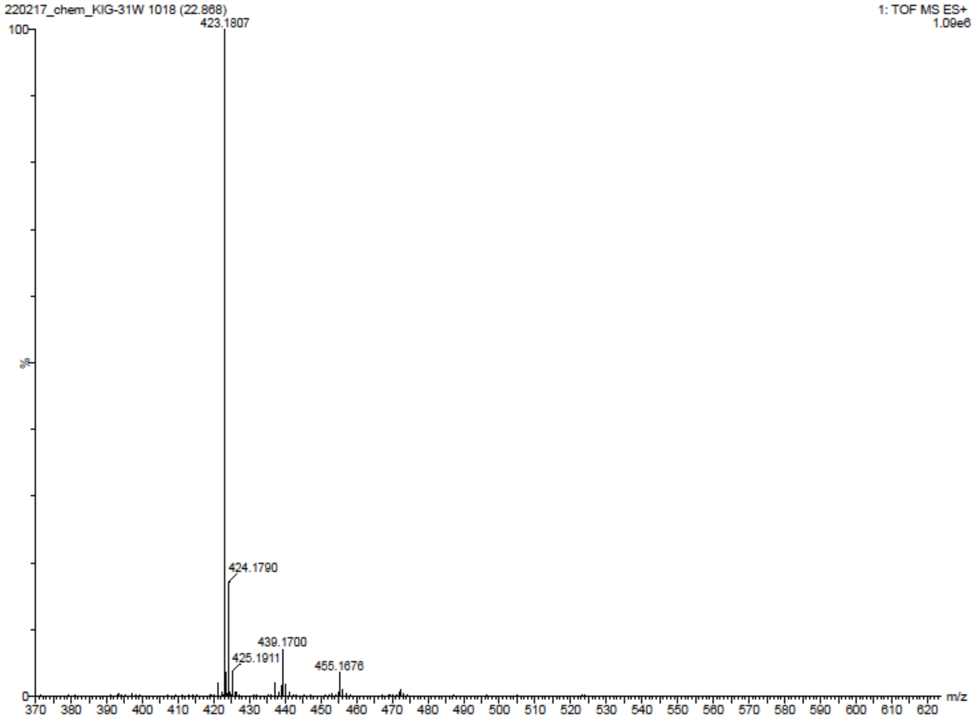

Figure S35. HRESIMS spectrum of gloverinol D (**4**)

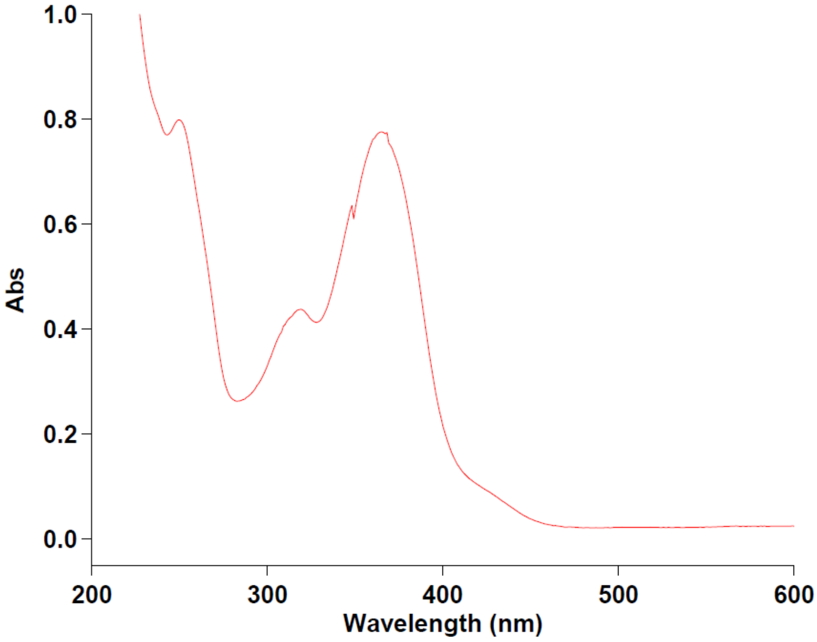

Figure S36. UV spectrum of gloverinol D (**4**)

Spectroscopic Data of gloveriflavan A (5)

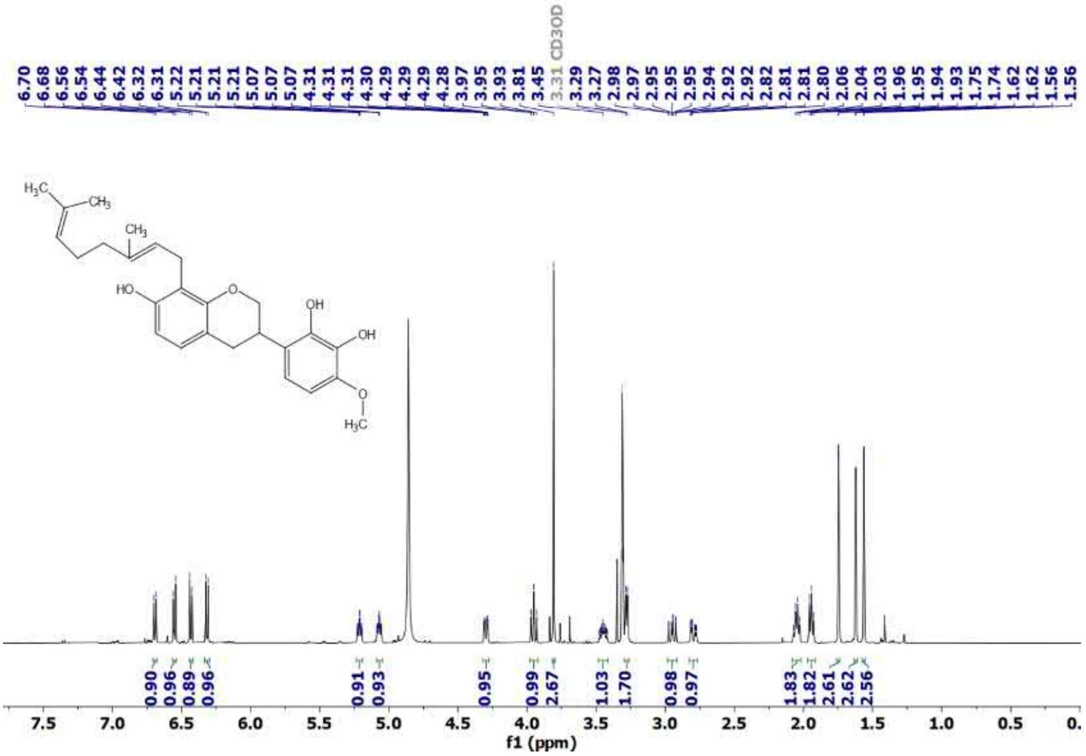

Figure S37. ^{1}H NMR (500 MHz, MeOH-d_4, 25 ºC) spectrum of gloveriflavan A (**5**)

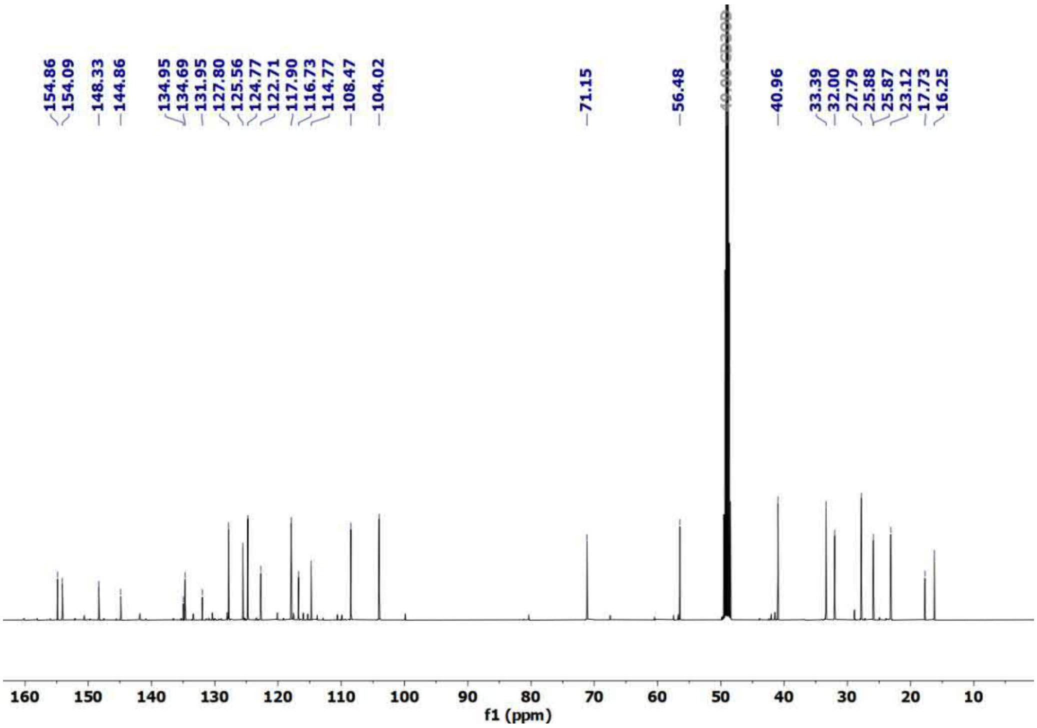

Figure S38. ^{13}C NMR (125 MHz, MeOH-d_4, 25 ºC) spectrum of gloveriflavan A (**5**)

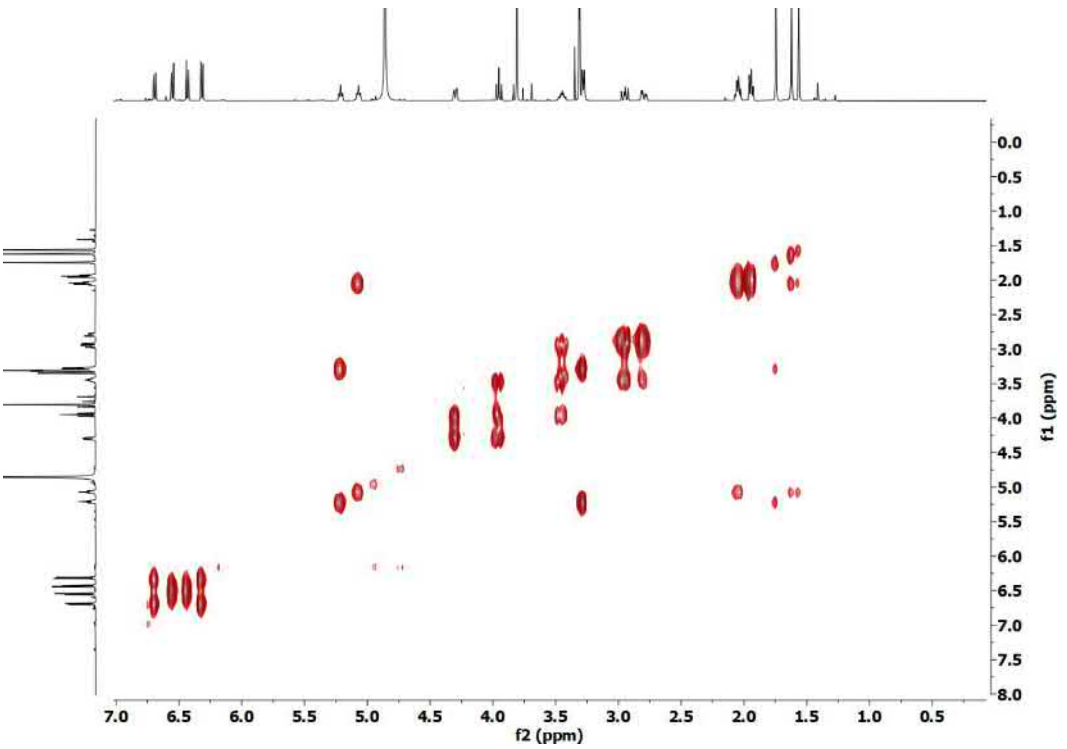

Figure S39. COSY (500 MHz, MeOH-d_4, 25 ºC) spectrum of gloveriflavan A (**5**)

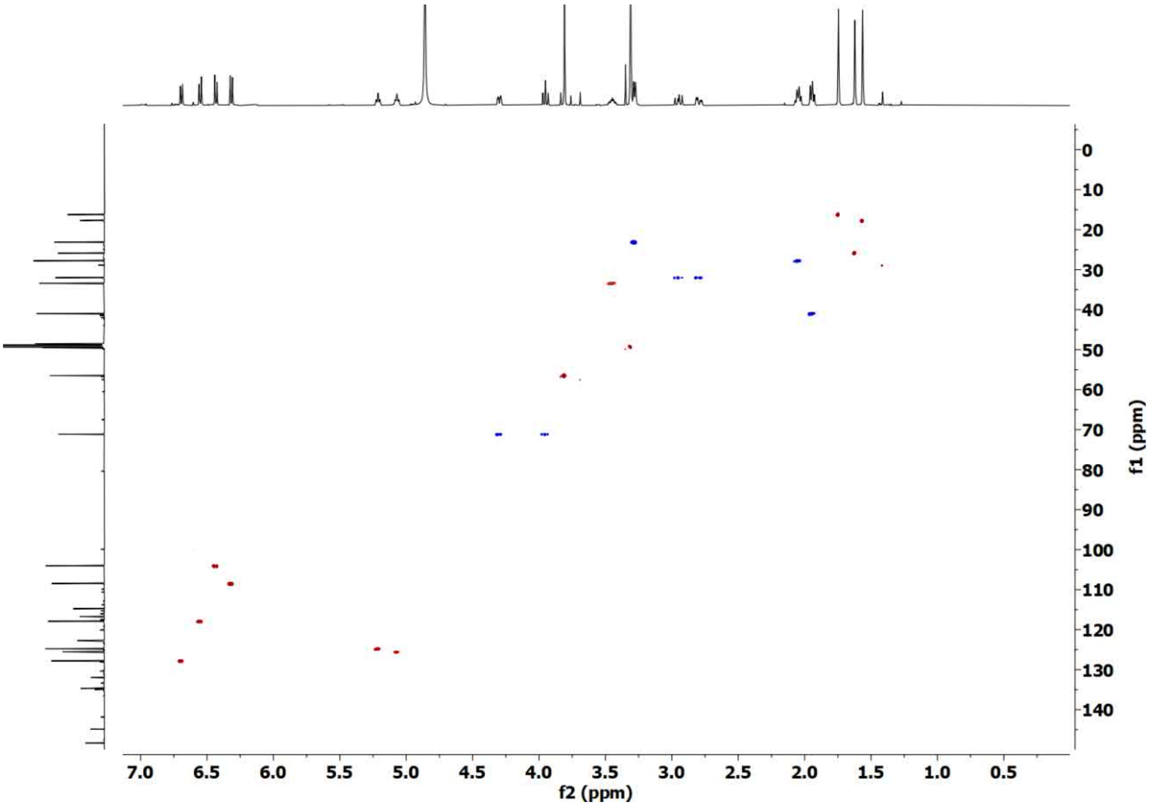

Figure S40. HSQC (500/125 MHz, MeOH-d_4, 25 ºC) spectrum of gloveriflavan A (**5**)

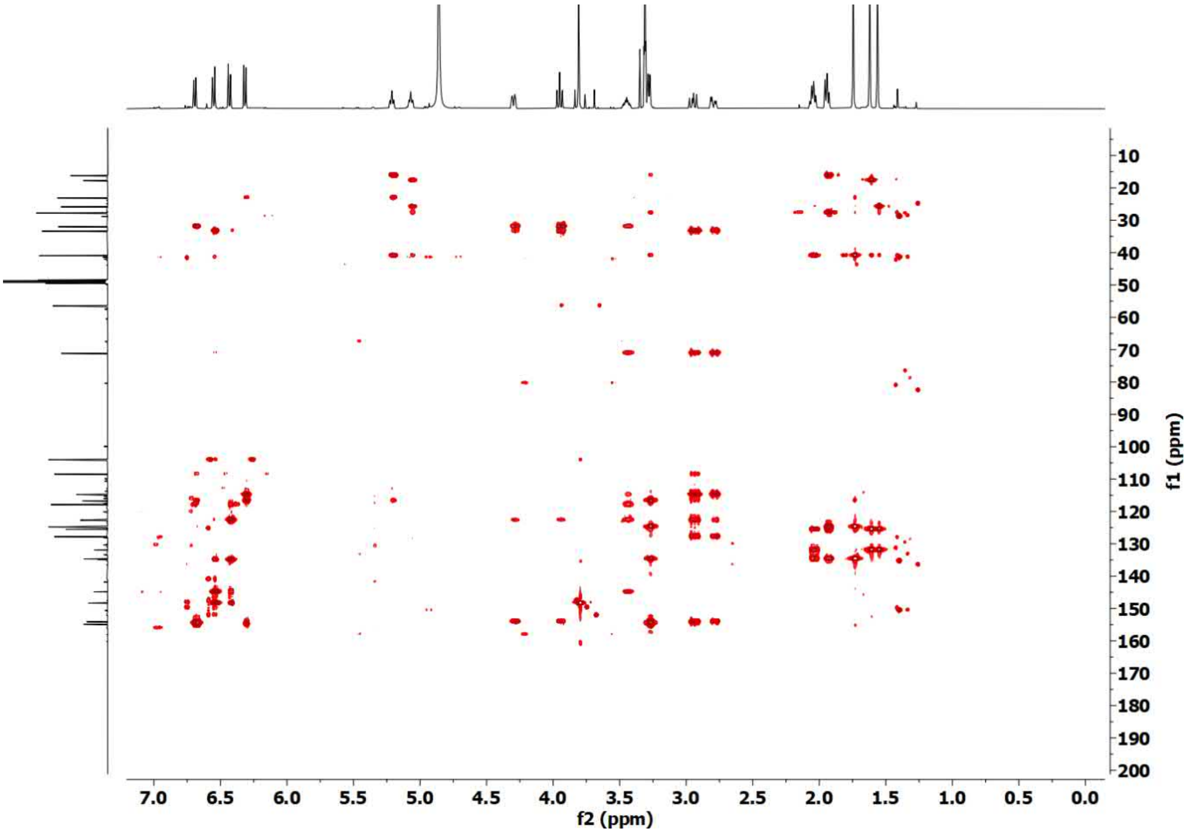

Figure S41. HMBC (500/125 MHz, MeOH-d_4, 25 ºC) spectrum of gloveriflavan A (5)

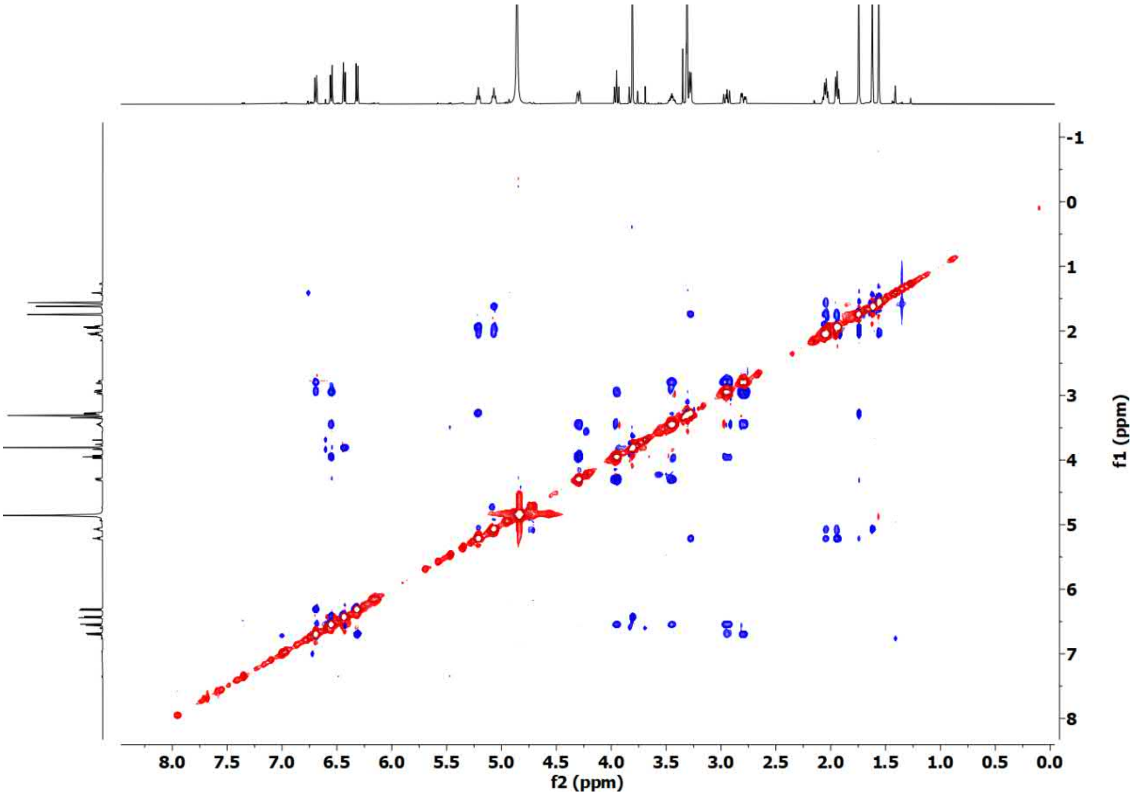

Figure S42. NOESY (500 MHz, MeOH-d_4, 25 ºC) spectrum of gloveriflavan A (**5**)

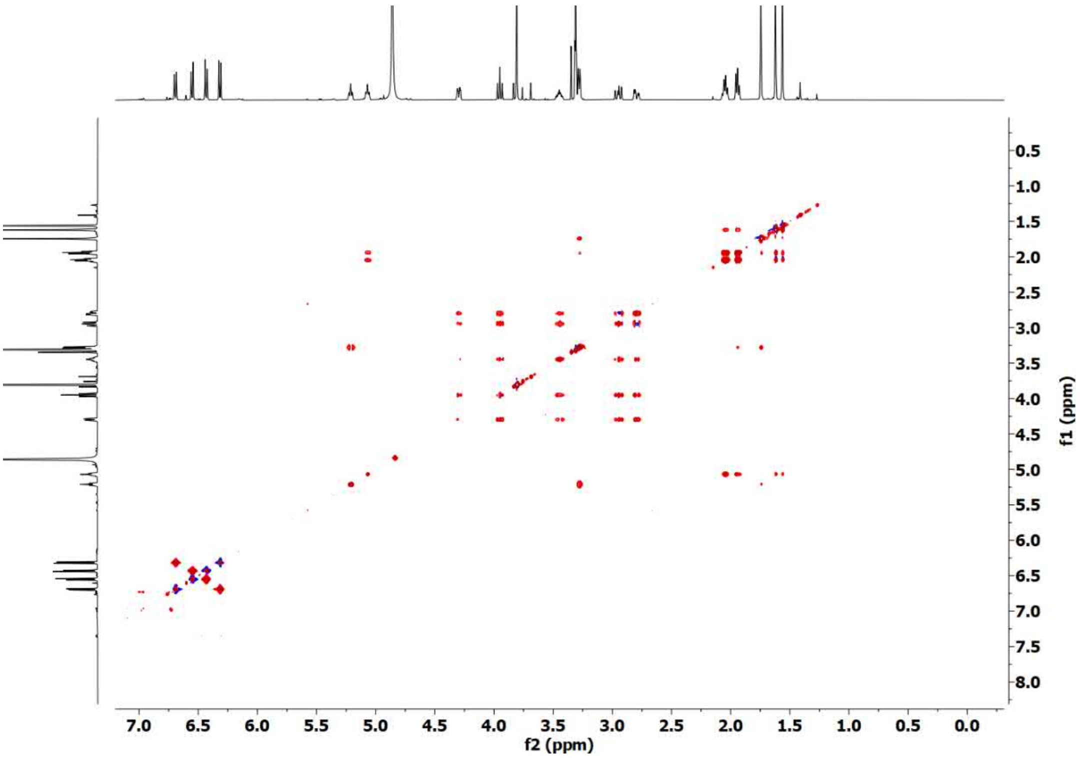

Figure S43. TOCSY (500 MHz, MeOH-d_4, 25 ºC) spectrum of gloveriflavan A (**5**)

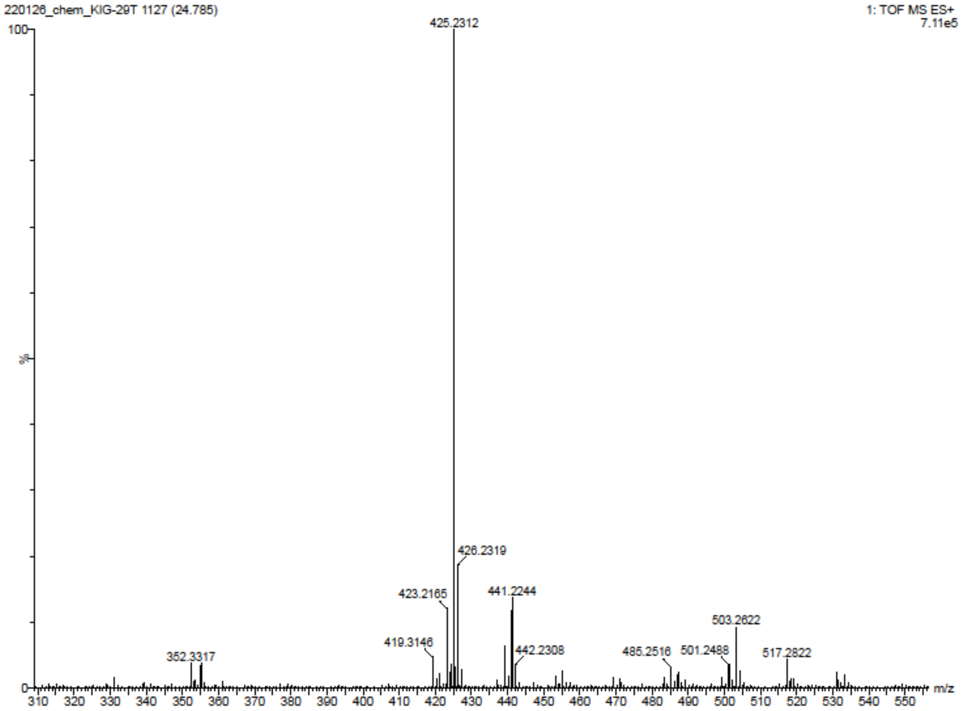

Figure S44. HRESIMS spectrum of gloveriflavan A (**5**)

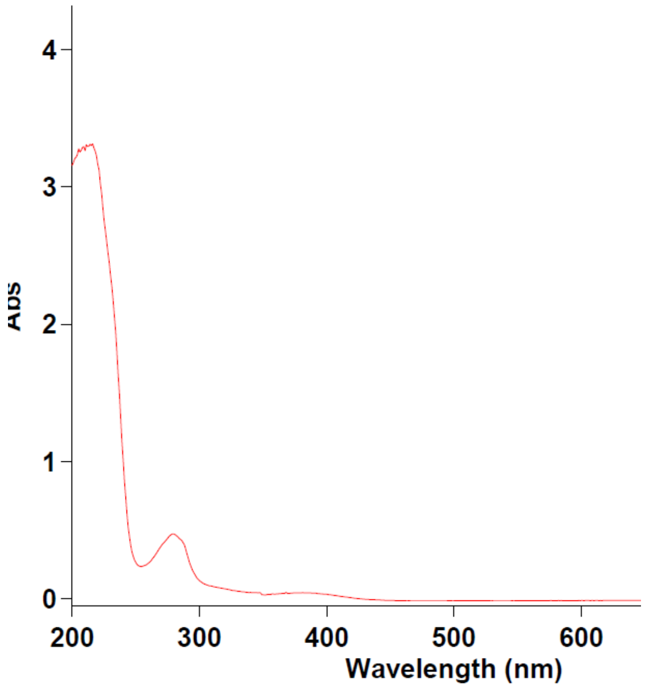

Figure S45. UV spectrum of gloveriflavan A (**5**)

Spectroscopic Data of gloveriflavan B (6)

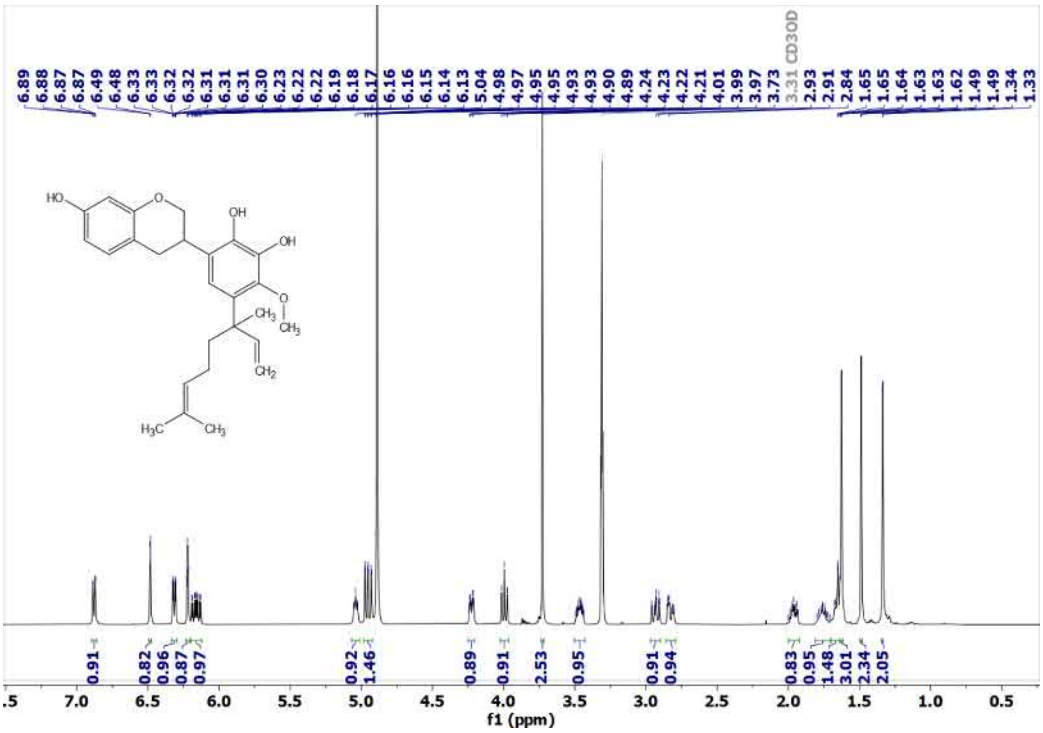

Figure S46. ^{1}H NMR (500 MHz, MeOH-d_4, 25 ºC) spectrum of gloveriflavan B (**6**)

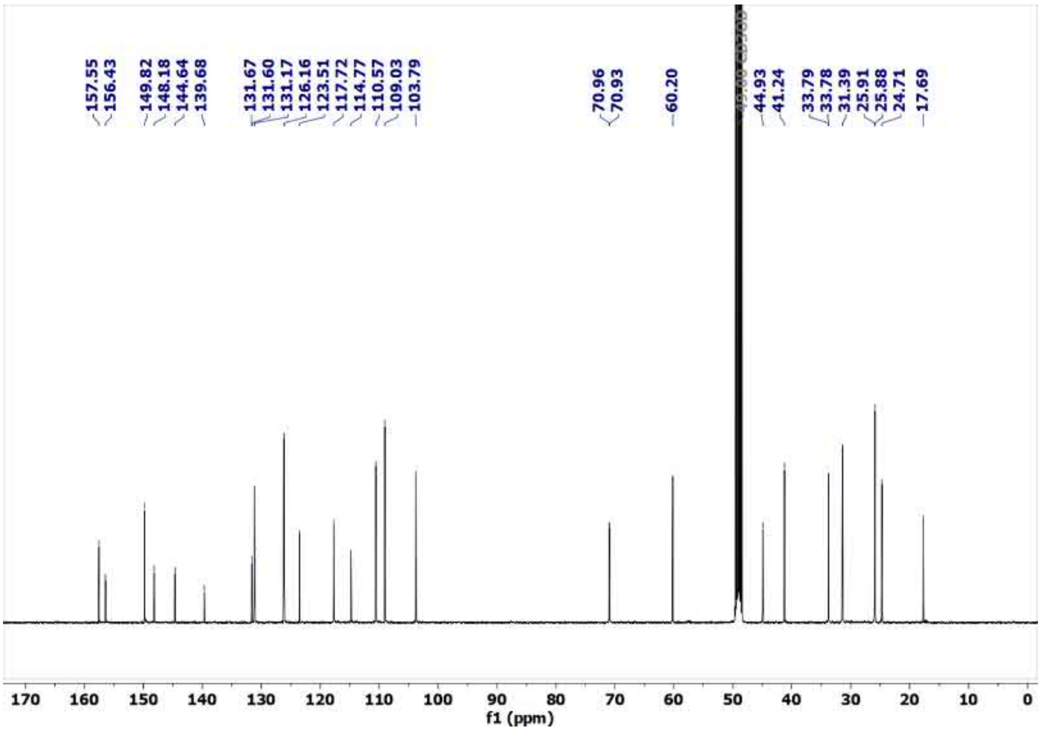

Figure S47. ^{13}C NMR (125 MHz, MeOH-d_4, 25 ºC) spectrum of gloveriflavan B (**6**)

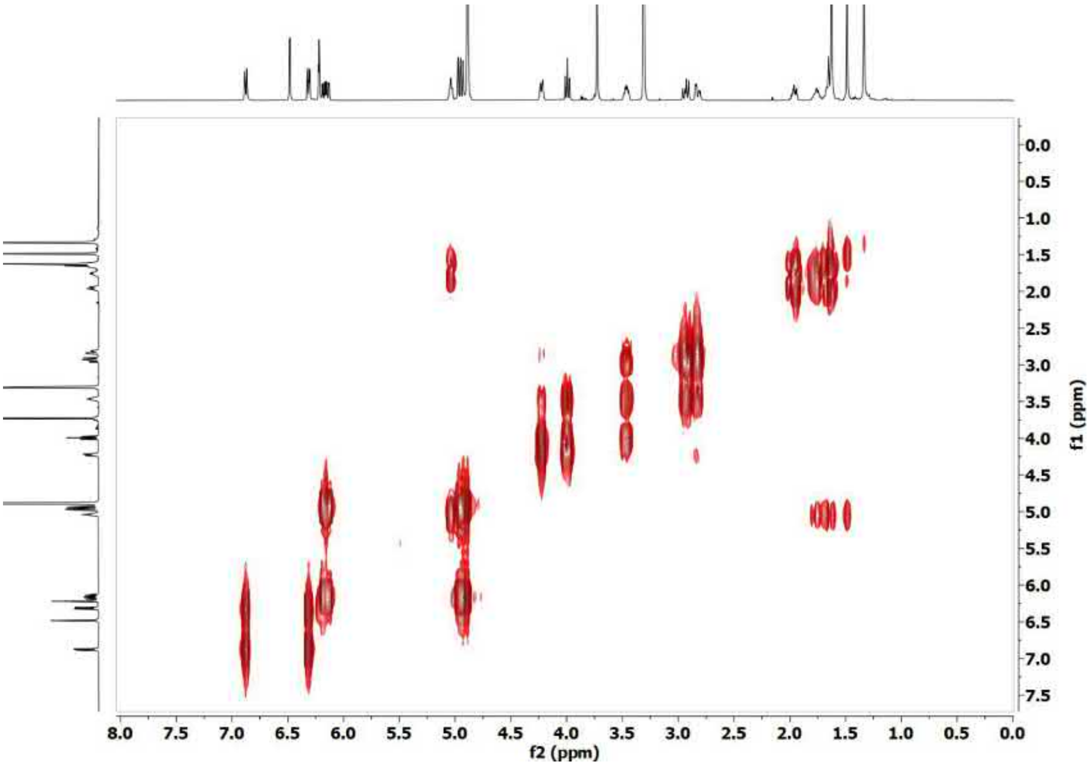

Figure S48. COSY (500 MHz, MeOH-d_4, 25 ºC) spectrum of gloveriflavan B (**6**)

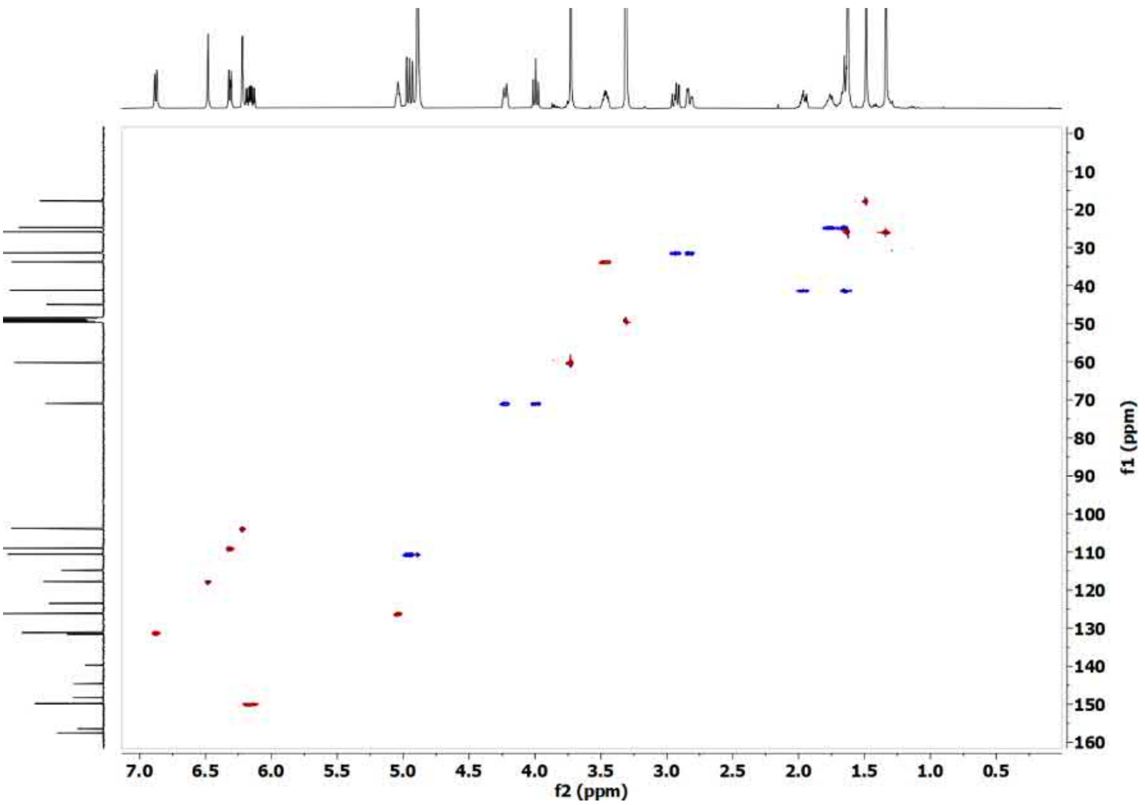

Figure S49. HSQC (500/125 MHz, MeOH-d_4, 25 ºC) spectrum of gloveriflavan B (**6**)

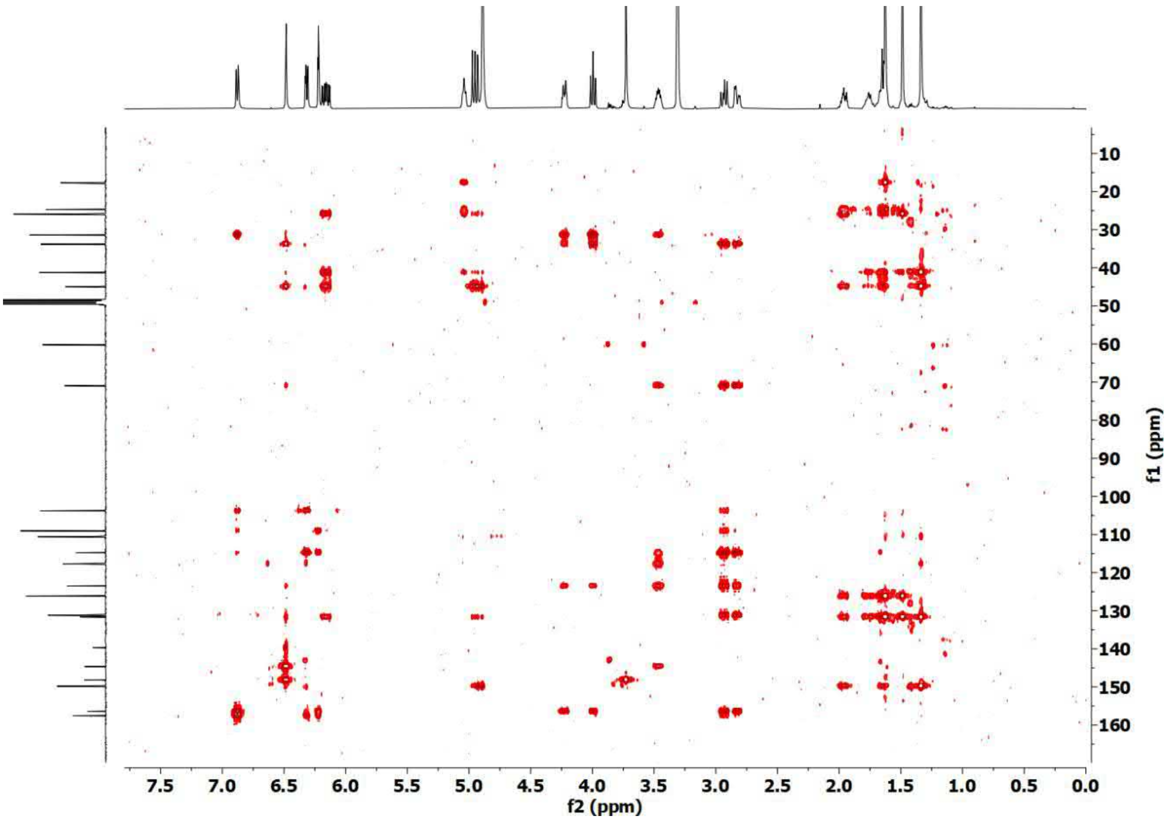

Figure S50. HMBC (500/125 MHz, MeOH-d_4, 25 ºC) spectrum of gloveriflavan B (**6**)

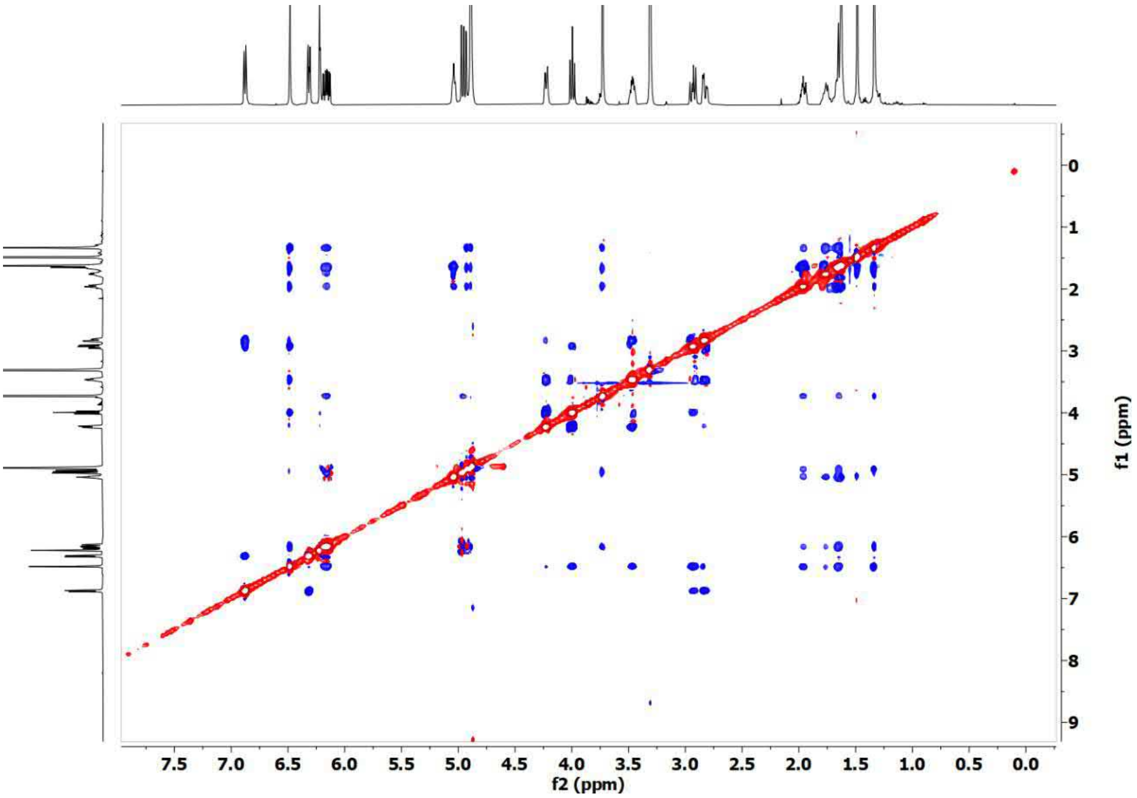

Figure S51. NOESY (500 MHz, MeOH-d_4, 25 ºC) spectrum of gloveriflavan B (**6**)

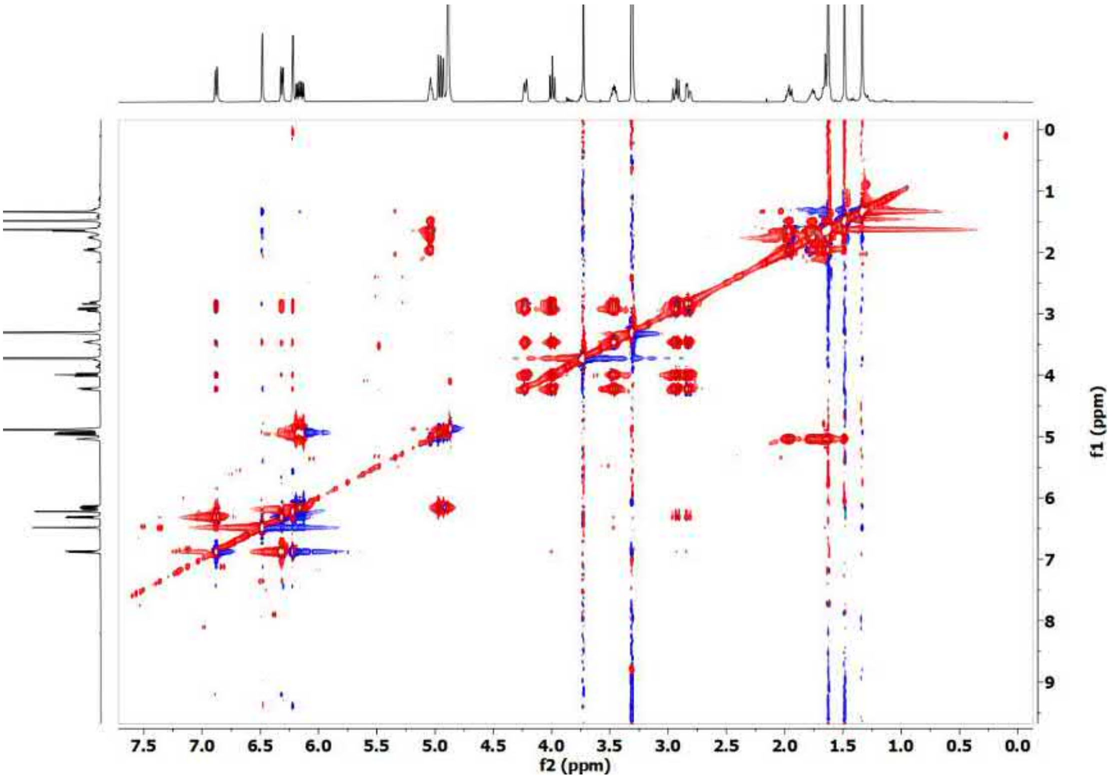

Figure S52. TOCSY (500 MHz, MeOH-d_4, 25 ºC) spectrum of gloveriflavan B (6)

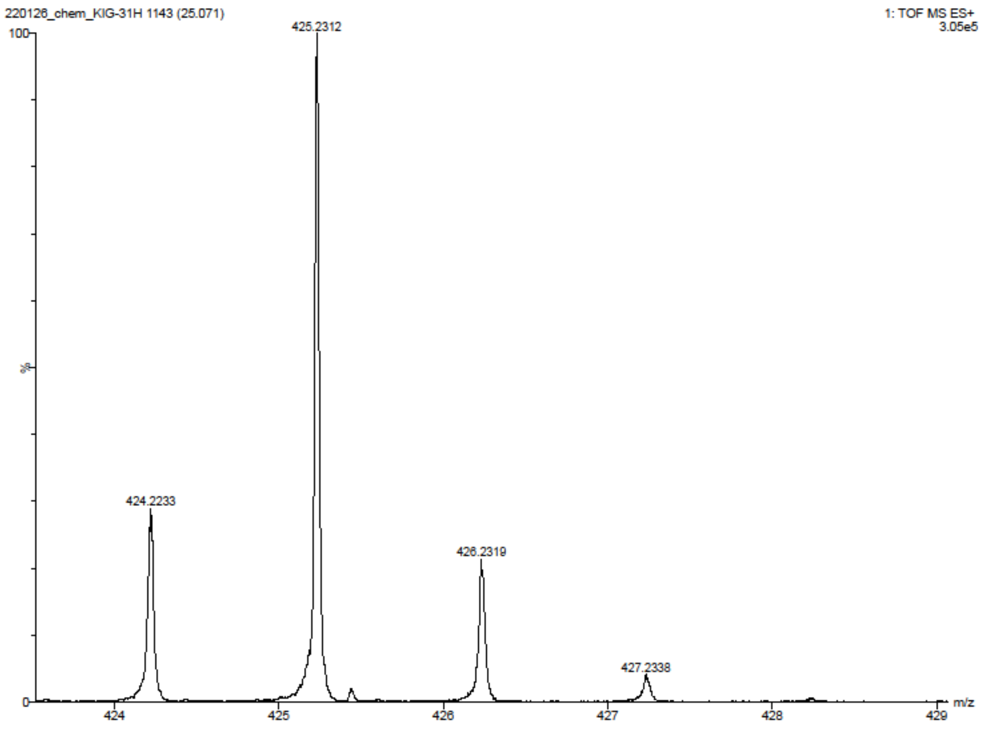

Figure S53. HRESIMS spectrum of gloveriflavan B (**6**)

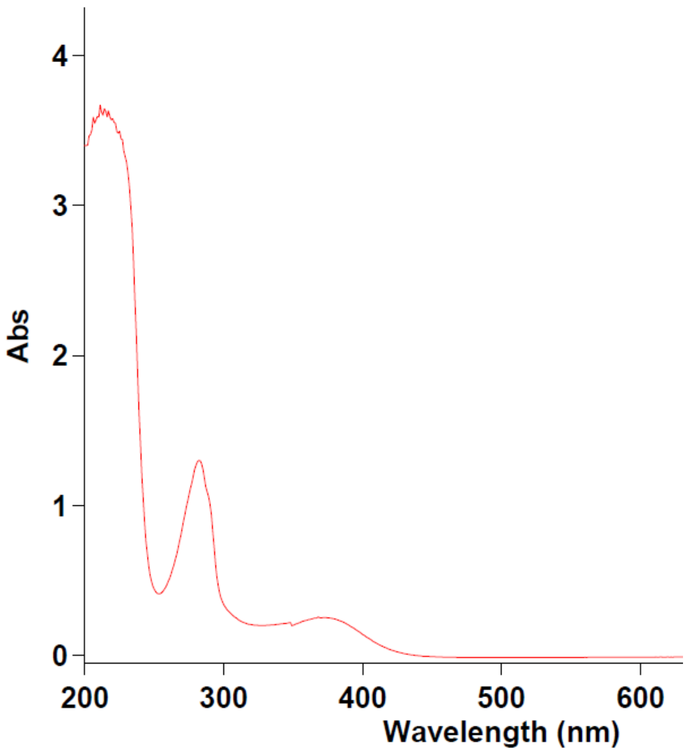

Figure S54. UVspectrum of gloveriflavan B (**6**)

Spectroscopic Data of nitidulin (7)

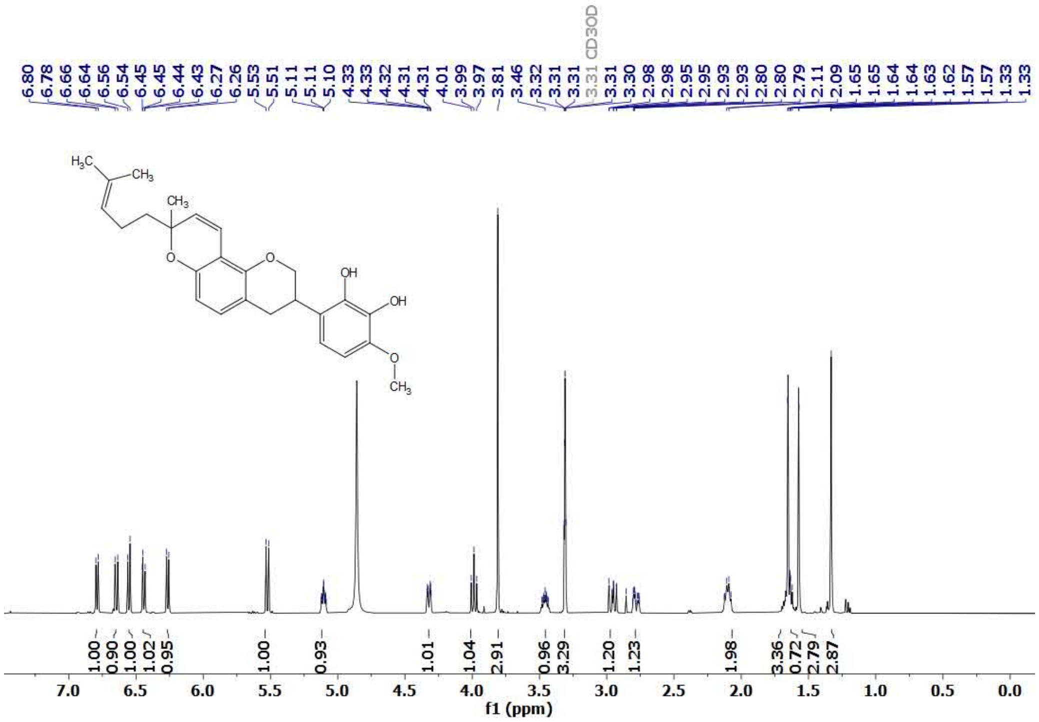

Figure S55. ^{1}H NMR (500 MHz, MeOH-d_4, 25 ºC) spectrum of nitidulin (**7**)

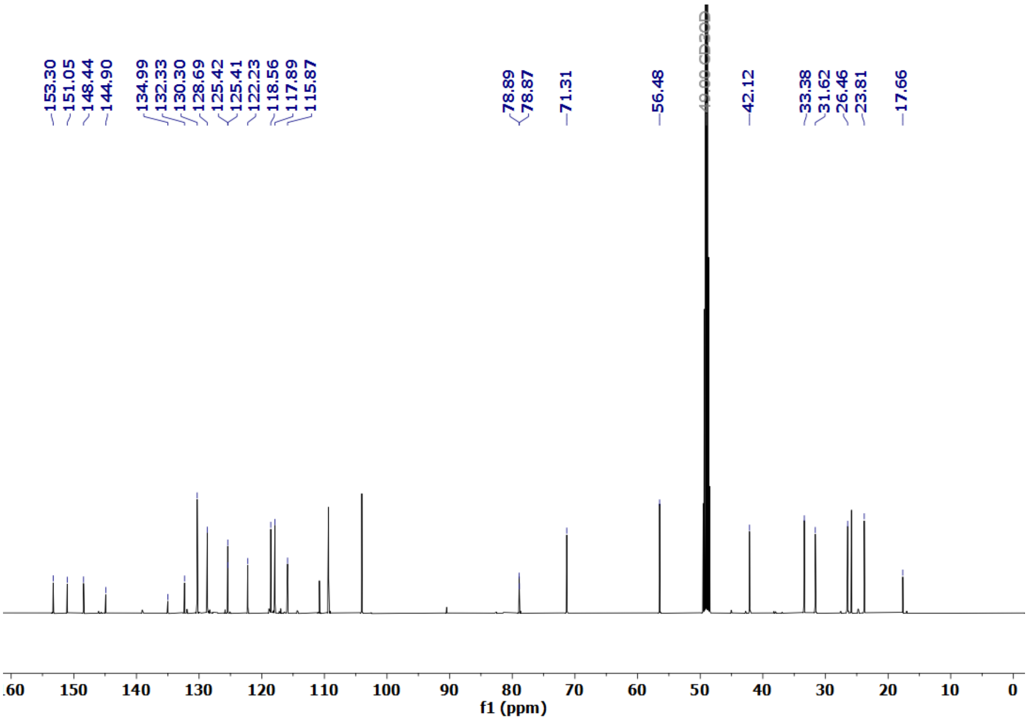

Figure S56. ^{13}C NMR (125 MHz, MeOH-d_4, 25 ºC) spectrum of nitidulin (**7**)

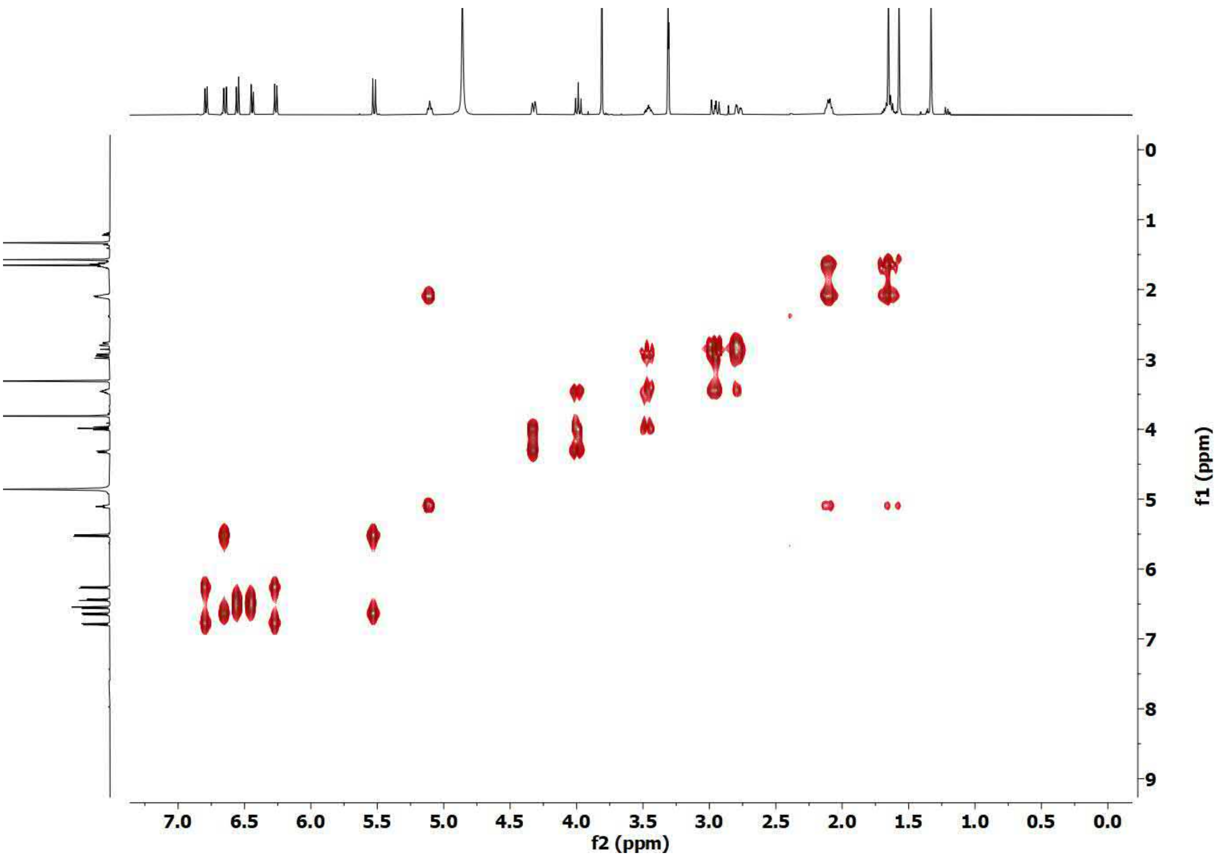

Figure S57. COSY (500 MHz, MeOH-d_4, 25 ºC) spectrum of nitidulin (7)

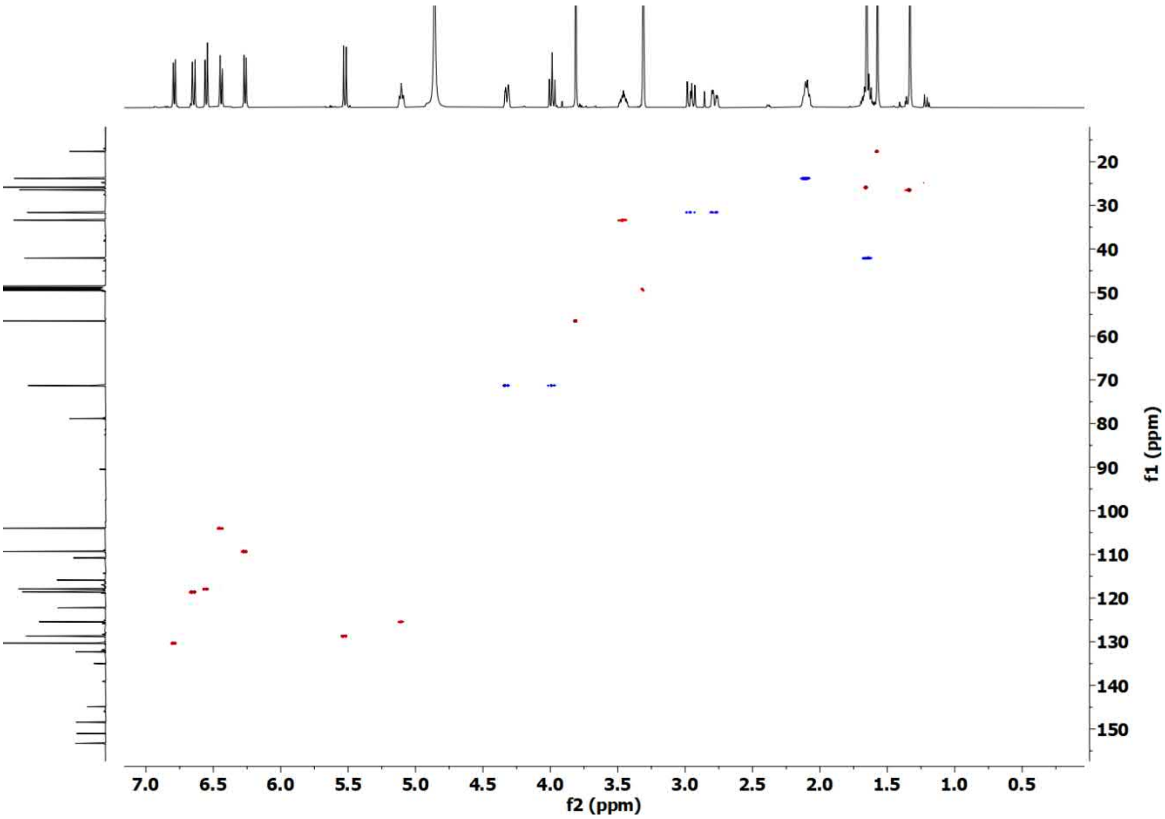

Figure S58. HSQC (500/125 MHz, MeOH-d_4, 25 ºC) spectrum nitidulin (**7**)

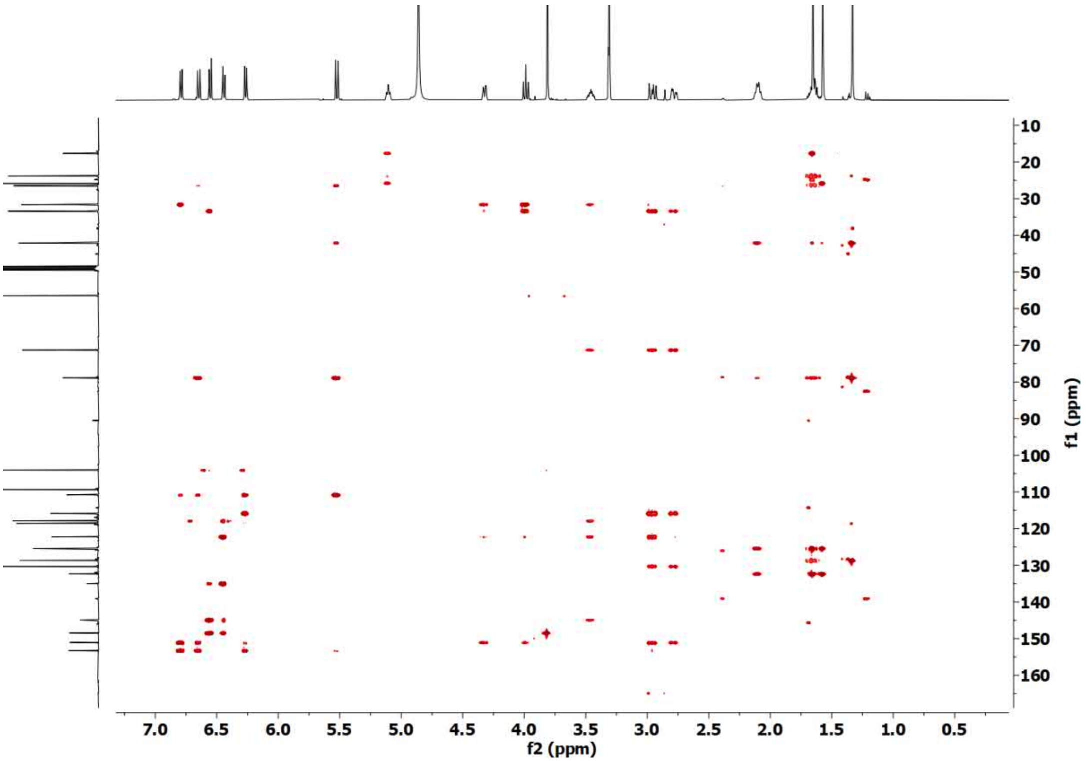

Figure S59. HMBC (500/125 MHz, MeOH-d_4, 25 ºC) spectrum of nitidulin (**7**)

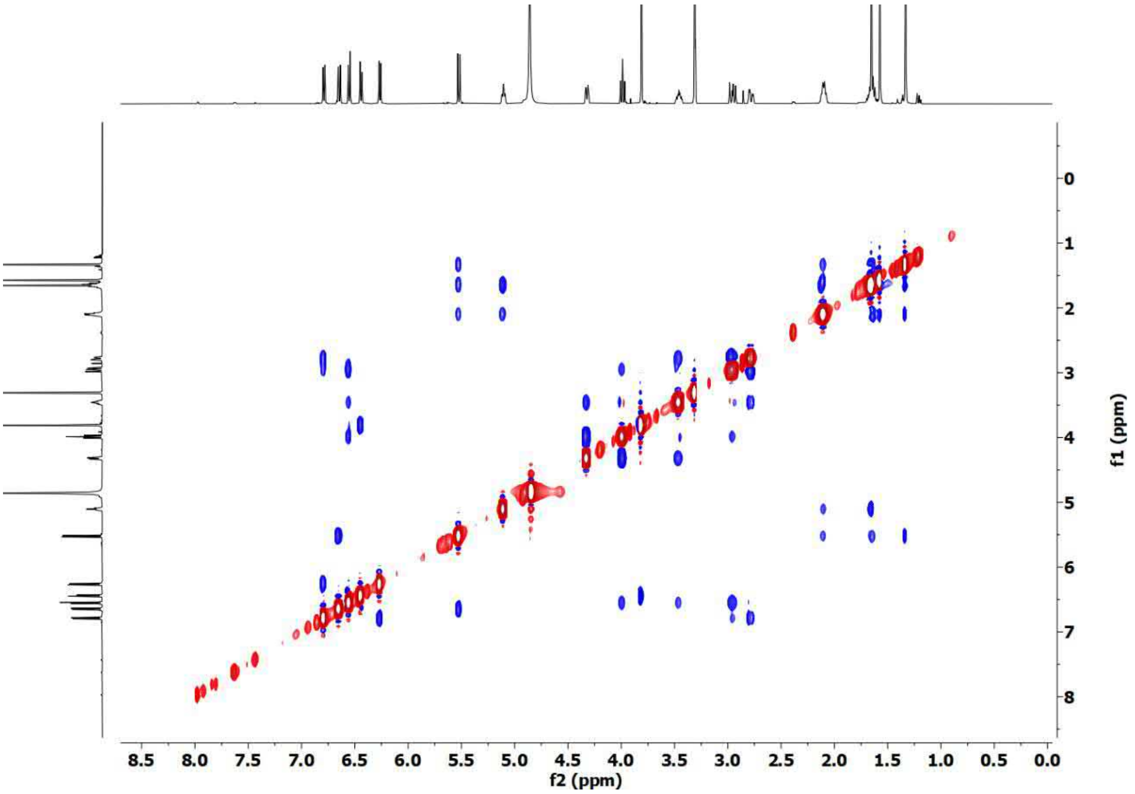

Figure S60. NOESY (500 MHz, MeOH-d_4, 25 ºC) spectrum of nitidulin (**7**)

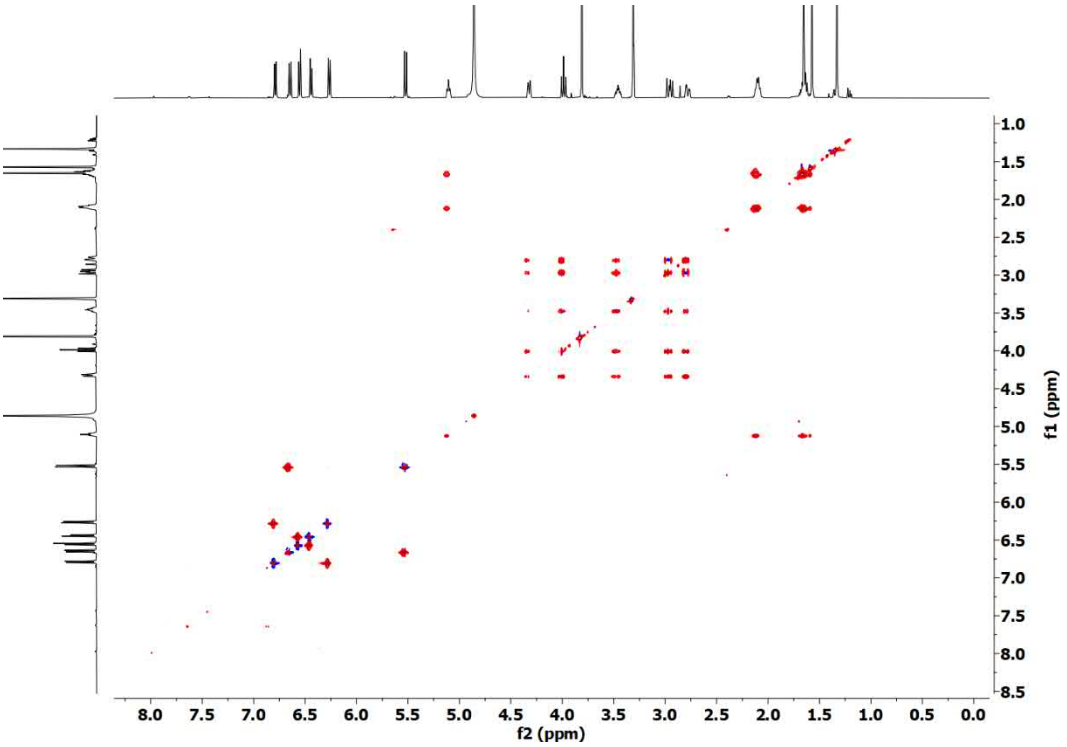

Figure S61. TOCSY (500 MHz, MeOH-d_4, 25 ºC) spectrum of gloveriflavan B (**6**)

Spectroscopic Data of lespeol (8)

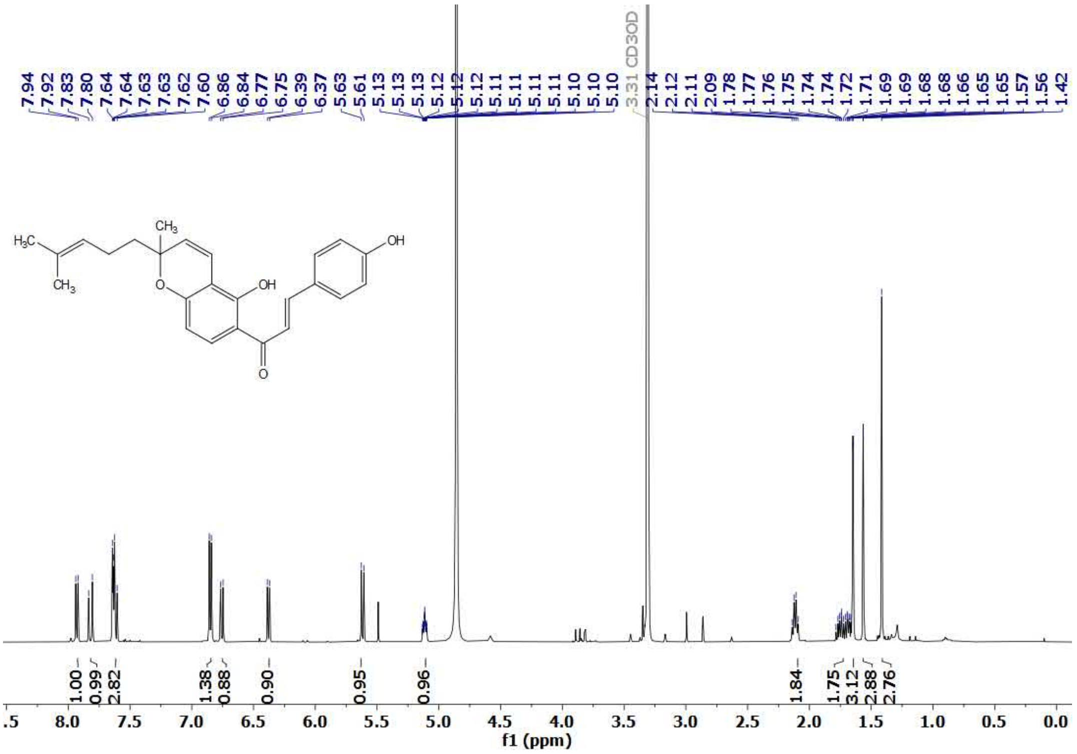

Figure S62. ^{1}H NMR (500 MHz, MeOH-d_4, 25 ºC) spectrum of lespeol (**8**)

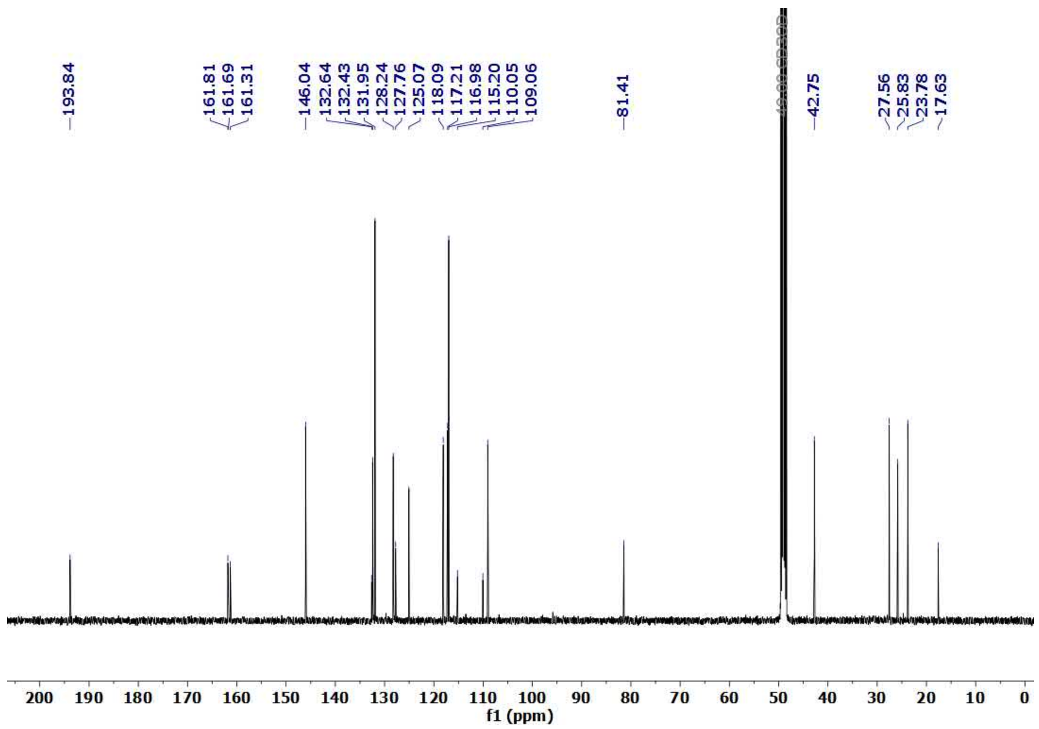

Figure S63. ^{13}C NMR (125 MHz, MeOH-d_4, 25 ºC) spectrum of lespeol (**8**)

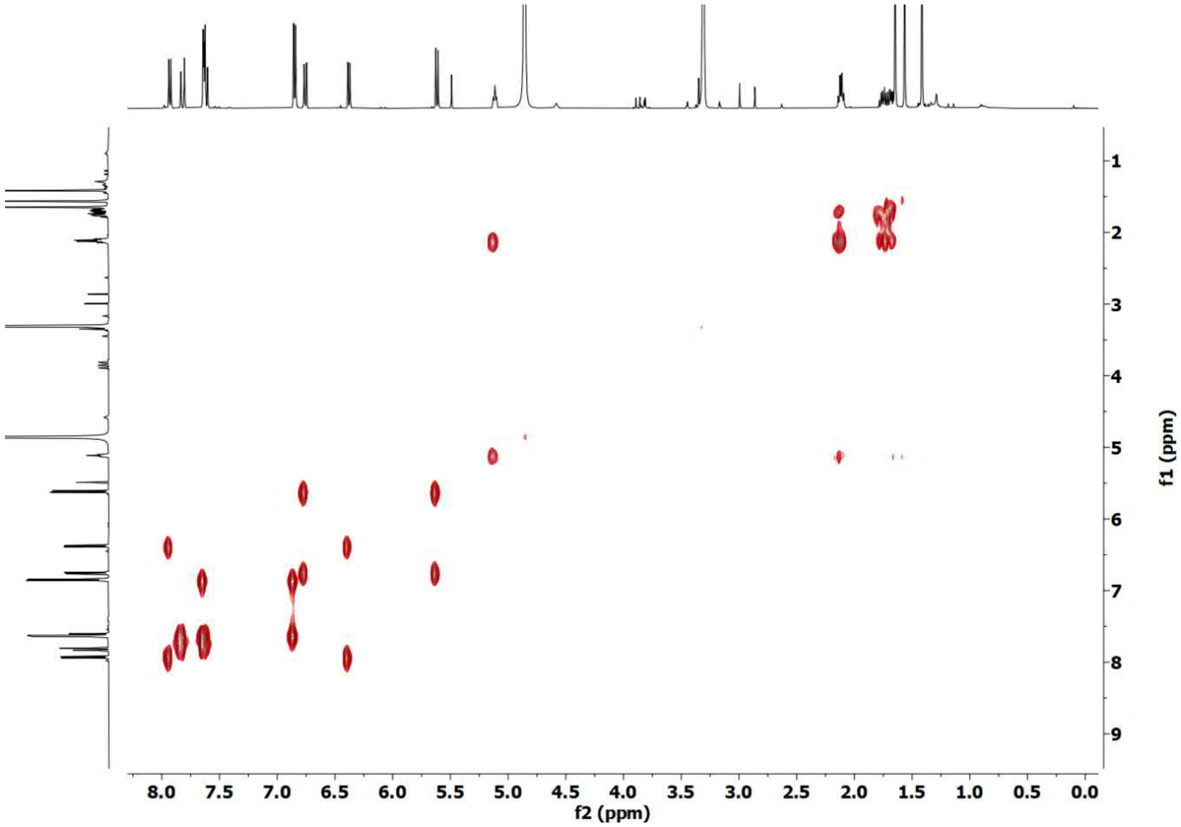

Figure S64. COSY (500 MHz, MeOH-d_4, 25 ºC) spectrum of lespeol (**8**)

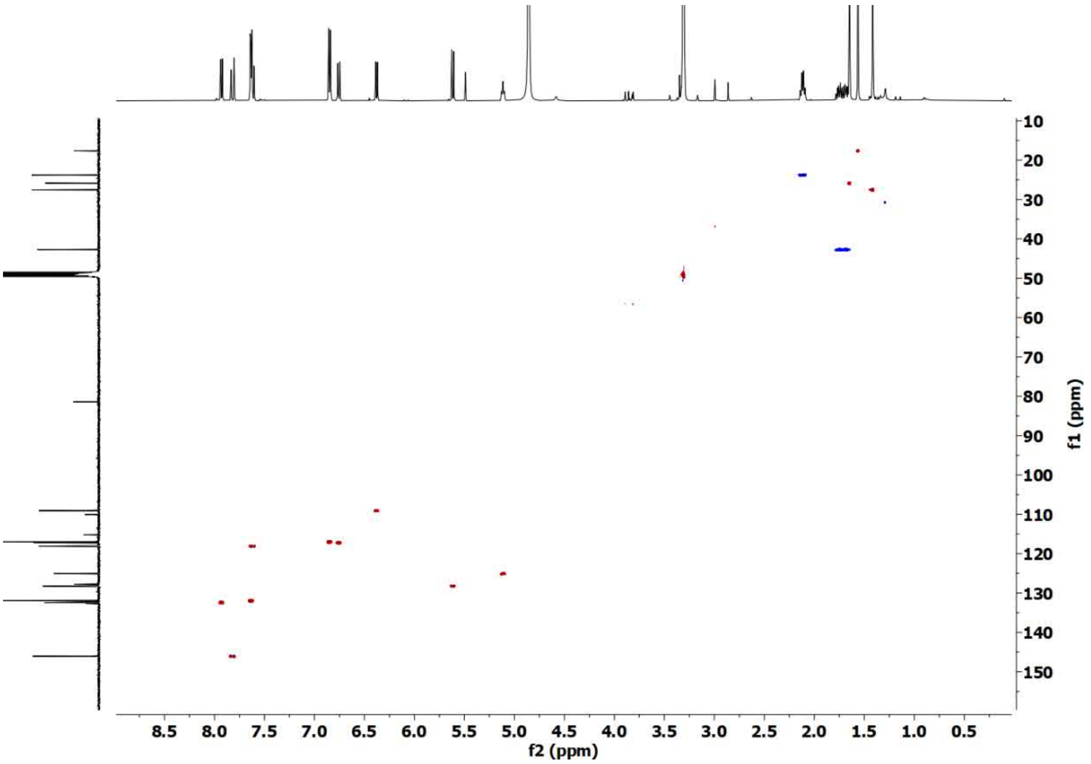

Figure S65. HSQC (500/125 MHz, MeOH-d_4, 25 ºC) spectrum of lespeol (**8**)

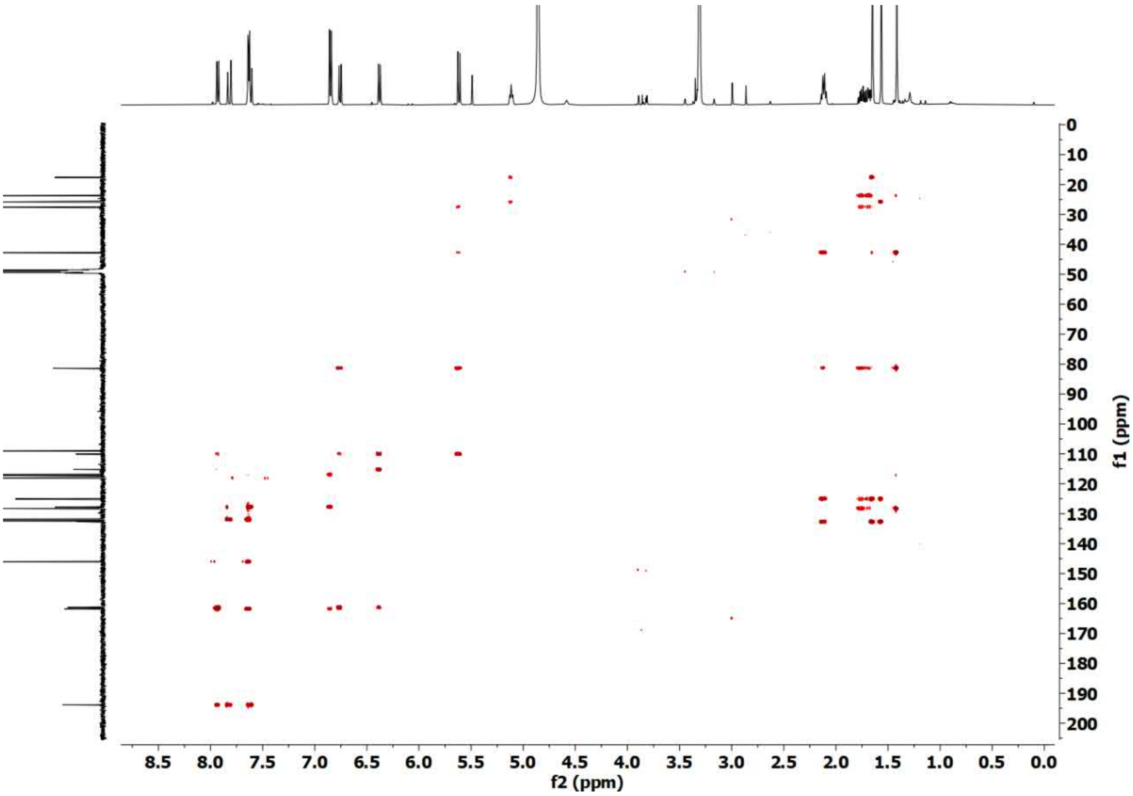

Figure S66. HMBC (500/125 MHz, MeOH-d_4, 25 ºC) spectrum of lespeol (**8**)

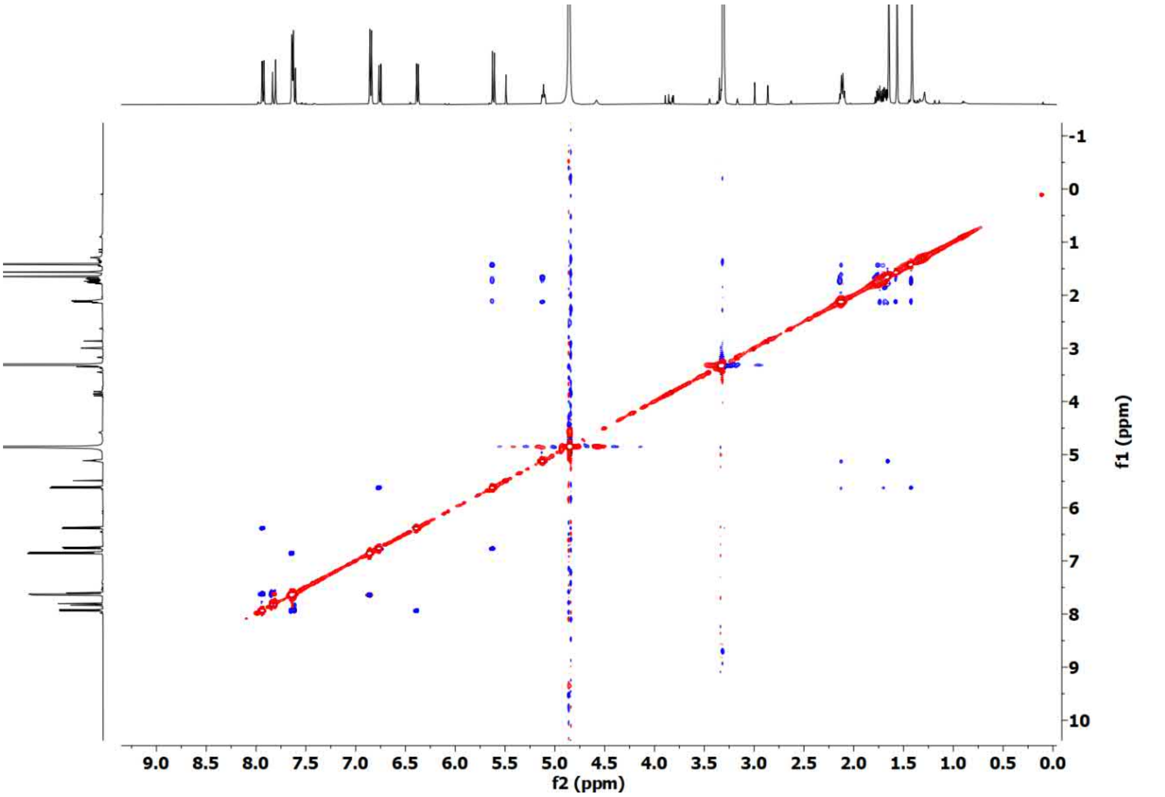

Figure S67. NOESY (500 MHz, MeOH-d_4, 25 ºC) spectrum of lespeol (**8**)

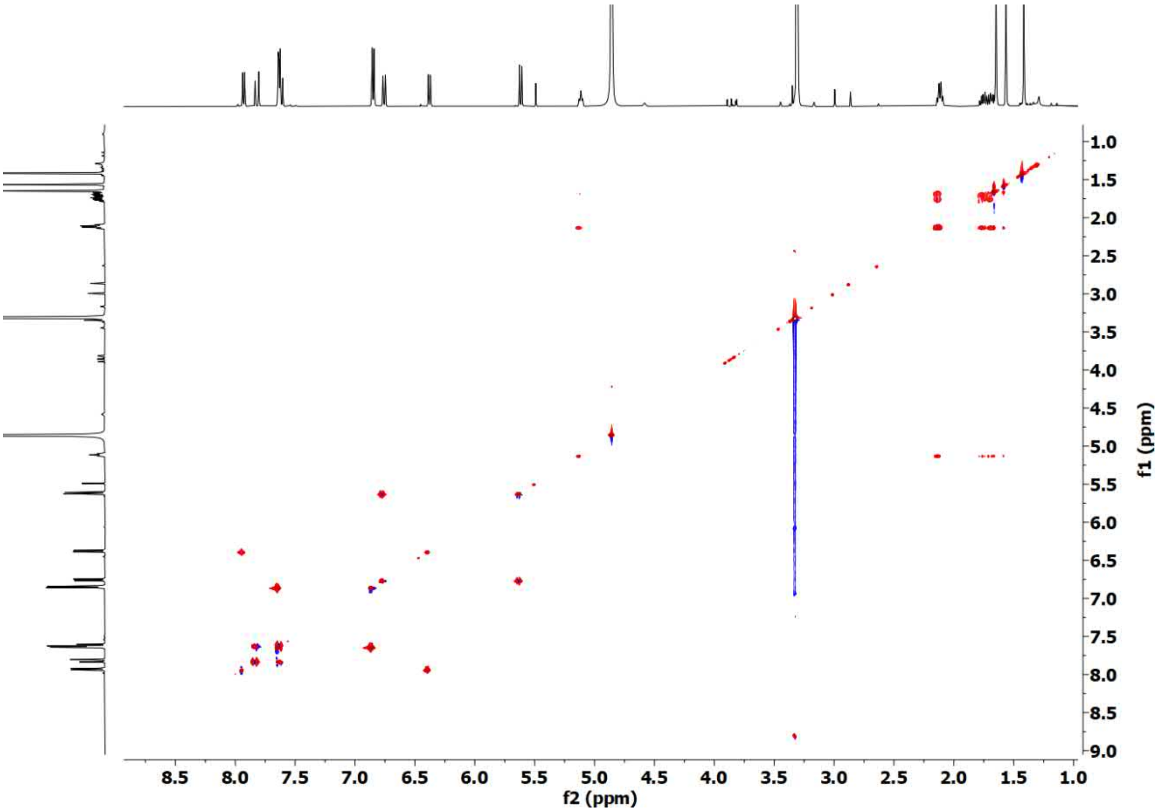

Figure S68. TOCSY (500 MHz, MeOH-d_4, 25 ºC) spectrum of lespeol (**8**)

Spectroscopic Data of isoliquiritigenin (9)

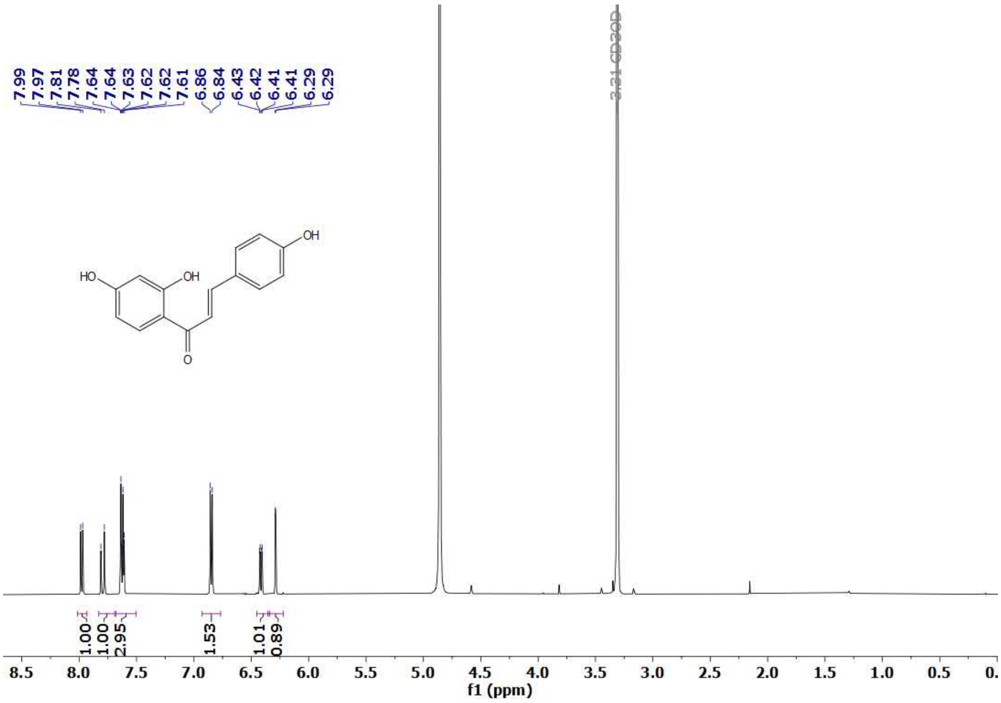

Figure S69. ^{1}H NMR (500 MHz, MeOH-d_4, 25 ºC) spectrum of isoliquiritigenin (**9**)

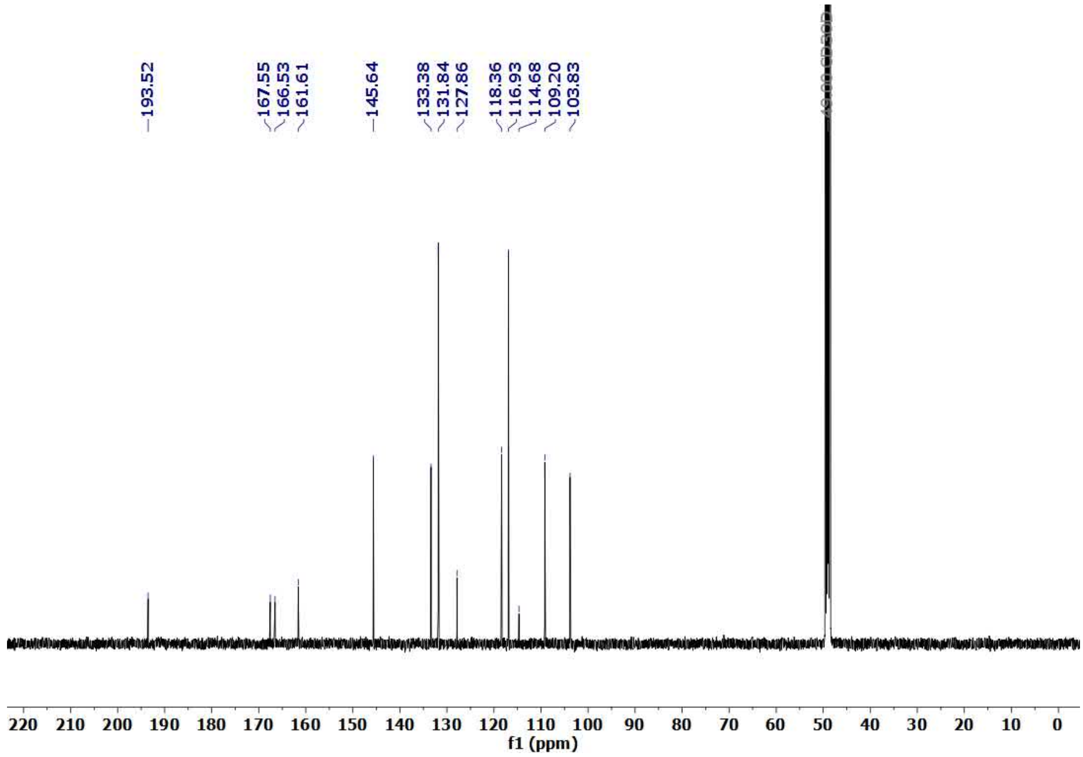

Figure S70. ^{13}C NMR (125 MHz, MeOH-d_4, 25 ºC) spectrum of isoliquiritigenin (**9**)

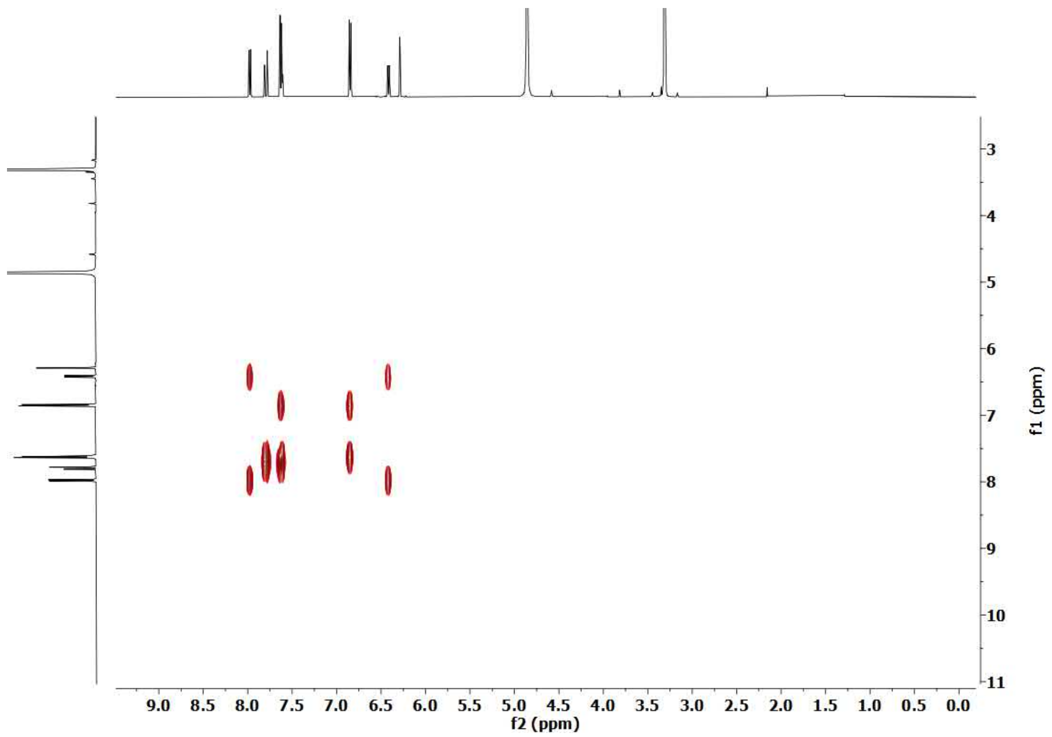

Figure S71. COSY (500 MHz, MeOH-d_4, 25 ºC) spectrum of isoliquiritigenin (**9**)

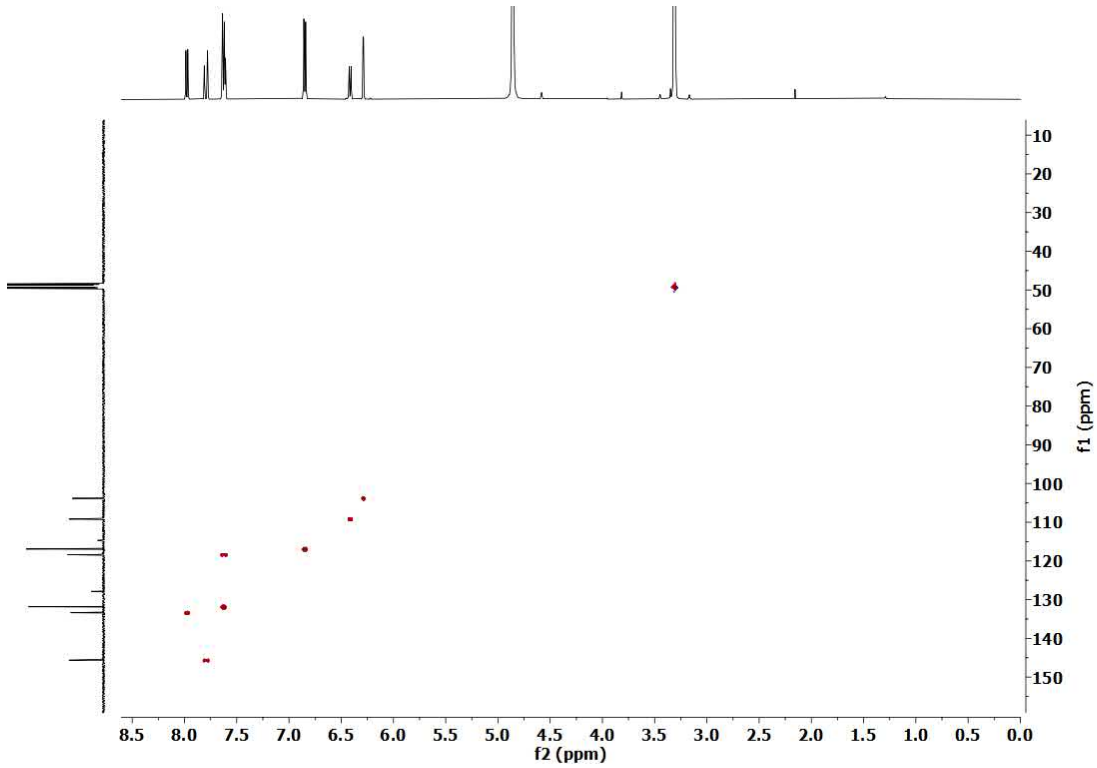

Figure S71. HSQC (500/125 MHz, MeOH-d_4, 25 ºC) spectrum of isoliquiritigenin (**9**)

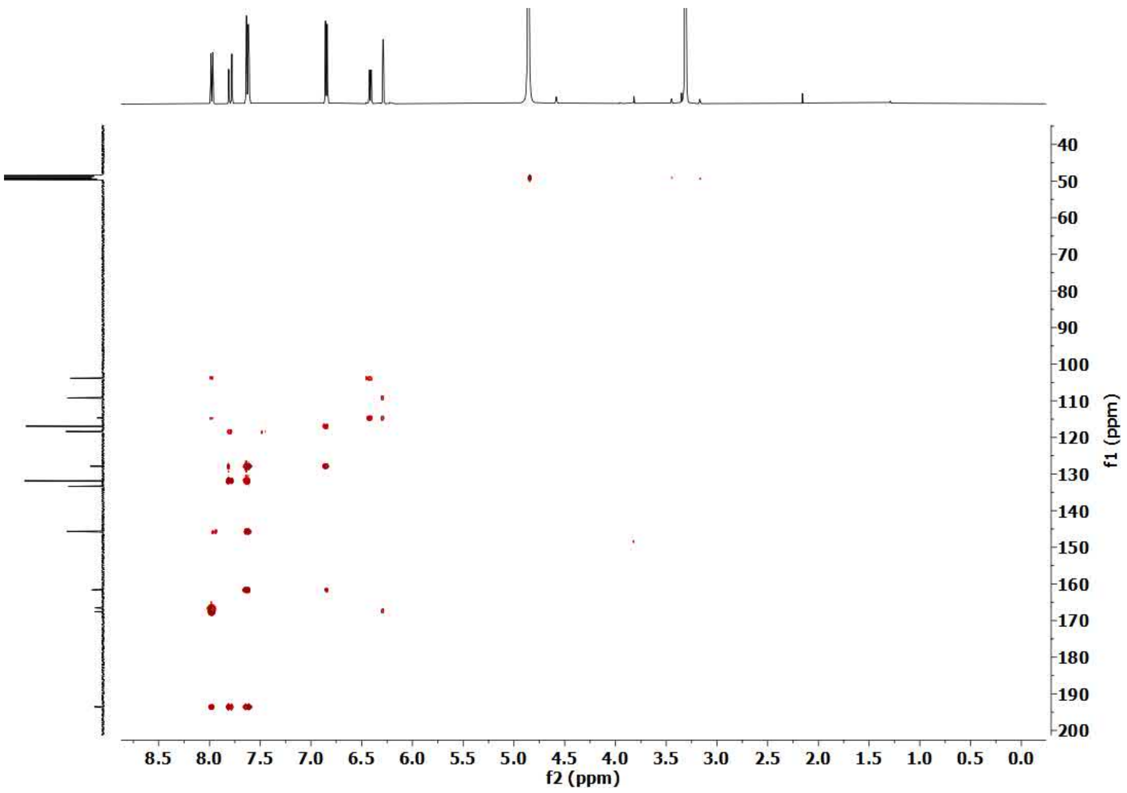

Figure S72. HMBC (500/125 MHz, MeOH-d_4, 25 ºC) spectrum of isoliquiritigenin (**9**)

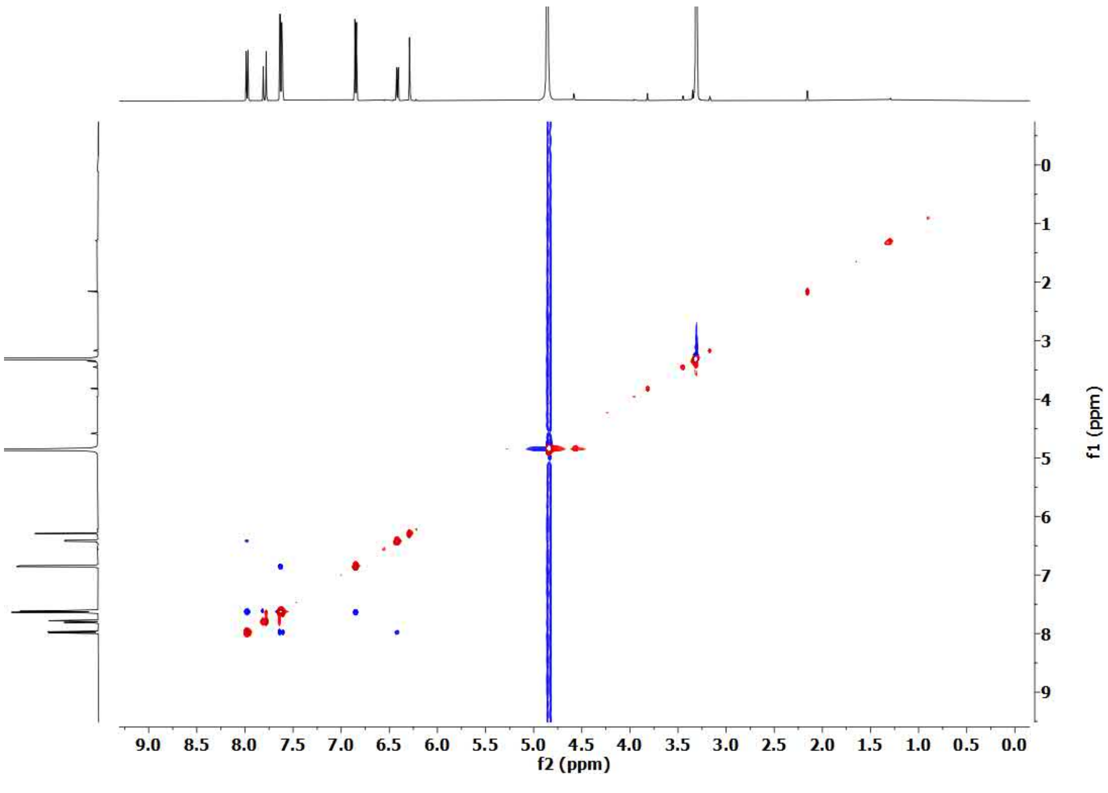

Figure S73. NOESY (500 MHz, MeOH-d_4, 25 ºC) spectrum of isoliquiritigenin (**9**)

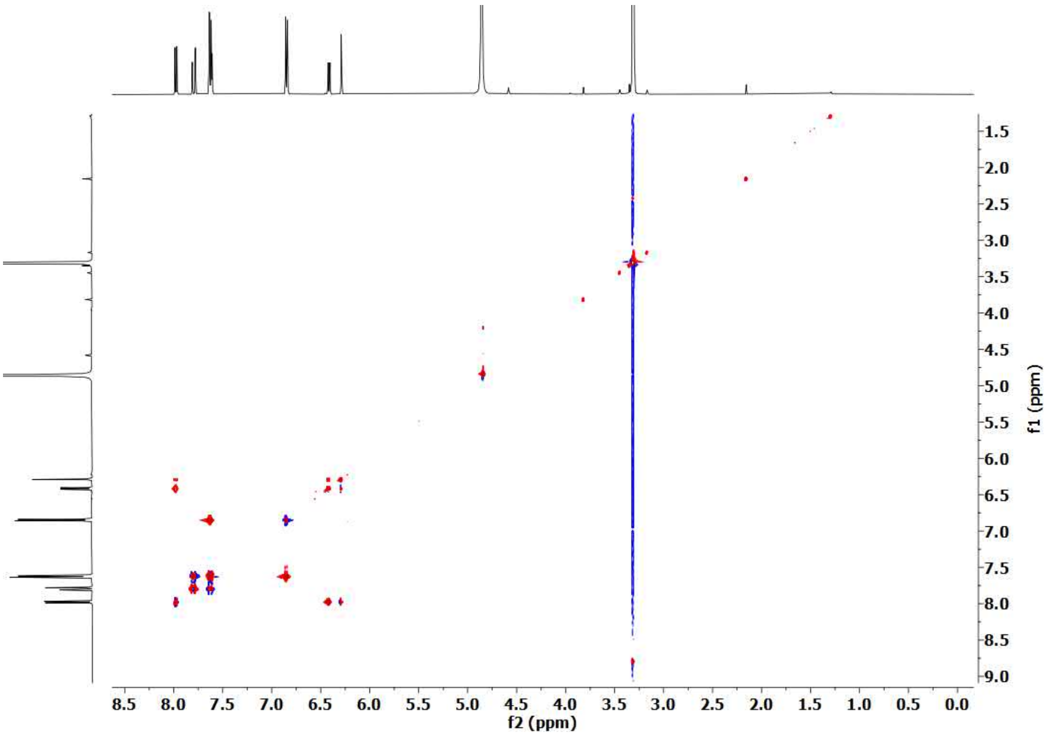

Figure S74. TOCSY (500 MHz, MeOH-d_4, 25 ºC) spectrum of isoliquiritigenin (**9**)

Spectroscopic Data of 1-(2,4-dihydroxyphenyl)-3-hydroxy-3-(4-hydroxyphenyl)-1-propanone (10)

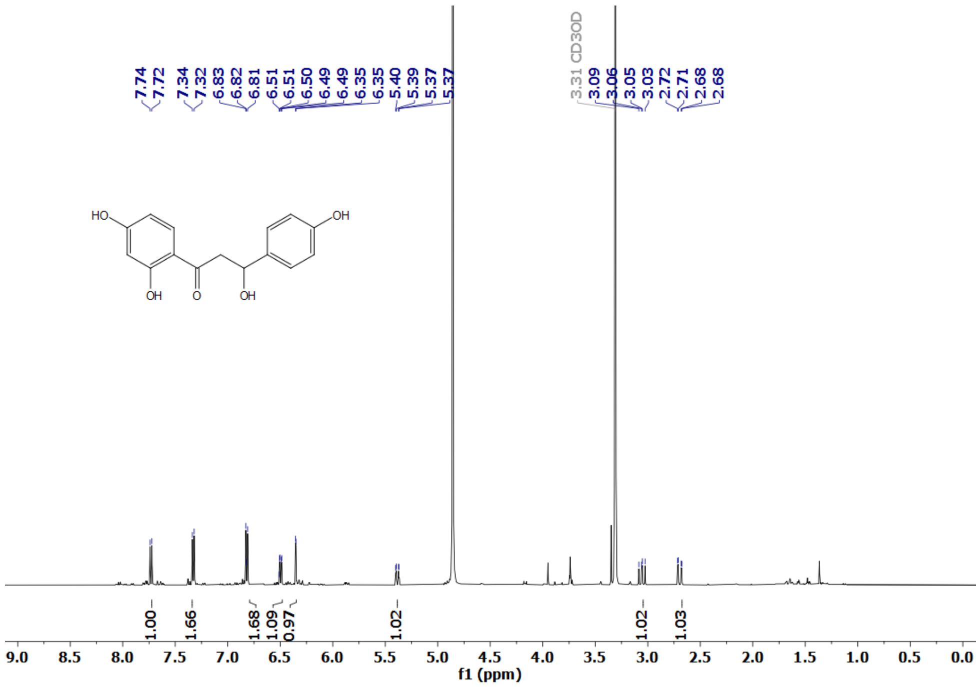

Figure S75. ^{1}H NMR (500 MHz, MeOH-d_4, 25 ºC) spectrum of 1-(2,4-dihydroxyphenyl)-3-hydroxy-3-(4-hydroxyphenyl)-1-propanone (**10**).

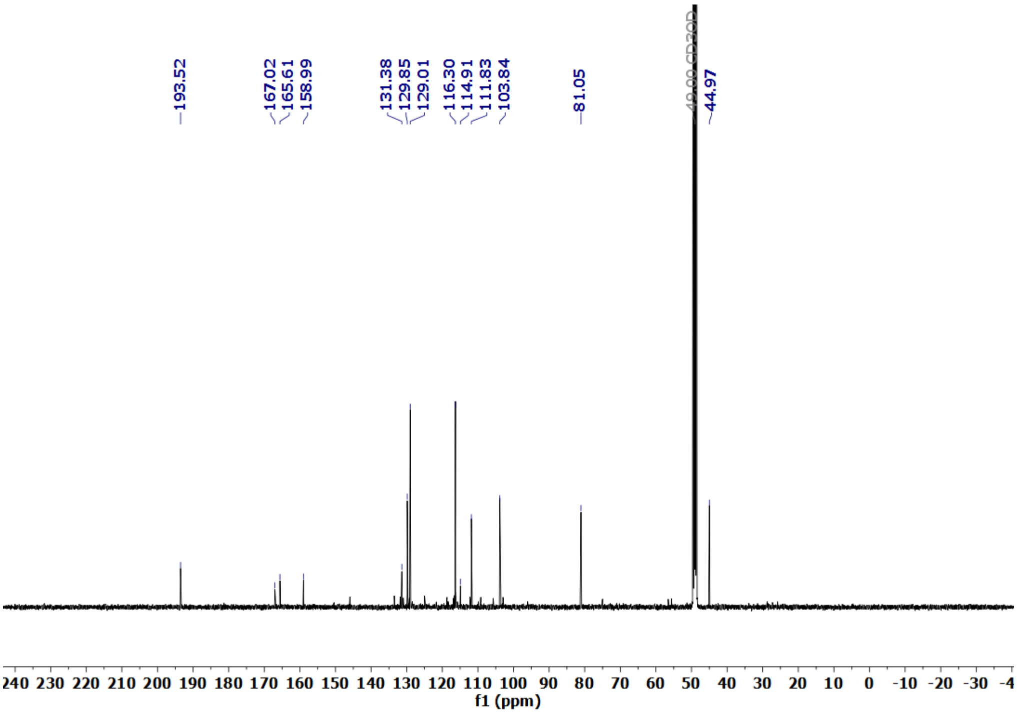

Figure S76. ^{13}C NMR (125 MHz, MeOH-d_4, 25 ºC) spectrum of 1-(2,4-dihydroxyphenyl)-3-hydroxy-3-(4-hydroxyphenyl)-1-propanone (**10**).

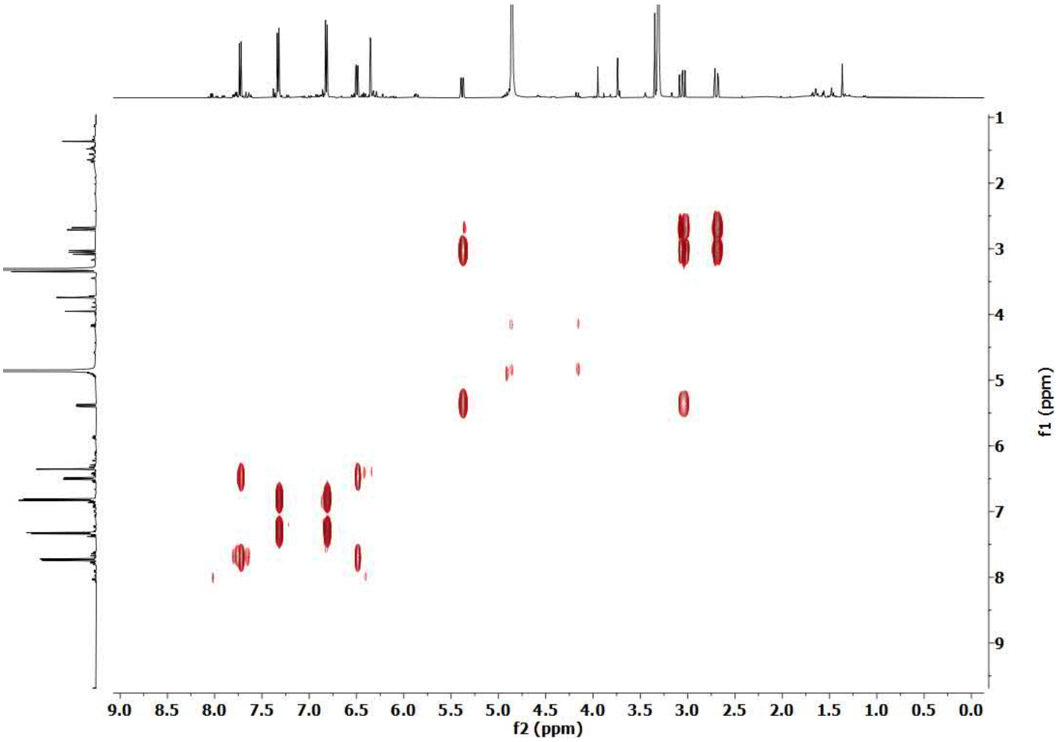

Figure S77. COSY (500 MHz, MeOH-d_4, 25 ºC) spectrum of 1-(2,4-dihydroxyphenyl)-3-hydroxy-3-(4-hydroxyphenyl)-1-propanone (**10**)

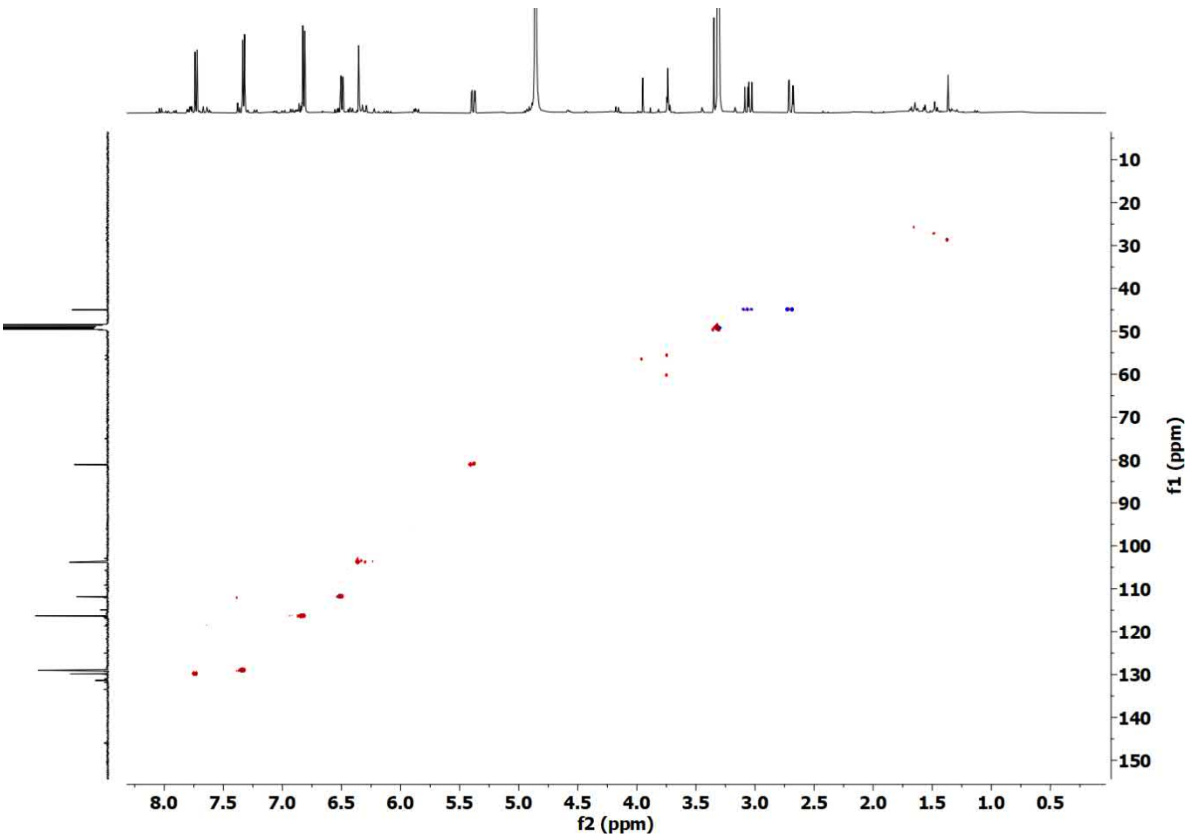

Figure S77. HSQC (500/125 MHz, MeOH-d_4, 25 ºC) spectrum of 1-(2,4-dihydroxyphenyl)-3-hydroxy-3-(4-hydroxyphenyl)-1-propanone (**10**).

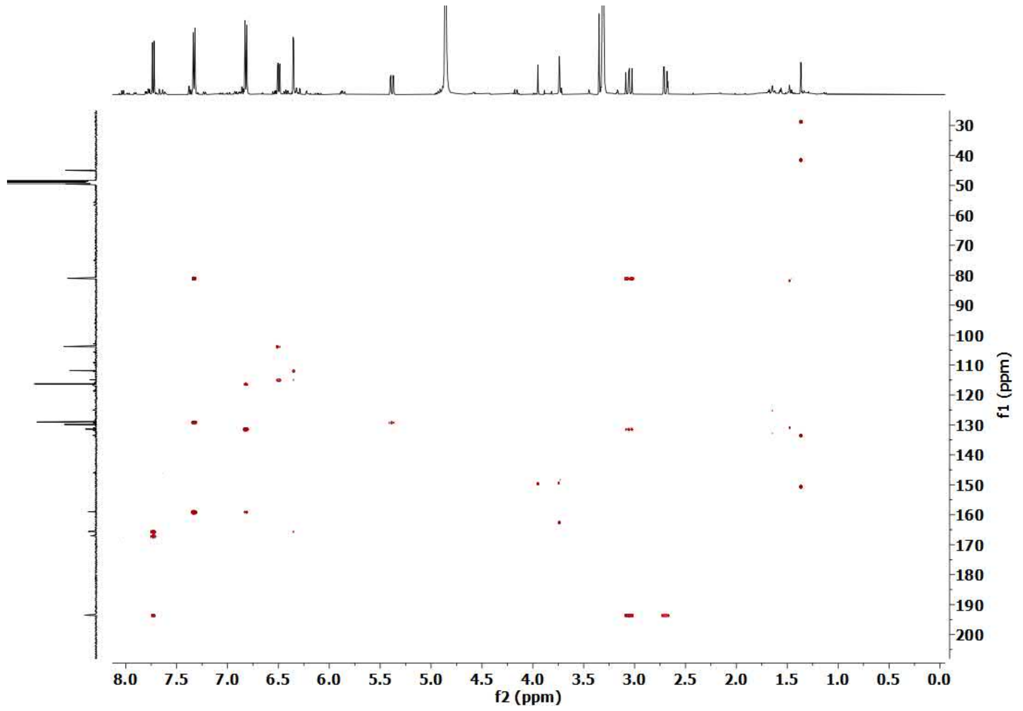

Figure S78. HMBC (500/125 MHz, MeOH-d_4, 25 ºC) spectrum of 1-(2,4-dihydroxyphenyl)-3-hydroxy-3-(4-hydroxyphenyl)-1-propanone (**10**)

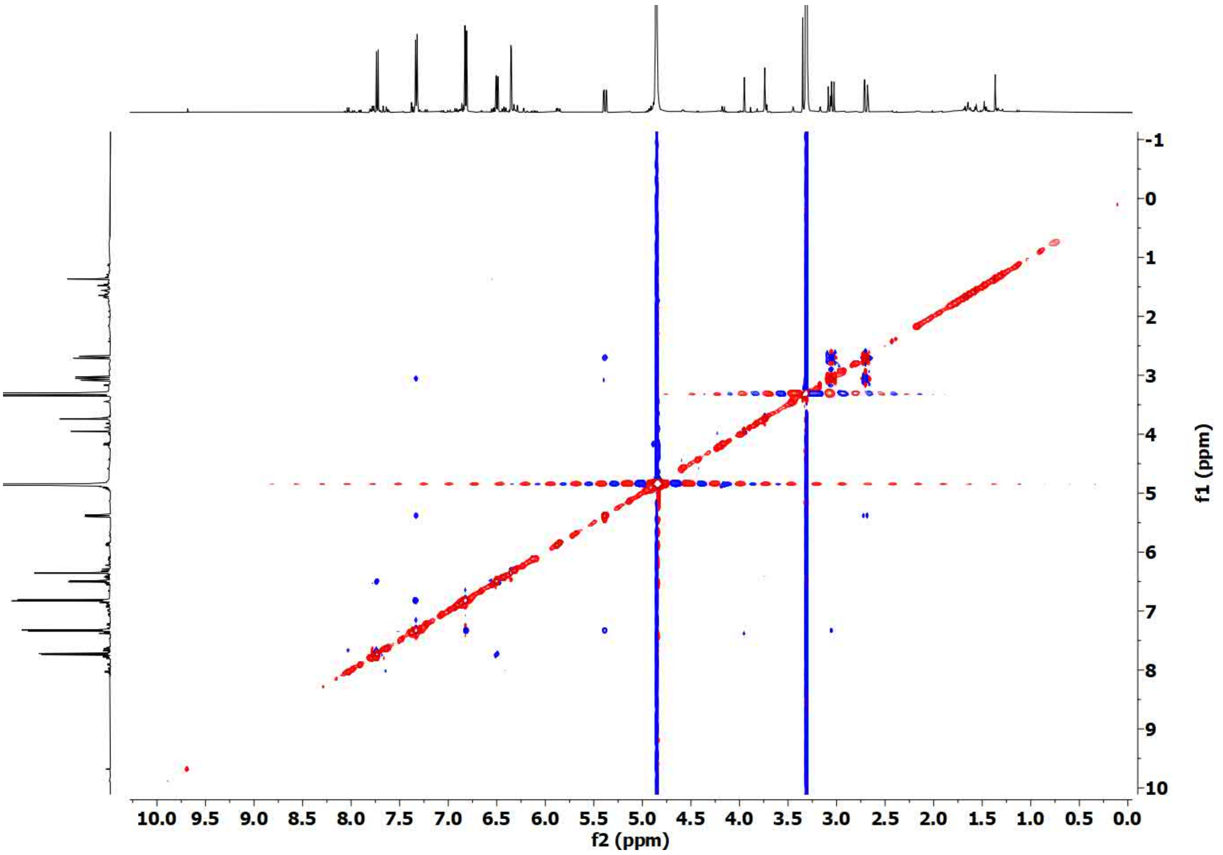

Figure S79. NOESY (500 MHz, MeOH-d_4, 25 ºC) spectrum of 1-(2,4-dihydroxyphenyl)-3-hydroxy-3-(4-hydroxyphenyl)-1-propanone (**10**)

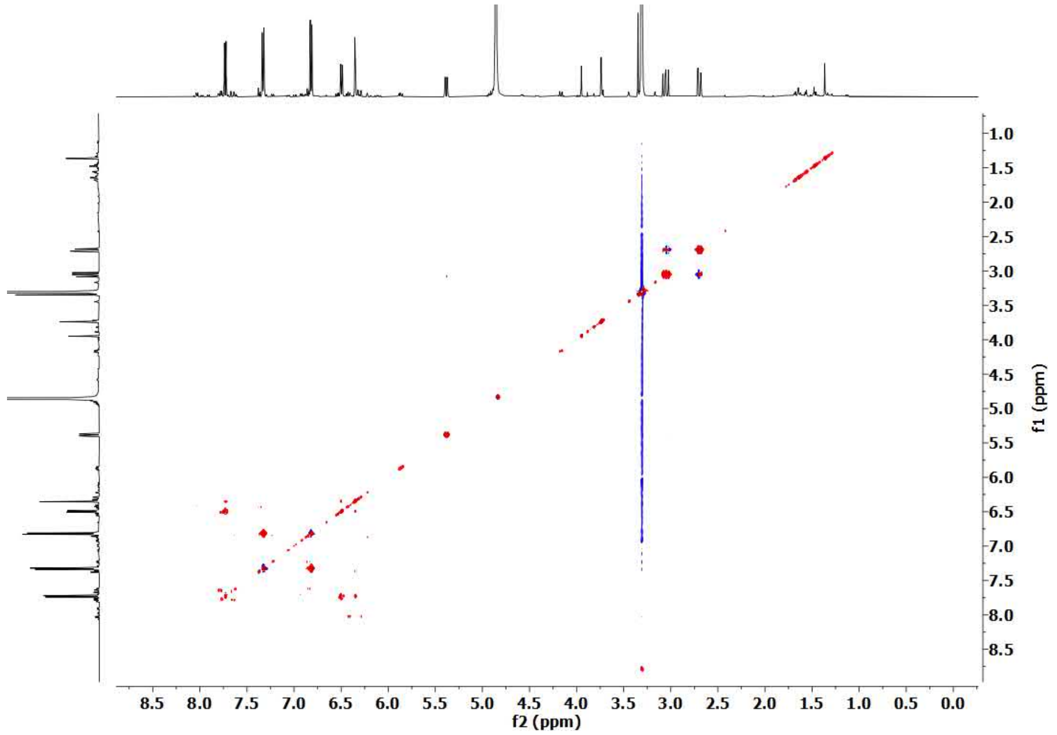

Figure S80. TOCSY (500 MHz, MeOH-d_4, 25 ºC) spectrum of 1-(2,4-dihydroxyphenyl)-3-hydroxy-3-(4-hydroxyphenyl)-1-propanone (**10**)

Spectroscopic Data of dalbinol (11)

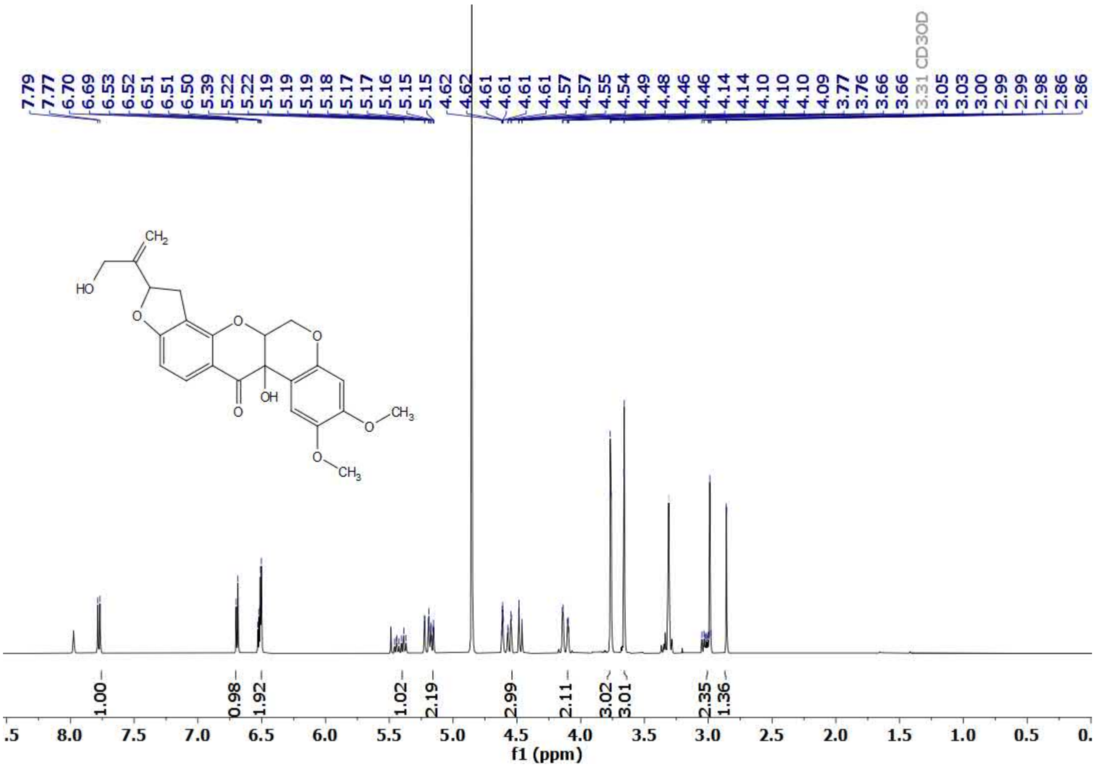

Figure S81. ^{1}H NMR (500 MHz, MeOH-d_4, 25 ºC) spectrum of dalbinol (**11**)

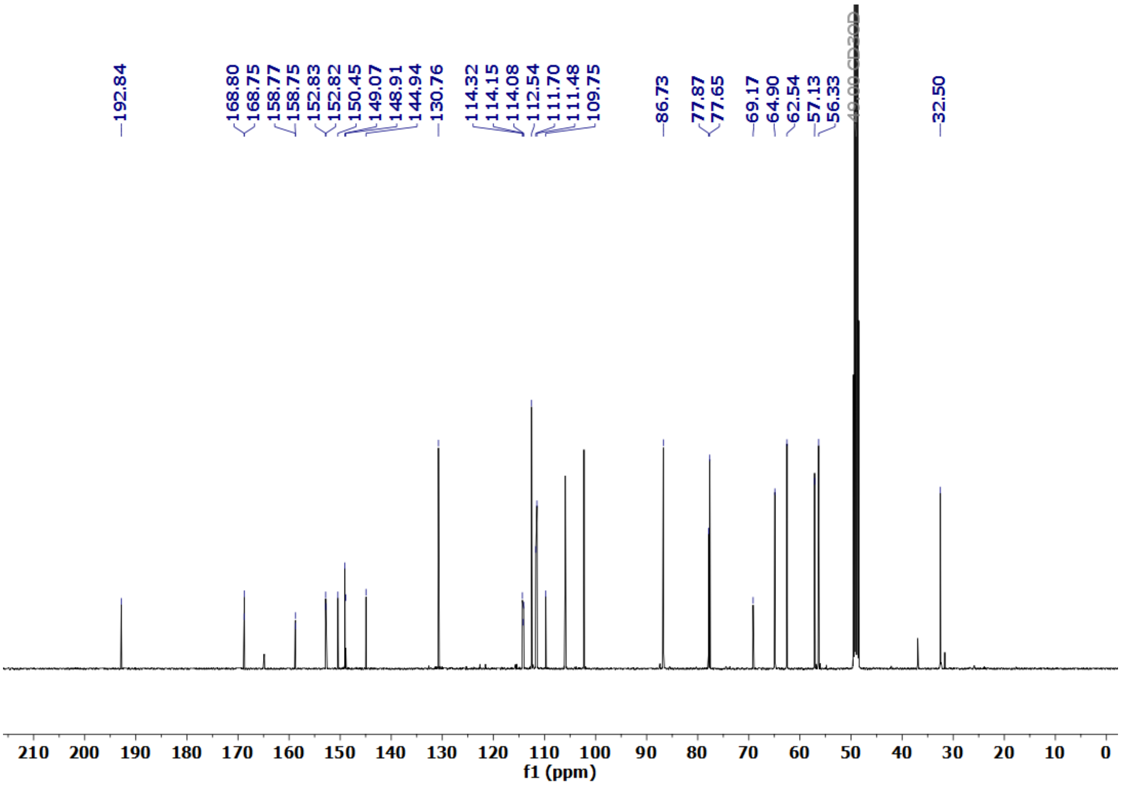

Figure S82. ^{13}C NMR (125 MHz, MeOH-d_4, 25 ºC) spectrum of dalbinol (**11**)

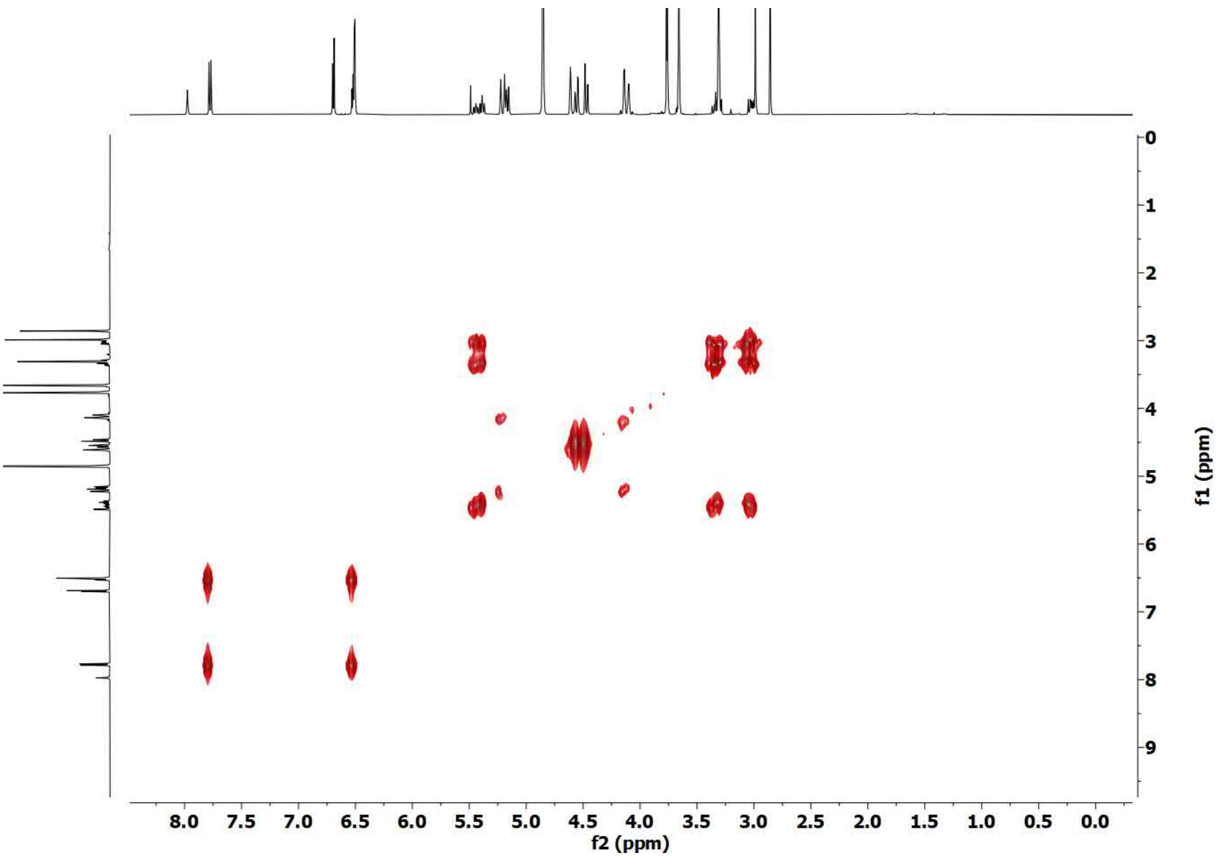

Figure S83. COSY (500 MHz, MeOH-d_4, 25 ºC) spectrum of dalbinol (**11**)

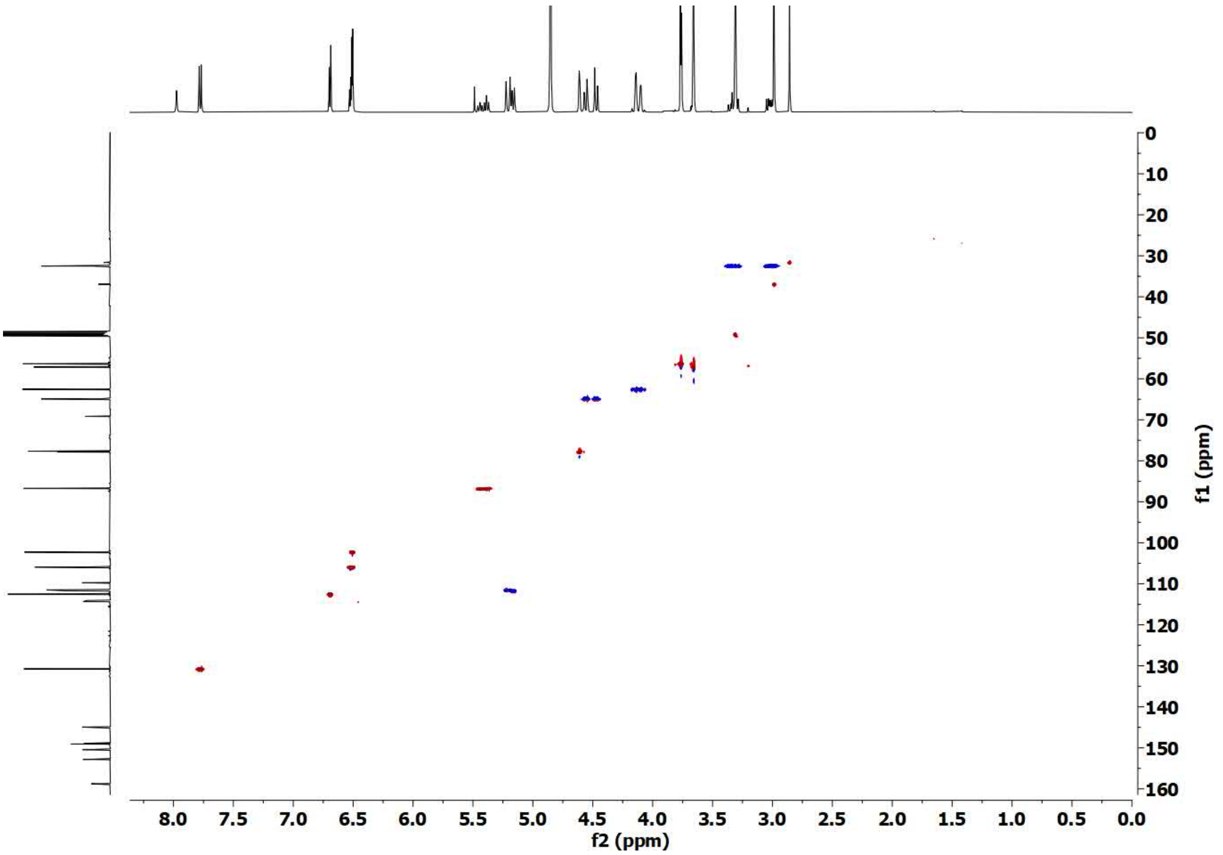

Figure S84. HSQC (500/125 MHz, MeOH-d_4, 25 ºC) spectrum of dalbinol (**11**)

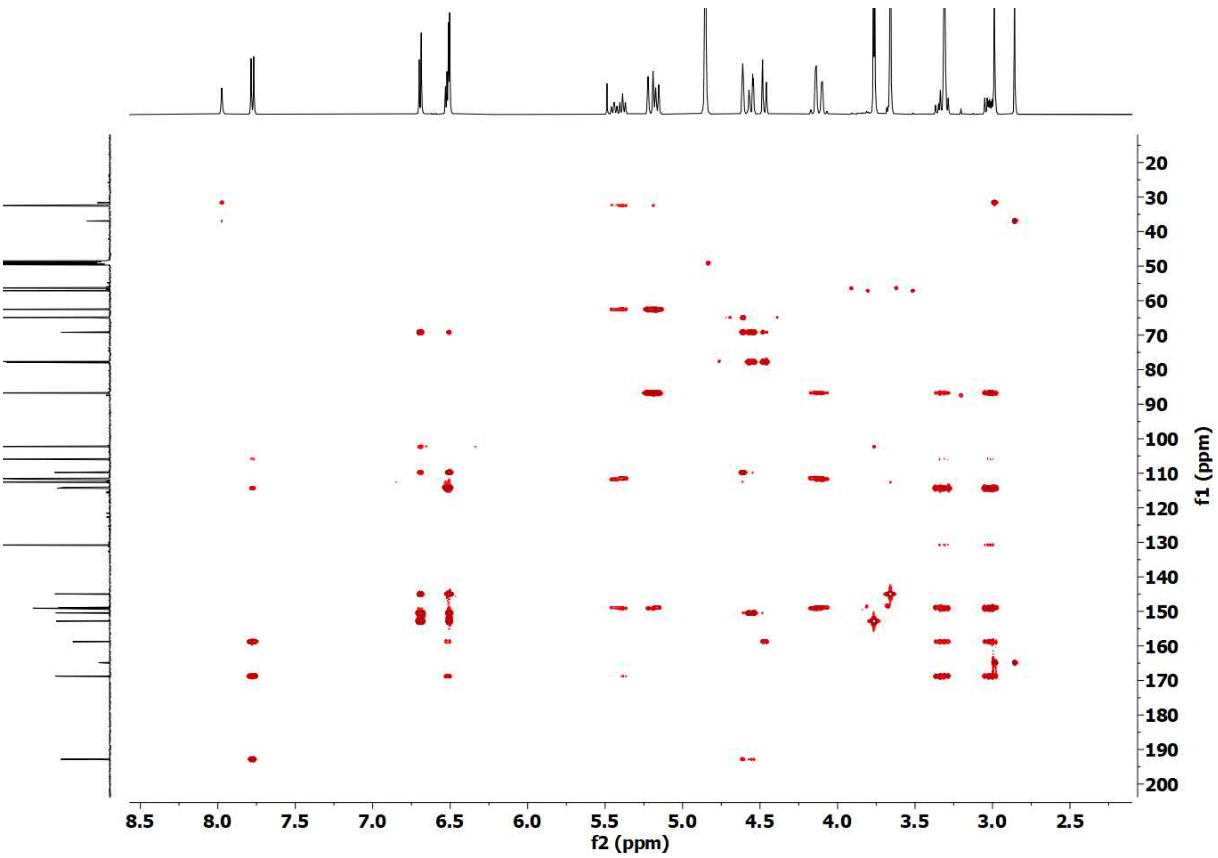

Figure S85. HMBC (500/125 MHz, MeOH-d_4, 25 ºC) spectrum of dalbinol (**11**)

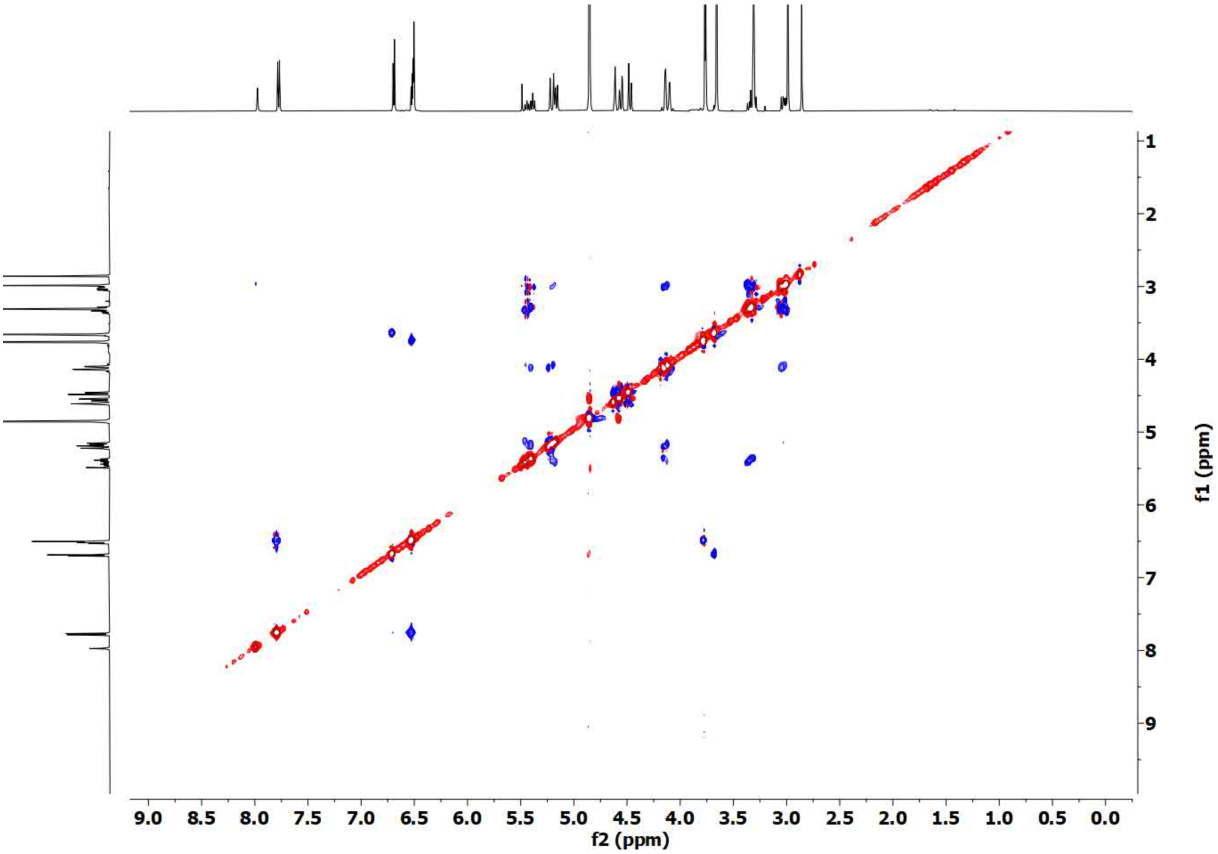

Figure S86. NOESY (500 MHz, MeOH-d_4, 25 ºC) spectrum of dalbinol (**11**)

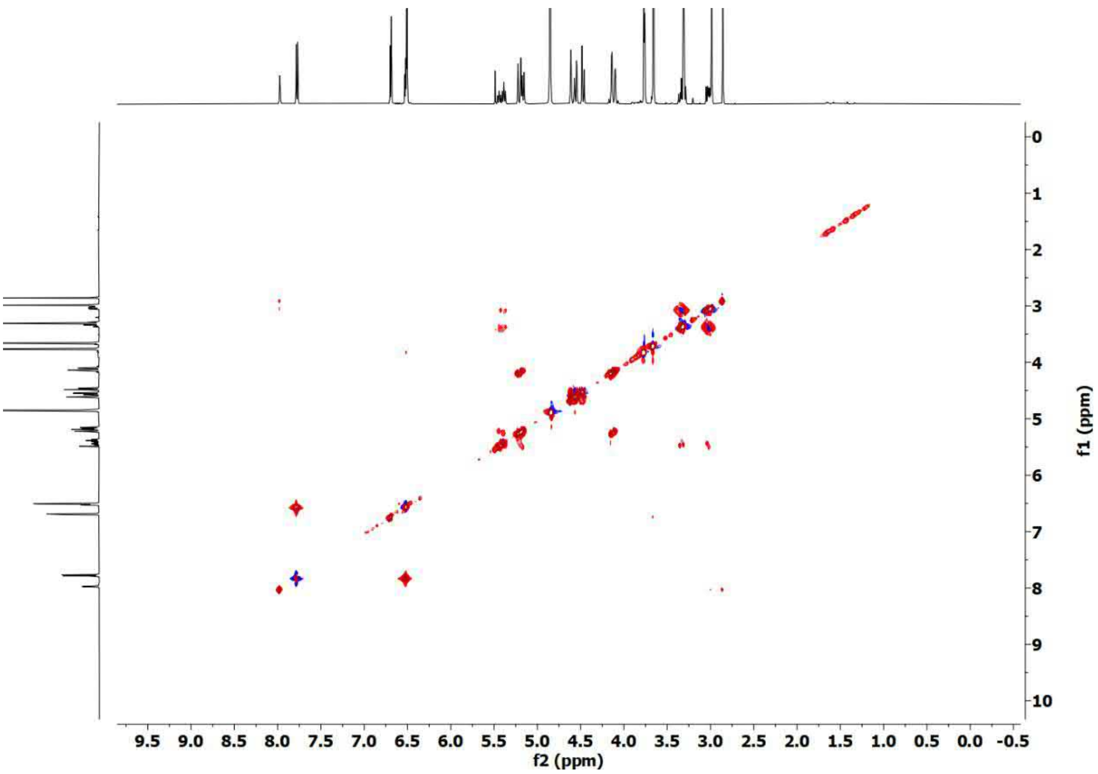

Figure S87. TOCSY (500 MHz, MeOH-d_4, 25 °C) spectrum of dalbinol (**11**)

Spectroscopic Data of (2*R*)-1,2-dihydro-2-[1-(hydroxymethyl)ethenyl]-8,9-dimethoxy[1]benzopyrano[3,4-*b*]furo[2,3-*h*][1]benzopyran-6(12*H*)-one (12)

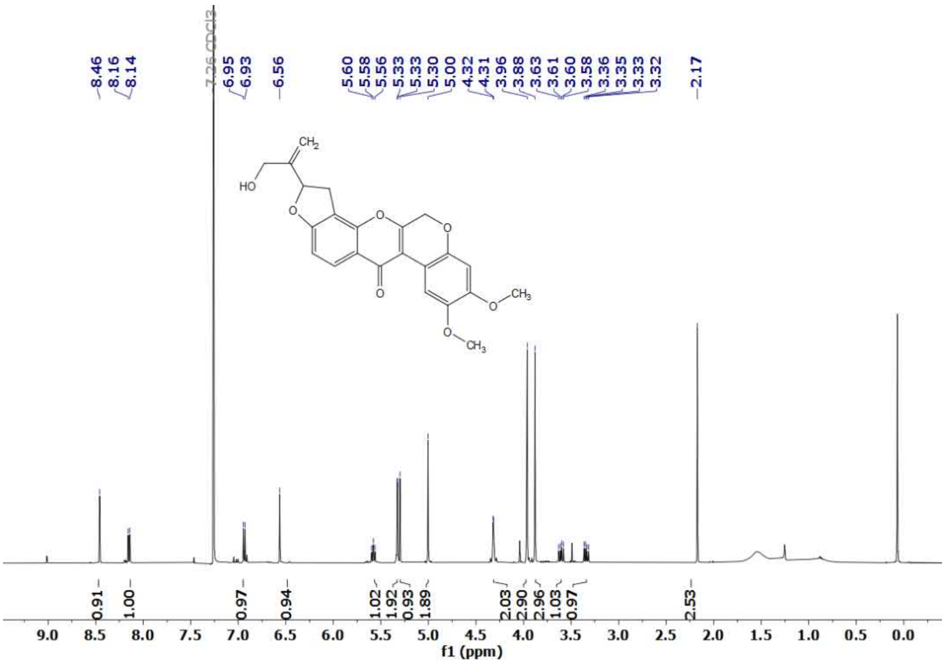

Figure S88. ^{1}H NMR (500 MHz, $CDCl_3$, 25 °C) spectrum of (2*R*)-1,2-dihydro-2-[1-(hydroxymethyl)ethenyl]-8,9-dimethoxy[1]benzopyrano[3,4-*b*]furo[2,3-*h*][1]benzopyran-6(12*H*)-one (**12**)

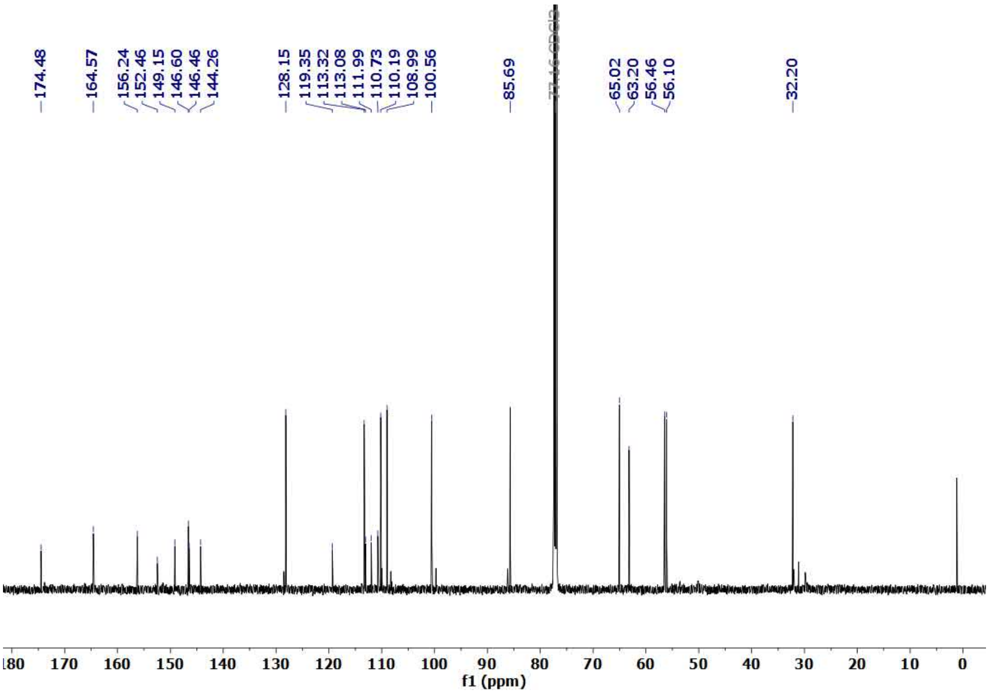

Figure S89. ^{13}C NMR (125 MHz, $CDCl_3$, 25 ºC) spectrum of (2*R*)-1,2-dihydro-2-[1-(hydroxymethyl)ethenyl]-8,9-dimethoxy[1]benzopyrano[3,4-*b*]furo[2,3-*h*][1]benzopyran-6(12*H*)-one (**12**).

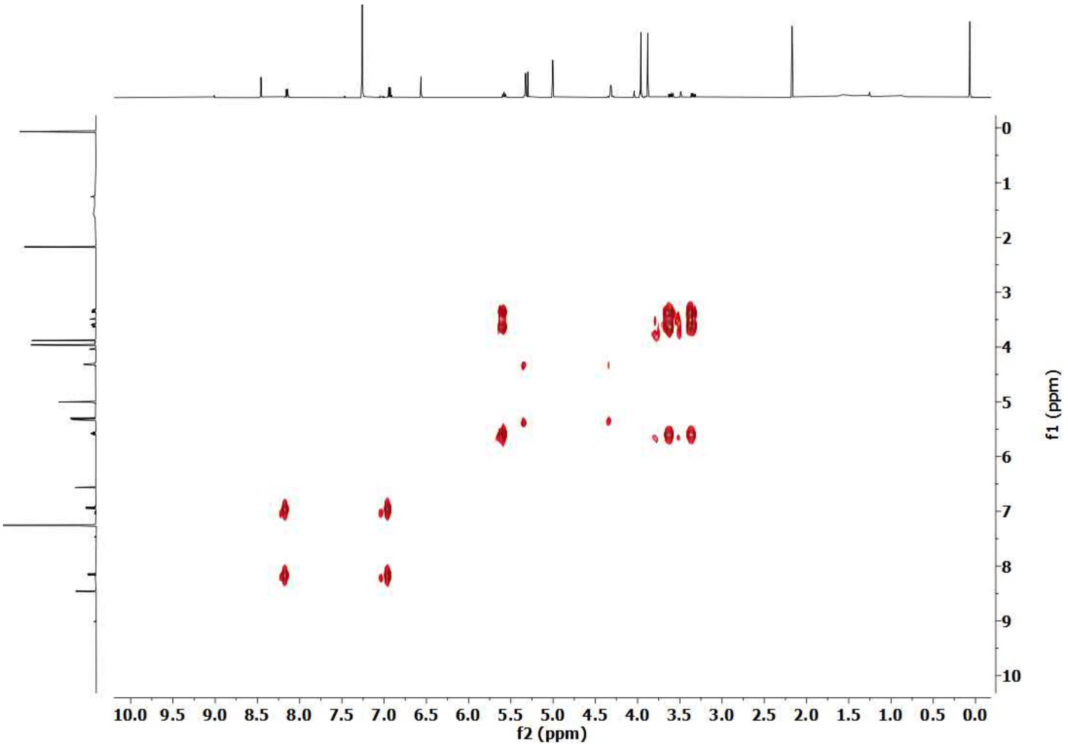

Figure S90. COSY (500 MHz, $CDCl_3$, 25 ºC) spectrum of (2*R*)-1,2-dihydro-2-[1-(hydroxymethyl)ethenyl]-8,9-dimethoxy[1]benzopyrano[3,4-*b*]furo[2,3-*h*][1]benzopyran-6(12*H*)-one (**12**).

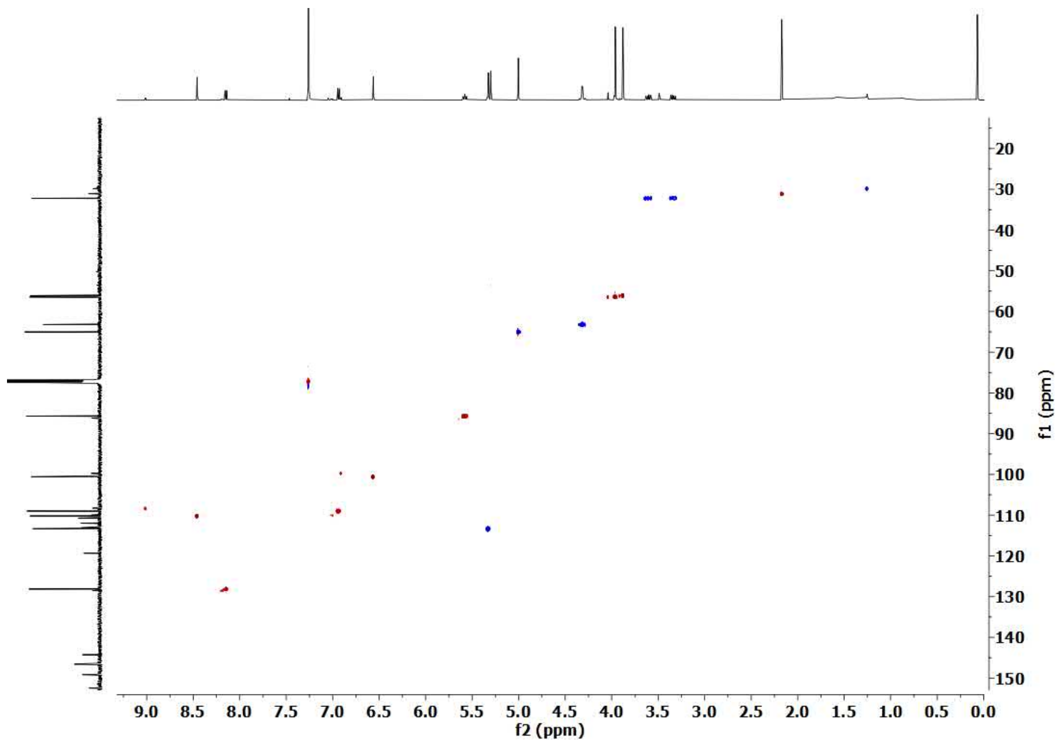

Figure S91. HSQC (500/125 MHz, $CDCl_3$, 25 ºC) spectrum of (2*R*)-1,2-dihydro-2-[1-(hydroxymethyl)ethenyl]-8,9-dimethoxy[1]benzopyrano[3,4-*b*]furo[2,3-*h*][1]benzopyran-6(12*H*)-one (**12**).

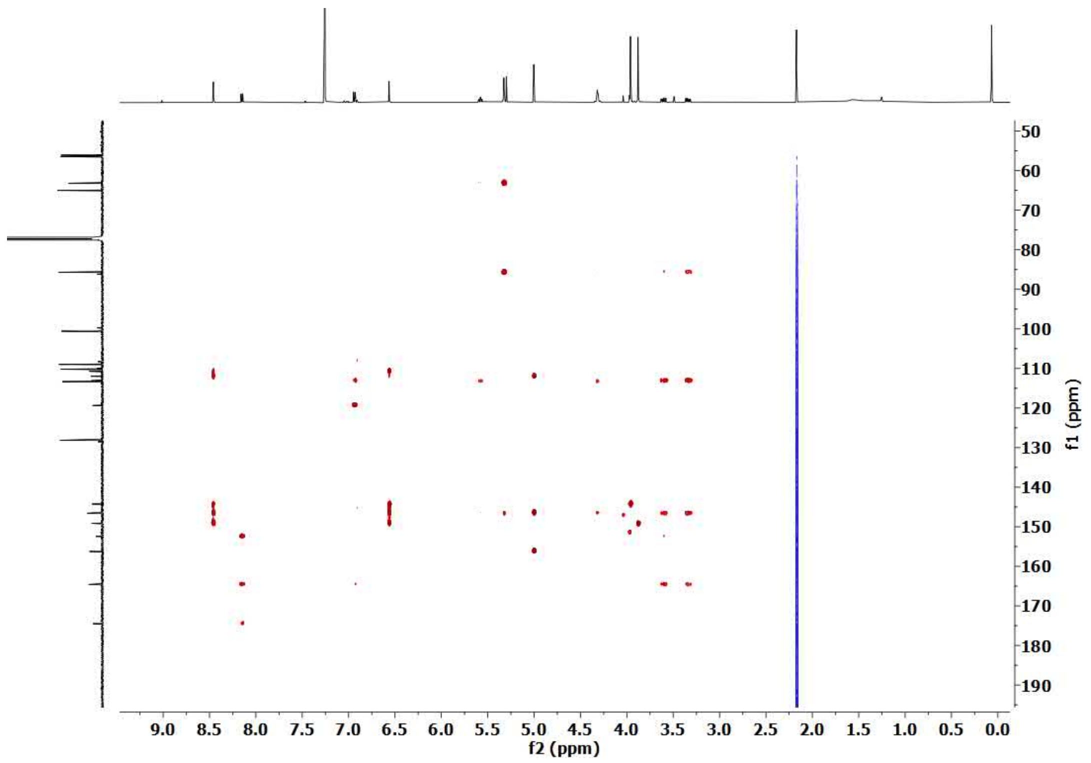

Figure S92. HMBC (500/125 MHz, $CDCl_3$, 25 ºC) spectrum of (2*R*)-1,2-dihydro-2-[1-(hydroxymethyl)ethenyl]-8,9-dimethoxy[1]benzopyrano[3,4-*b*]furo[2,3-*h*][1]benzopyran-6(12*H*)-one (**12**).

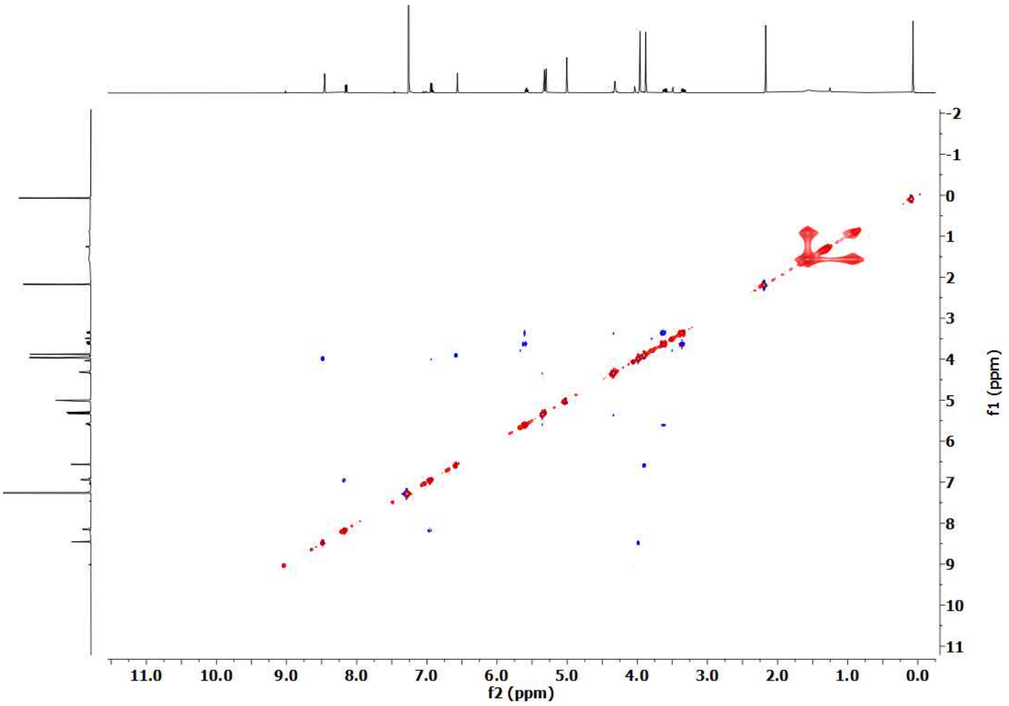

Figure S93. NOESY (500 MHz, $CDCl_3$, 25 ºC) spectrum of (2*R*)-1,2-dihydro-2-[1-(hydroxymethyl)ethenyl]-8,9-dimethoxy[1]benzopyrano[3,4-*b*]furo[2,3-*h*][1]benzopyran-6(12*H*)-one (**12**).

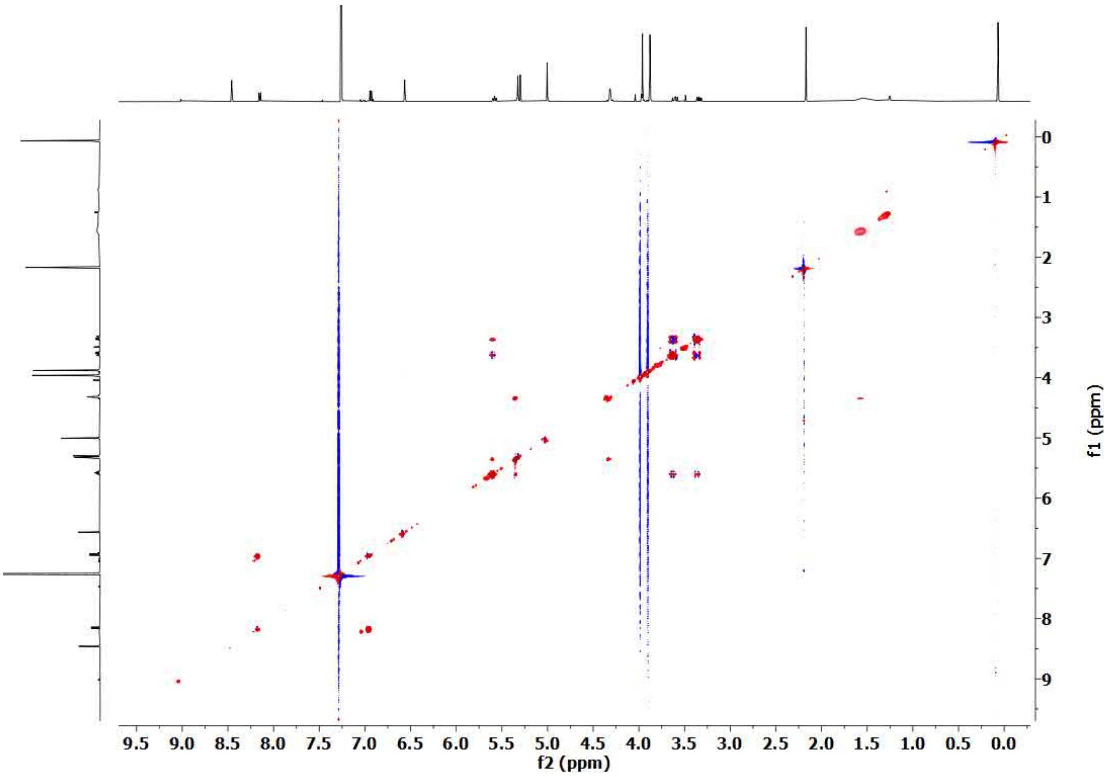

Figure S94. TOCSY (500 MHz, $CDCl_3$, 25 ºC) spectrum of (2*R*)-1,2-dihydro-2-[1-(hydroxymethyl)ethenyl]-8,9-dimethoxy[1]benzopyrano[3,4-*b*]furo[2,3-*h*][1]benzopyran-6(12*H*)-one (**12**).

Spectroscopic Data of oleanolic acid acetate (13)

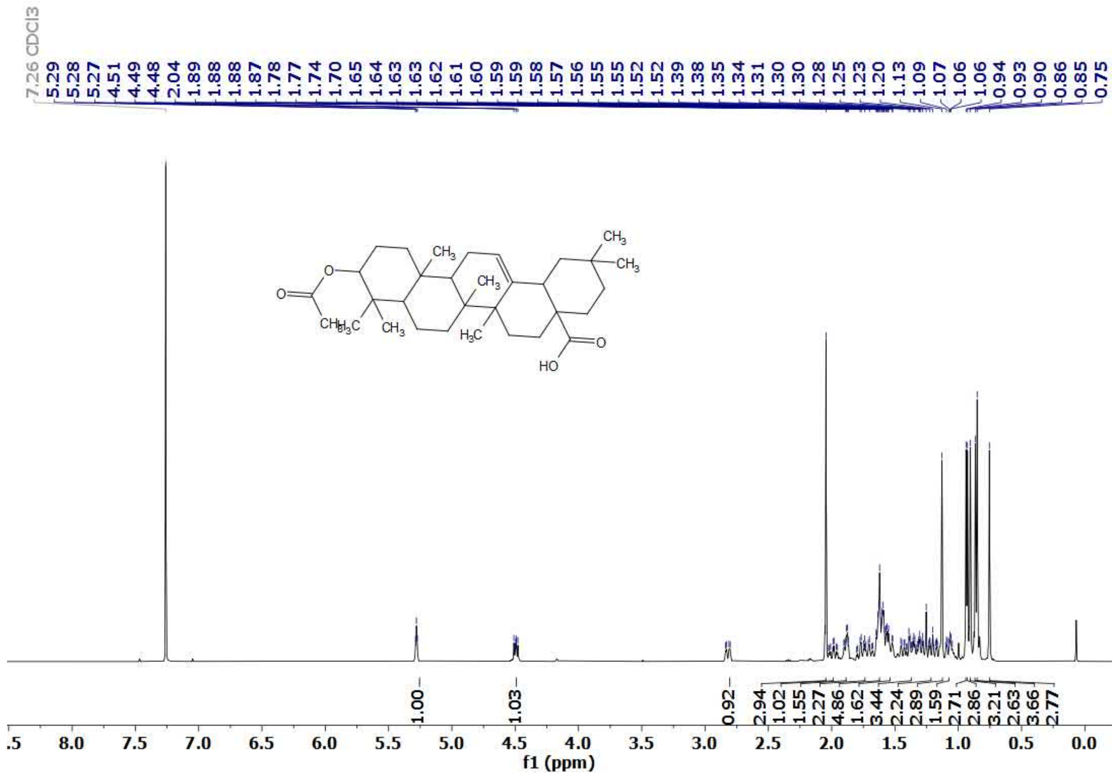

Figure S95. ^{1}H NMR (500 MHz, $CDCl_3$, 25 ºC) spectrum of Oleanolic acid acetate (**13**)

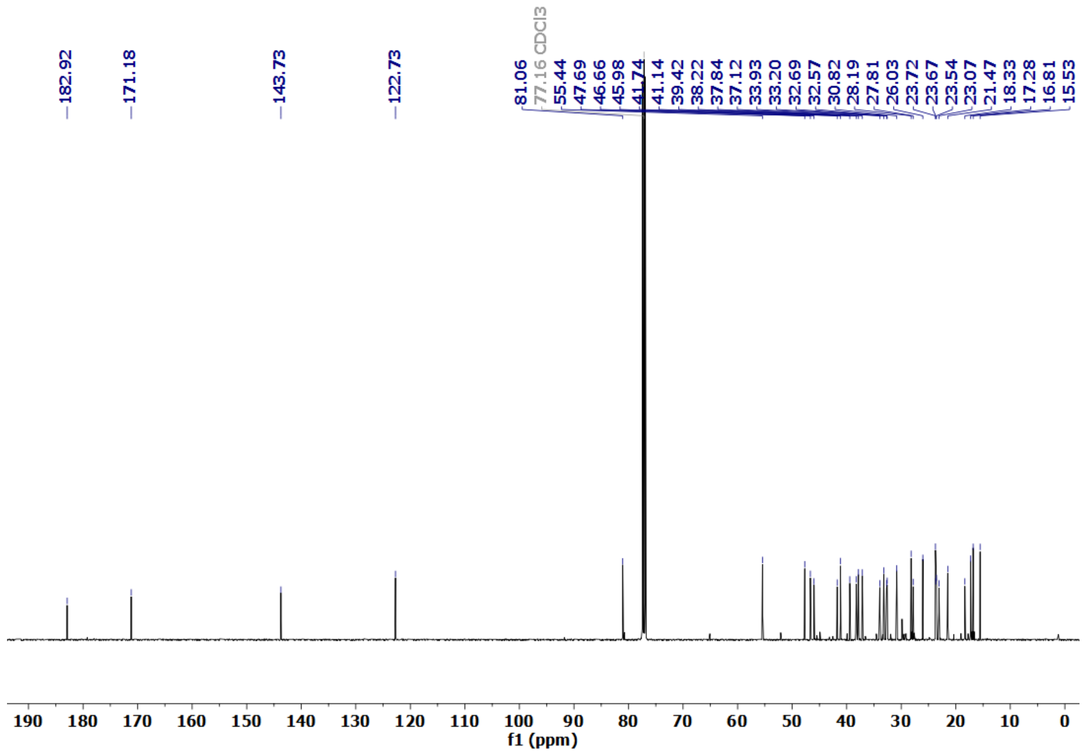

Figure S96. ^{13}C NMR (125 MHz, $CDCl_3$, 25 ºC) spectrum of Oleanolic acid acetate (**13**)

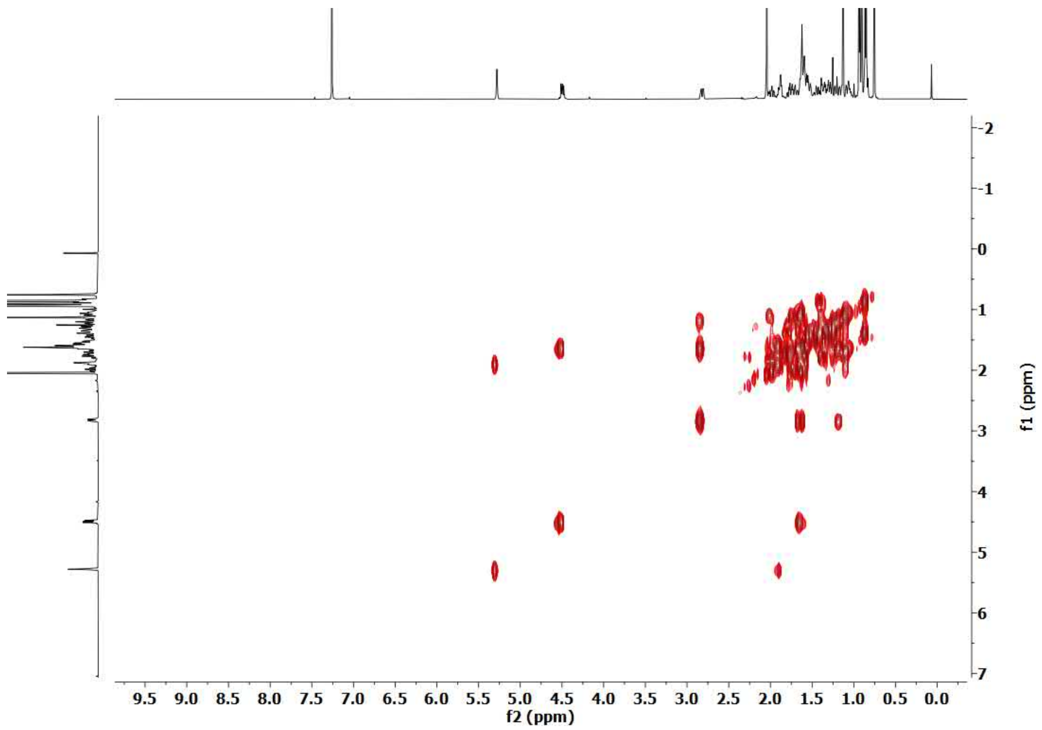

Figure S97. COSY (500 MHz, $CDCl_3$, 25 ºC) spectrum of Oleanolic acid acetate (**13**)

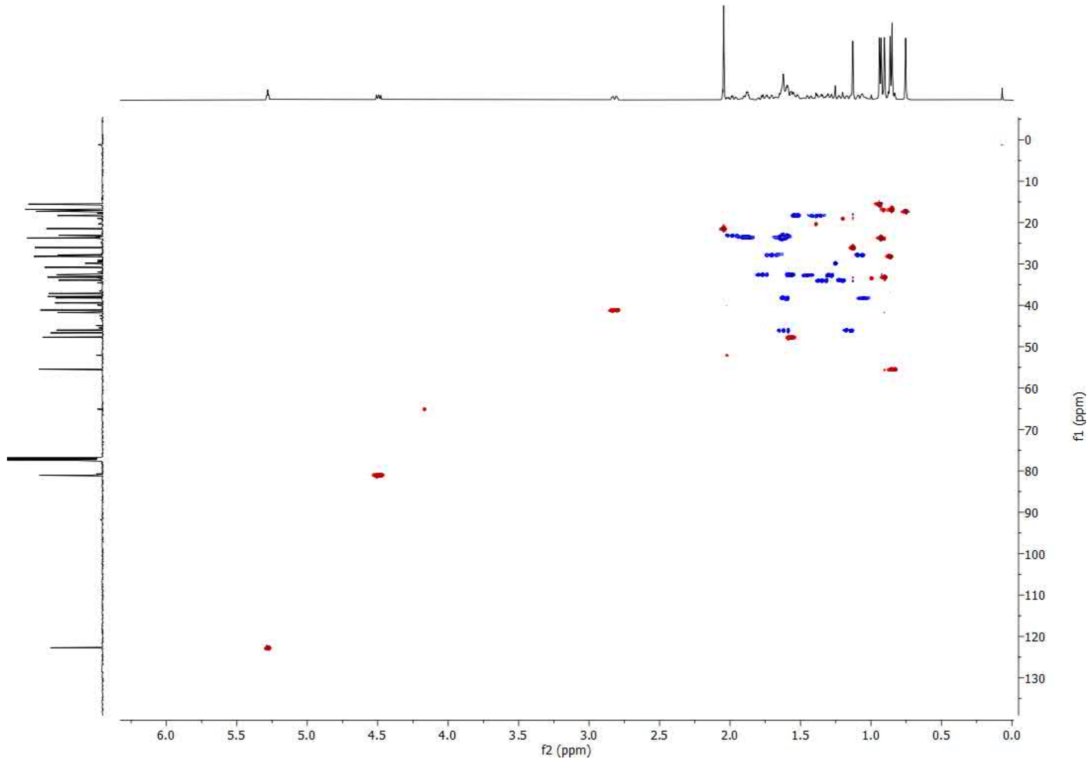

Figure S98. HSQC (500/125 MHz, $CDCl_3$, 25 ºC) spectrum of Oleanolic acid acetate (**13**)

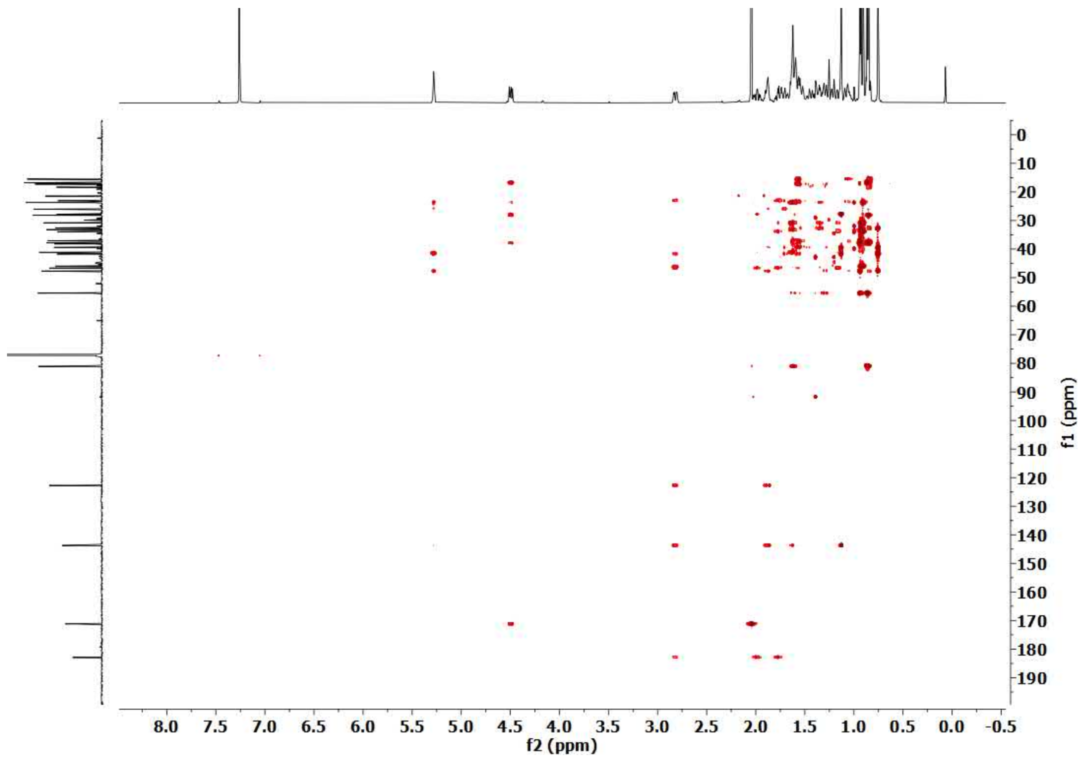

Figure S99. HMBC (500/125 MHz, $CDCl_3$, 25 ºC) spectrum of Oleanolic acid acetate (**13**)

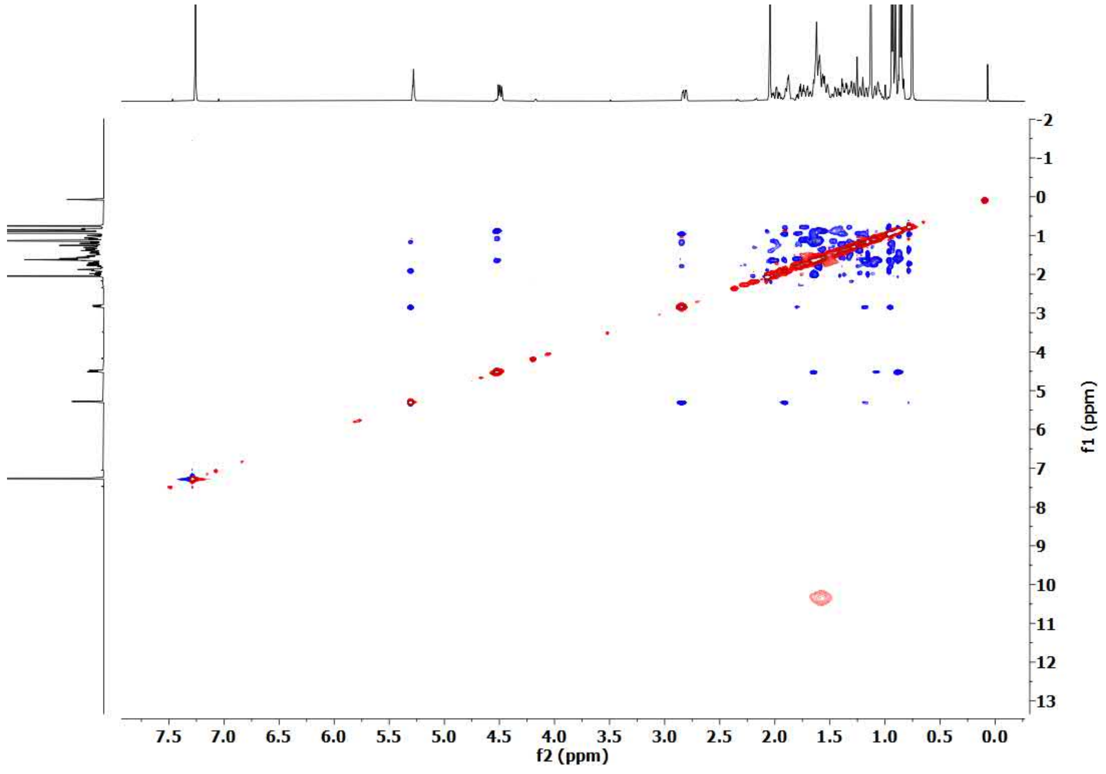

Figure S100. NOESY (500 MHz, $CDCl_3$, 25 ºC) spectrum of Oleanolic acid acetate (**13**)

ECD calculation

Table S1. Experimental ^{1}H and ^{13}C chemical shifts and calculated isotropic shieldings for the diastereosimers of **2**, **3** and **6** considered. For each compound, two diastereoisomers were considered with the unknown chiral center being *R* or *S*, respectively. For all diastereoisomers studied, also the calculated DP4(+) probabilities are included.

Compound **2**		Probability	100.00%	0.00%	
		**Exp**	**RRR (DP4+)**		**RRS (DP4+)**
C		85,500	109,300	109,400	
C		74,500	121,700	121,700	
C	x	194,400	4,700	4,700	
C	x	113,400	84,900	84,900	
C	x	130,100	66,600	66,600	
C	x	112,200	88,100	88,000	
C	x	166,900	34,500	34,500	
C	x	103,700	95,200	95,200	
C	x	165,100	33,900	33,800	
C	x	130,800	67,700	67,800	
C	x	116,400	83,600	83,700	
C	x	146,200	53,090	53,040	
C	x	142,200	57,480	57,430	
C	x	122,900	75,410	75,380	
C	x	118,300	79,860	79,710	
C	x	123,900	73,670	73,570	
C	x	131,200	66,640	66,690	
C		80,300	111,900	111,840	
C		42,100	153,650	153,550	
C		23,900	168,870	168,840	
C	x	125,300	72,280	72,180	
C	x	132,500	61,780	61,780	
C		25,900	169,230	169,130	
C		26,800	169,200	169,190	
C		17,800	177,800	177,730	
H		4,930	26,623	26,620	
H		4,480	26,872	26,854	
H	x	7,730	23,311	23,298	
H	x	6,540	24,699	24,700	
H	x	6,350	24,925	24,940	
H	x	6,890	24,207	24,189	
H	x	6,730	24,508	24,506	
H	x	6,400	24,728	27,733	
H	x	5,670	25,429	25,423	
H		1,790	29,940	29,840	
H		2,150	29,284	29,294	
H	x	5,120	26,019	26,009	
H		1,660	29,845	29,834	
H		1,420	30,014	30,015	
H		1,580	29,863	29,862	

**Compound 3**			Probability	88.68%	11.32%
		**Exp**	**RRR (DP4+)**		**RRS (DP4+)**
C		85,700	109,227	109,303	
C		74,500	121,805	121,858	
C	x	194,500	4,718	4,729	
C	x	114,200	85,125	85,150	
C	x	128,900	67,927	67,855	
C	x	112,200	86,470	86,507	
C	x	159,000	36,210	36,110	
C	x	110,400	87,291	87,514	
C	x	161,600	39,951	39,929	
C	x	129,900	68,020	68,187	
C	x	115,900	83,311	83,596	
C	x	146,400	54,773	54,764	
C	x	147,200	53,969	53,895	
C	x	116,100	83,586	83,660	
C	x	120,900	76,236	76,248	
C	x	117,000	80,221	79,999	
C	x	129,300	69,197	69,495	
C		81,500	111,304	111,334	
C		42,700	153,061	152,904	
C		23,900	169,232	168,748	
C	x	125,000	72,493	72,317	
C	x	132,700	61,674	61,761	
C		25,800	169,087	169,066	
C		27,400	168,509	168,242	
C		17,600	177,721	177,788	
H		4,990	26,591	26,621	
H		4,510	26,857	26,868	
H	x	6,600	24,703	24,690	
H	x	5,620	23,414	23,413	
H		1,390	30,141	30,159	
H		1,640	29,850	29,847	
H		1,560	29,883	29,895	
H	x	5,110	26,050	26,038	
H		2,100	29,367	29,395	
H	x	7,660	23,414	23,413	
H	x	6,500	24,703	24,690	
H	x	7,010	24,124	24,106	
H	x	6,820	24,301	24,304	
H	x	6,890	24,218	24,185	
H		1,740	29,900	29,894	

**Compound 6**			Probability	99.99%	0.01%
		**Exp**	**SR (DP4+)**	**SS (DP4+)**	
C		71,000	121,603	121,818	
H		3,990	27,334	27,339	
H		4,230	27,260	27,224	

C		33,800	155,267	155,241
H		3,460	28,052	28,071
C		31,400	158,802	159,207
H		2,930	28,554	28,448
H		2,830	28,625	28,715
C	x	114,800	81,981	82,005
C	x	131,200	67,178	67,208
H	x	6,880	24,220	24,214
C	x	109,000	91,932	91,884
H	x	6,320	24,877	24,874
C	x	157,600	43,269	43,265
C	x	103,800	96,424	96,418
H	x	6,220	24,965	24,967
C	x	156,400	42,791	42,819
C	x	123,500	74,619	74,526
C	x	144,600	56,162	56,241
C	x	139,700	60,720	60,748
C	x	148,200	53,159	53,115
C	x	131,600	65,977	66,439
C	x	117,700	77,811	77,509
H	x	6,480	24,670	24,667
C	x	110,600	87,263	87,720
H	x	4,960	26,053	26,089
H	x	4,890	26,121	26,143
C	x	149,800	47,444	47,439
H	x	6,160	24,752	24,769
C		44,900	142,630	142,844
C		41,200	150,016	150,270
H		1,960	29,508	29,538
H		1,650	29,878	29,878
C		24,700	164,637	164,925
H		1,780	26,682	26,677
H		1,650	29,823	29,841
C	x	126,200	71,492	71,511
H	x	5,040	26,074	26,080
C	x	131,700	62,568	52,588
C		25,880	164,782	165,057
H		1,630	29,872	29,881
C		25,910	165,755	165,530
H		1,340	30,170	30,169
C		17,700	173,421	173,655
H		1,490	29,970	29,977
C		60,200	130,922	131,173
H		3,740	27,816	27,825
